# Genetic heterogeneity and actionable mutations in HER2-positive primary breast cancers and their brain metastases

**DOI:** 10.18632/oncotarget.25041

**Published:** 2018-04-17

**Authors:** Leticia De Mattos-Arruda, Charlotte K. Y. Ng, Salvatore Piscuoglio, Maria Gonzalez-Cao, Raymond S. Lim, Maria R. De Filippo, Nicola Fusco, Anne M. Schultheis, Carolina Ortiz, Santiago Viteri, Alexandra Arias, Gabriel S. Macedo, Mafalda Oliveira, Patricia Gomez, Cristina Teixidó, Paolo Nuciforo, Vicente Peg, Cristina Saura, Santiago Ramon y Cajal, Francesc Tresserra Casas, Britta Weigelt, Javier Cortes, Joan Seoane, Jorge S. Reis-Filho

**Affiliations:** ^1^ Department of Pathology, Memorial Sloan Kettering Cancer Center, New York, NY, USA; ^2^ Vall d'Hebron Institute of Oncology (VHIO), Vall d'Hebron University Hospital, Barcelona, Spain; ^3^ Universitat Autònoma de Barcelona, Barcelona, Spain; ^4^ Institute of Pathology, University Hospital Basel, Basel, Switzerland; ^5^ Department of Biomedicine, University of Basel, Basel, Switzerland; ^6^ Quirón Dexeus University Hospital, Barcelona, Spain; ^7^ Vall d'Hebron Institute of Research, Vall d'Hebron University Hospital, Barcelona, Spain; ^8^ Ramon y Cajal University Hospital, Madrid, Spain; ^9^ Institució Catalana de Recerca i Estudis Avançats (ICREA), Barcelona, Spain; ^10^ Human Oncology and Pathogenesis Program, Memorial Sloan Kettering Cancer Center, New York, NY, USA

**Keywords:** metastatic breast cancer, HER2-positive, brain metastasis, actionable genetic alterations, personalized medicine

## Abstract

Brain metastases constitute a challenge in the management of patients with HER2-positive breast cancer treated with anti-HER2 systemic therapies. Here we sought to define the repertoire of mutations private to or enriched for in HER2-positive brain metastases. Massively parallel sequencing targeting all exons of 254 genes frequently mutated in breast cancers and/or related to DNA repair was used to characterize the spatial and temporal heterogeneity of HER2-positive breast cancers and their brain metastases in six patients. Data were analyzed with state-of-the-art bioinformatics algorithms and selected mutations were validated with orthogonal methods. Spatial and temporal inter-lesion genetic heterogeneity was observed in the HER2-positive brain metastases from an index patient subjected to a rapid autopsy. Genetic alterations restricted to the brain metastases included mutations in cancer genes *FGFR2, PIK3CA* and *ATR*, homozygous deletion in *CDKN2A* and amplification in *KRAS*. Shifts in clonal composition and the acquisition of additional mutations in the progression from primary HER2-positive breast cancer to brain metastases following anti-HER2 therapy were investigated in additional five patients. Likely pathogenic mutations private to or enriched in the brain lesions affected cancer and clinically actionable genes, including *ATR, BRAF, FGFR2, MAP2K4, PIK3CA, RAF1* and *TP53*. Changes in clonal composition and the acquisition of additional mutations in brain metastases may affect potentially actionable genes in HER2-positive breast cancers. Our observations have potential clinical implications, given that treatment decisions for patients with brain metastatic disease are still mainly based on biomarkers assessed in the primary tumor.

## INTRODUCTION

Brain metastases represent a frequent source of morbidity and mortality for breast cancer patients [1]. The incidence of brain metastasis in patients with metastatic breast cancer varies from 10 to 15% [2] and these rates are as high as 50% in patients with the Erb-B2 receptor tyrosine kinase 2 (*ERBB2* or HER2)-positive breast cancer [3].

The central nervous system (CNS) remains a sanctuary site for HER2-positive breast cancer [4, 5]. Whilst anti-HER2 targeted therapies have resulted in better therapeutic control for systemic disease [6–8], CNS metastases often occur [9]. Several hypotheses may explain this clinical phenomenon, including the poor or non-penetration of trastuzumab, a recombinant humanized anti-HER2 monoclonal antibody, across the blood-brain barrier, better imaging methods for the diagnosis of brain metastasis and the increased life expectancy of HER2-positive breast cancer patients with newer anti-HER2-targeted therapies [10].

Yet there is a limited understanding of how brain metastases evolve from their primary HER2-positive breast cancers and how they can be effectively targeted in clinical practice. In this study we hypothesized that clinically metachronous brain metastasis from HER2-positive breast cancers would differ in their repertoire of somatic genetic alterations from their respective primary tumor, and that potentially targetable driver genetic alterations would be enriched in or restricted to the metastases, and could be employed as genetic biomarkers to guide the rational use of targeted agents. The aims of this study were i) to define the repertoire of somatic genetic alterations in primary HER2-positive breast cancers and their corresponding brain metastases in patients whose lesions metastatic to the brain were collected at rapid post-mortem examination or surgical excision of the brain metastases, ii) to analyze the temporal heterogeneity involved in the progression of HER2-positive breast cancers to brain metastasis, and iii) to identify potential clinically actionable alterations that may allow targeting brain metastasis in HER2-positive breast cancer patients.

## RESULTS

### The genetic diversity of a brain metastasis from a HER2-positive breast cancer patient subjected to a rapid post-mortem analysis

**Figure 1:**
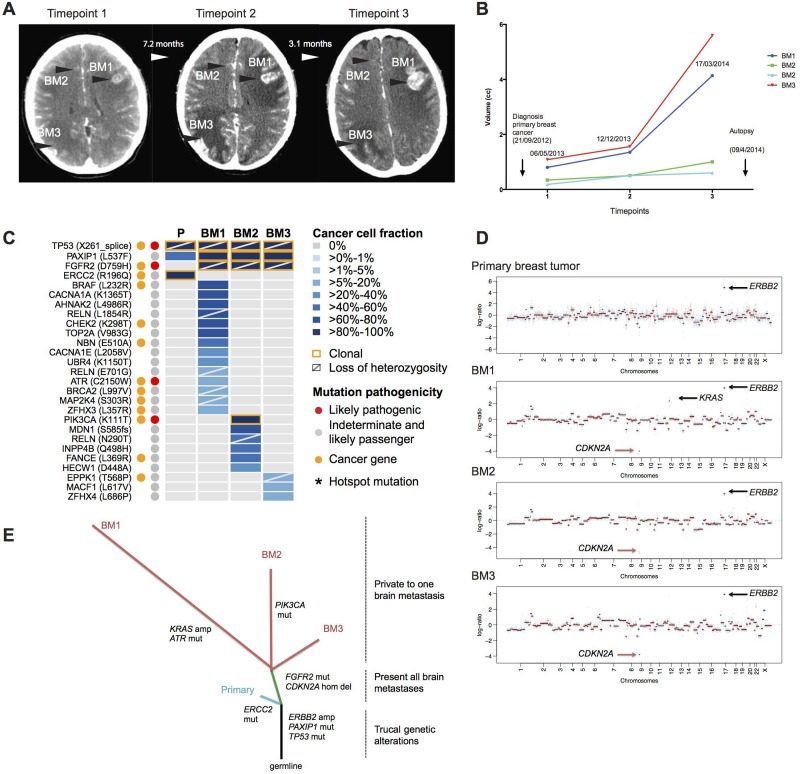
The somatic genetic alterations in the primary breast tumor and the three synchronous, spatially distinct brain metastatic lesions of the index case (**A**) Longitudinal representation of computer tomography scans, showing coronal sections in T1W1 and the presence of 3 main lesions: BM1, left frontal lobe, BM2, basilar ganglion, BM3, right occipital lobe during the timepoint 1 (06.05.2013), timepoint 2 (12.12.2013) and timepoint 3 (17.03.2014). (**B**) Bar graphs representing the volumes of main brain lesions that were followed up in the clinics. Note that two small lesions in the basilar ganglion (BM2) were followed up in the clinical care of this patient. (**C**) Heatmap depicting the cancer cell fractions (CCF) of the mutations as defined by ABSOLUTE [11] in the primary breast cancer and the metachronous brain metastases. Color key for CCF is depicted. Red and orange dots indicate likely pathogenic mutations and mutations affecting cancer genes [12–14], respectively. Asterisk (^*^) indicate hotspot mutation [17]. The presence of loss of heterozygosity is represented by a diagonal bar, and clonal mutations are indicated by an orange box. (**D**) Genome plots of the primary tumor and the three brain metastatic lesions highlighting *ERBB2* gene amplification, *KRAS* amplification and *CDKN2A* homozygous deletion. Smoothed Log_2_ ratios were plotted on the y-axis according to their genomic positions indicated on the x-axis. (**E**) Phylogenetic tree constructed from the somatic mutations, amplifications and homozygous deletions highlighting the main genetic alterations for each sample sequenced. The branch lengths are proportional to the number of genetic alterations.

The index patient was a 36-year-old female with an ER-negative, HER2-positive invasive ductal carcinoma and associated liver metastasis (cT3N3M1) at presentation in 2012. The patient received first-line therapy with the anti-HER2 trastuzumab plus paclitaxel; following successive visceral and brain progression (7.5 months after diagnosis), the patient received whole brain radiotherapy and three additional lines of anti-HER2 therapies combined with cytotoxic and/or targeted agents i) capecitabine, trastuzumab, BKM120; ii) lapatinib, capecitabine, iii) vinorelbine, trastuzumab. The patient expired 18.6 months after the initial diagnosis and was subjected to a rapid post-mortem examination (Figure 1A and 1B and Table 1). In the final radiological assessment ∼21 days before death, the patient had no measurable systemic extra-cranial visceral metastases (the liver metastasis was in clinical complete remission) except for the supra and infratentorial anatomically distinct brain metastases that were longitudinally followed up from the diagnosis of the CNS infiltration to the post-mortem analysis (Figure 1A and 1B).

**Table 1:** Clinico-pathologic characteristics and outcome of the six patients included in the study

Patient ID	Breast cancer subtype	Histologic subtype	Age at diagnosis (years)	Anti-HER2 therapy administered between the diagnosis of the primary breast cancer and the collection of the brain metastases	Time to brain metastasis diagnosis (months)	Extra-cranial disease	Overall survival (months)
**Index**	ER-negative/HER2-positive	IDC	36	Trastuzumab plus taxanes	7.5	No extra-cranial disease at the last clinical assessment before death	18.6
**Case 1**	ER-negative/HER2-positive	ILC	44	Neo (adjuvant): anthracycline and taxanes-based plus trastuzumab	26.3	Bone metastasis in lumbar vertebrae (L4) synchronous to brain metastasis	67
**Case 2**	ER-positive/HER2-positive	IDC	40	Adjuvant: anthracycline and taxanes-based plus endocrine therapy. No adjuvant trastuzumab due to low ventricular ejection fraction	26.1	Axillary lymph node and leptomeningeal carcinomatosis *a posteriori* to the diagnosis of brain metastasis	58.3
**Case 6**	ER-positive/HER2-positive	IDC	30	Adjuvant: anthracycline and taxanes-based plus trastuzumab and endocrine therapy. Metastatic setting: Capecitabine plus vinorelbine	39	Bone lesion resected, then irradiated	52
**Case 12**	ER-positive/HER2-positive	IDC	40	Neo (adjuvant): anthracycline and taxanes-based plus adjuvant chemotherapy followed by trastuzumab and endocrine therapy	18.1^*^	No	62.6
**Case 14**	ER-negative/HER2-positive	IDC	52	Neoadjuvant: anthracycline and taxanes-based plus trastuzumab	19.5	No	27.6

ER, estrogen receptor; IDC, invasive ductal carcinoma; ILC, invasive lobular carcinoma.

^*^Time to the first brain metastasis.

The primary tumor and three spatially separated brain metastases were subjected to high-depth targeted sequencing of 254 genes frequently mutated in breast cancer and/or related to DNA repair (Supplementary Table 1) to 238× in the primary tumor, a median depth of 1,267× (range: 1,056×–1,503×) in the metastases and to 1,109× in the matched normal sample (Supplementary Table 2). All mutations found by targeted sequencing where genomic DNA was available were subjected to orthogonal validation using amplicon resequencing (Supplementary Tables 3 and 4).

We sought to define whether intra-tumor genetic heterogeneity would be present between the primary breast cancer and the brain metastatic deposits, and whether the progression from HER2-positive primary breast cancer metastatic to the brain following anti-HER2 therapy was associated with shifts in clonal composition. Overall, the primary breast cancer and the three synchronous and spatially distinct brain metastatic lesions, in addition to displaying a clonal *ERBB2* (*HER2*) gene amplification, harbored 27 somatic mutations (Figure 1C). The likely pathogenic *TP53* X261_splice mutation was clonal (as defined by ABSOLUTE [11]) and ubiquitously present in the primary tumor and in the three brain metastatic deposits. The *PAXIP1* L537F mutation was subclonal in the primary tumor and expanded to become clonal in the brain metastases. Among the 27 somatic mutations, 24 were restricted to the brain metastases, of which 4 affected cancer-related genes (i.e., *FGFR2, MAP2K4, ATR* and *PIK3CA*) (Figure 1C) [12–16]. The likely pathogenic *FGFR2* D759H missense mutation was clonal in the three metastatic deposits found in the patient. Most of the somatic mutations identified were private to one of the metastatic deposits and the likely pathogenic hotspot *PIK3CA* K111T mutation, a recurrently mutated residue in breast cancer [16], was private to and clonal in the brain metastasis #2 (BM2).

Analysis of the CNAs demonstrated that *ERBB2* amplification was present both in the primary tumor and all brain metastases; reinforcing its role as an early ‘truncal’ event (Figure 1D). By contrast, we observed a homozygous deletion on chromosome 9p21.3 in the three brain metastases but not in the primary breast cancer. This locus encompasses the *CDKN2A* (p16) gene, an important tumor suppressor gene with a central role in cell cycle regulation. Furthermore, the brain metastasis #1 (BM1) showed a private (i.e., not present in the primary tumor or in the other synchronous brain metastases) amplification on chromosome 12p12.1, encompassing the *KRAS* gene.

A phylogenetic analysis of the somatic mutations and CNAs of the index patient suggested that the brain metastatic lesions diverged from the primary tumor and acquired additional likely pathogenic mutations in the cancer genes *FGFR2, PIK3CA* and *ATR*, homozygous deletion in *CDKN2A* and amplification in *KRAS* (Figure 1E). This led us to expand our cohort to investigate temporal heterogeneity in pairs of HER2-positive primary breast tumor and brain metastases.

### Characterization of somatic genetic alterations of primary breast cancers and their metachronous brain metastases in five additional patients

Sequencing of an additional five primary HER2-positive breast cancers and corresponding six brain metastases was performed to median depths of 447× (range: 84×–645×), 392× (range: 139×–1,148×) and 467× (range: 196×–1,790×) in the primary tumors, brain metastases and matched normal counterparts, respectively (Table 1, Supplementary Table 2).

**Figure 2:**
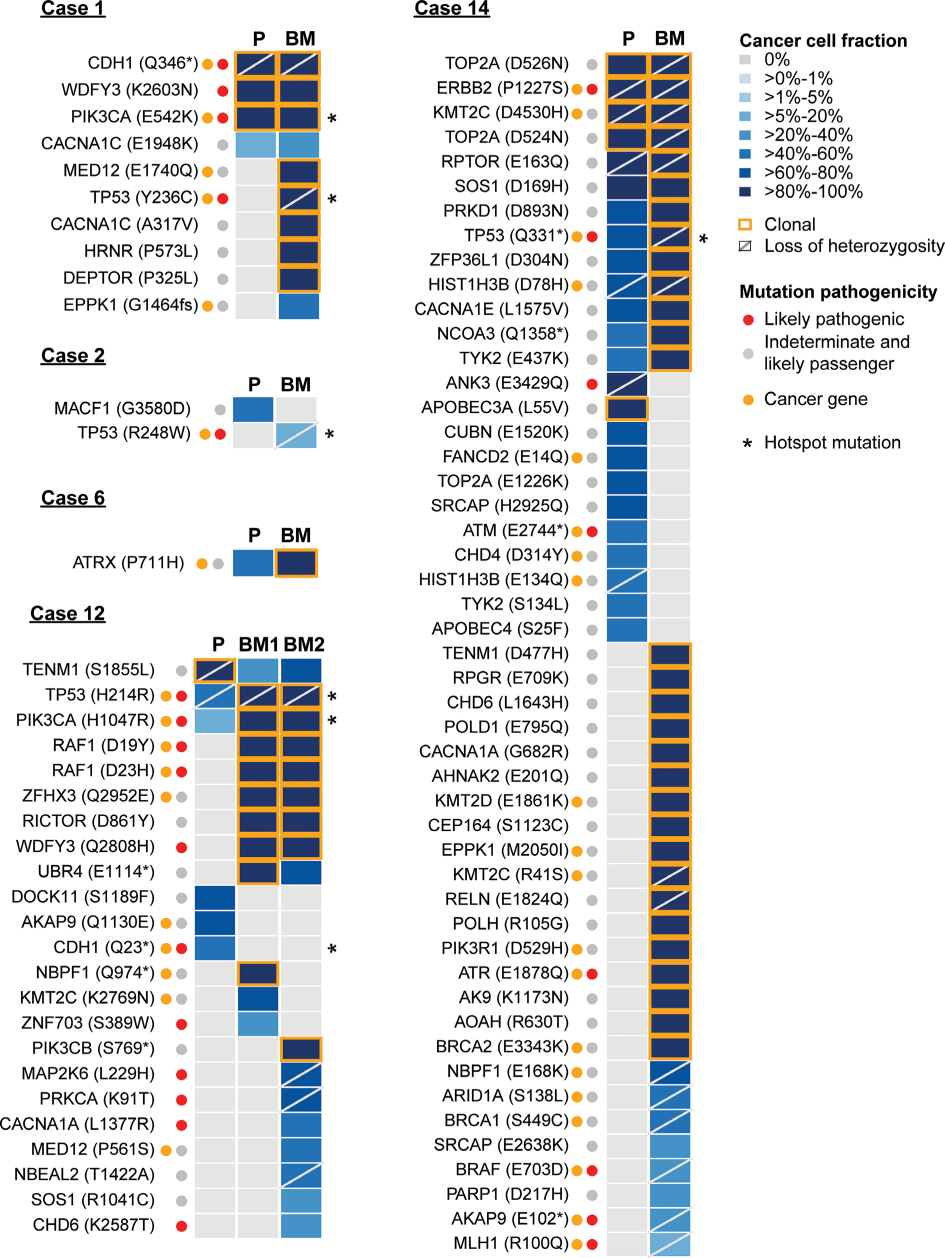
The repertoire of somatic genetic alterations in primary breast cancers and their respective brain metastases Heatmap depicting the cancer cell fractions (CCF) of the mutations as defined by ABSOLUTE [11] in the primary breast tumors and the metachronous brain metastases. CCFs are depicted according to the color key. Red and orange dots indicate likely pathogenic mutations and mutations affecting cancer genes [12–14], respectively. Asterisks (^*^) indicate hotspot mutations [17]. The presence of loss of heterozygosity (LOH) of the wild-type allele is represented by a diagonal bar, and mutations considered clonal by ABSOLUTE [11] are indicated by an orange box.

Across the five cases, the primary tumors and the metastases harbored a median of 3 (range: 1–24) and 10 (range: 1–38) somatic mutations, respectively (Supplementary Table 4), with a higher number of somatic mutations in the brain metastases than the corresponding primary tumors (*P* = 0.02, paired Mann-Whitney *U*-test, Figures 1C and 2). Overall, 111 somatic mutations targeting 73 genes were detected (median 16.5 per case, range 1–49), including 18 likely pathogenic mutations in 11 cancer genes (*AKAP9, ATM, ATR, BRAF, CDH1, ERBB2, FGFR2, MLH1, PIK3CA, RAF1* and *TP53*, Figures 1C and 2). A pairwise comparison revealed that a median of 17% (range: 0%–100%) of the mutations were shared by the primary tumors and corresponding brain metastases, while a median of 15% (range: 0%–22%) and 60% (range: 0%–88%) were restricted to the primary tumors and metastases, respectively (Figures 1C and 2). Hotspot mutations [17] present in both the primary tumor and brain metastasis were identified in Cases 1 (*PIK3CA* E542K) and 12 (*TP53* H214R and *PIK3CA* H1047R, Figure 2). Most CNAs were present in both the primary tumor and the brain metastases (Supplementary Figure 2), consistent with the notion that CNAs are early events in breast cancer tumorigenesis, likely constituting punctuated evolutionary bursts [18]. Importantly, *ERBB2* amplification was present and inferred by FACETS [19] to be clonal in all primary tumors and their respective brain metastases, therefore likely represented a ‘truncal’ genetic event. Further focal amplifications present in both the primary tumors and their respective brain metastases affected 19q13.33 (encompassing *NR1H2* and *POLD1*, Case 2) and 12q13.2–15 (*ERBB3*, *CDK4* and *MDM2*, Case 6).

Of particular interest are the 61 genes affected by mutations selected for in the brain metastases, including those mutations private to, enriched in, or associated with secondary LOH event in the brain metastasis. These genes included 54 genes affected by 73 mutations that were private to the brain metastases, 31 of which that were clonal in at least one of the brain metastases analyzed, including two hotspot mutations in *TP53* (Case 1, Y236C and Case 2, R248W), as well as seven likely pathogenic mutations in *ATR*, *FGFR2*, *PIK3CA*, *RAF1*, *TP53,* and *WDFY3* (Figures 1C and 2). We further identified 11 genes affected by 12 mutations that were enriched in the brain metastasis, including pathogenic hotspot mutations in *PIK3CA* (Case 12, H1047R) and *TP53* (Case 12, H214R). Two genes (*TOP2A* and *TP53*) were affected by three shared mutations that were associated with a secondary LOH event in the metastases but not in the primary tumor in Case 14, including a *TP53* Q331* truncating mutation. Collectively, the mutations private to, enriched in, or associated with a secondary LOH event in the brain metastasis affected 61 genes. Additionally, the index case harbored copy number alterations restricted to the brain metastases (*KRAS* amplification in brain metastasis #1, *CDKN2A* homozygous deletion in all three brain metastases) (Figure 1D).

Mutational processes that shape the human genome can be deduced from the pattern of somatic mutations detected in cancer cells [20]. For Case 14, the high mutation burden allowed us to identify signatures associated with increased APOBEC cytidine deaminase activity (2 and 13 [20]) as the predominant signatures among the mutations shared between the primary tumor and the brain metastasis and among the mutations private to the metastasis (Supplementary Figure 1). Mutations private to the primary breast cancer were associated with signature 2 (APOBEC) and 3 (homologous recombination deficiency), although the number of mutations private to the primary was small and the mutations were not associated with an increased burden of small insertions and deletions. In the remaining cases, this analysis was not possible, as the tumors harbored < 20 somatic mutations.

Taken together, these results suggest that primary HER2-positive breast cancers and their respective metachronous brain metastases displayed important genetic differences that affected cancer genes. Of note, four of the six cases harbored *TP53* likely pathogenic mutations (three hotspot mutations and one truncating mutation) that were private to or enriched in the brain lesions, all of which were associated with LOH of the wild-type allele in the metastatic deposit.

### Tumor progression from the breast cancer to the brain site is underpinned by temporal genetic heterogeneity

The tissue samples in Case 12 were longitudinally collected, thus we sought to define whether shifts in clonal composition would take place in the progression from the primary tumor to the brain metastases in this HER2-positive breast cancer following anti-HER2 therapy (Table 1).

**Figure 3:**
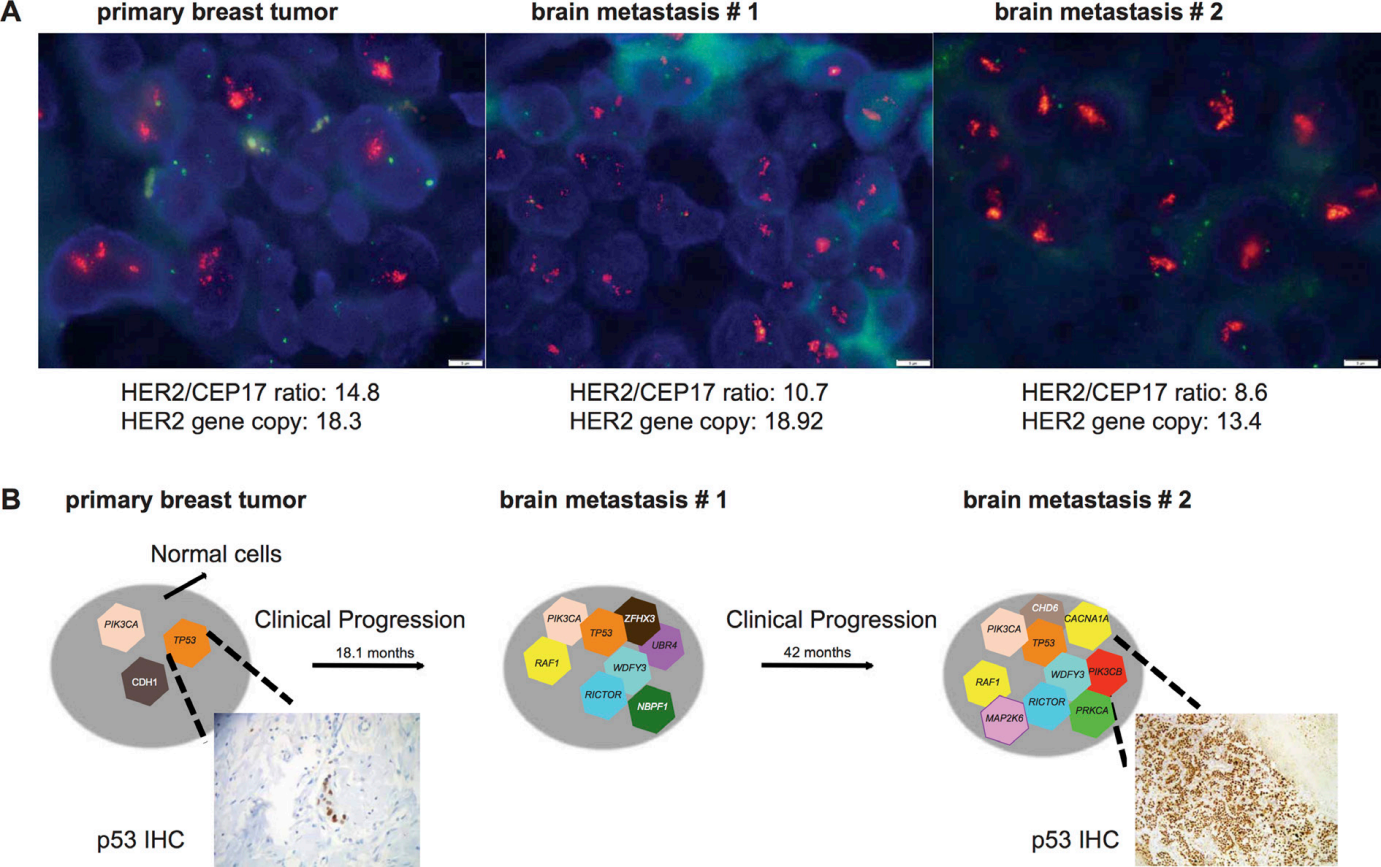
Progression and associated shift in clonal composition of Case 12 (**A**) Fluorescent *in situ* hybridization representative micrographs characterize the *HER2* status of the primary breast cancer, brain metastasis #1, brain metastasis #2 as *HER2*-amplified. HER2/CEP17 ratio and *HER2* gene copy number are shown for each sample. Scale bar: 5 microns. (**B**) Schematic shows shifts in clonal composition from the diagnosis of primary breast cancer to the development of two sequential brain metastases over a period of 60.8 months. Genes affected by likely pathogenic mutations are illustrated. P53 expression has a focal pattern in the primary tumor and is enriched in the brain metastasis (more than 80% of the cells) as depicted in the representative micrograph of immunohistochemistry with antibodies against p53 (See Materials and Methods, original magnifications 100X).

Case 12 was a 40-year-old woman, diagnosed in 2005 with locally advanced ER-positive/HER2-positive breast cancer (Figure 3A). The patient was treated with neoadjuvant therapy (anthracycline and taxane-based therapy), mastectomy and adjuvant chemotherapy, followed by adjuvant radiotherapy and trastuzumab plus tamoxifen. The patient developed a single cerebellum metastasis (brain metastasis # 1) 18.1 months from after the diagnosis of primary breast cancer. This patient had a complete surgical excision of the brain metastasis in 2007 (ER- and PR-negative, HER2 not tested during clinical care), followed by radiotherapy. The patient recurred after 17 months and was treated with radiosurgery and anti-HER2 and taxane-based therapies achieving partial response with an incomplete surgical excision in 2009. The residual lesion increased in size five months after surgery. The patient started treatment with lapatinib and capecitabine. The brain metastasis # 2 was surgically treated in 2010 after six months on chemotherapy (ER- and PR-negative, HER2-positive) (Supplementary Figure 3). After a new CNS progression, the patient was treated with anti-HER2 systemic therapy, and expired in 2011.

The analysis of the sequential brain metastases revealed that two likely pathogenic hotspot mutations *TP53* H214R and *PIK3CA* H1047R were enriched in the brain metastasis as compared to the primary tumor (Figure 2 and Figure 3B). In particular, the enrichment of the *TP53* H214R mutation coupled with LOH of the wild-type allele in the brain metastases was confirmed by p53 IHC demonstrating the evident enrichment of p53 expression in more than 80% of the cells in brain metastasis #2 compared to the primary breast cancer (Figure 3B). We identified six mutations that were present in both brain metastases but not in the primary tumor, including three likely pathogenic clonal mutations affecting *RAF1* and *WDFY3*. Additionally, private mutations were identified in each of the two brain metastases, including a likely pathogenic mutation in *ZNF703* in brain metastasis #1 and likely pathogenic mutations affecting *MAP2K6*, *PRKCA*, *CACNA1A* and *CHD6* in brain metastasis #2 (Figure 3B). These results suggest that progression from primary tumor to brain metastasis after several lines of chemo and targeted therapy was associated with clonal shifts and the acquisition of additional mutations.

### Clinical ‘actionability’ of genetic alterations present in HER2-positive brain metastases

Given that the progression of the breast cancers to the brain metastasis (median 22.75 months, range 7.5–39, Table 1) showed evidence of spatial and temporal genetic heterogeneity, we interrogated the 402-gene list derived from DGIdb [21, 22] and the 373-variant list from OncoKB [23] (Supplementary Tables 5 and 6) to determine the actionability of the likely pathogenic mutations that were private to, enriched in or associated with secondary LOH events in the brain lesions, and the genes affected by CNAs private to the brain lesions.

Among the 54 genes affected by 73 mutations, as well as *KRAS* and *CDKN2A* (affected by amplification and homozygous deletion, respectively, restricted to the brain metastases in the Index case) private to, enriched in or associated with secondary LOH events in the brain lesions, 11 genes were considered actionable based on DGIdb (Table 2). In particular, both mutations in *PIK3CA* and all four mutations in *TP53* affected hotspot residues (Table 2). Considering the actionability of specific alterations based on OncoKB, *CDKN2A* homozygous deletion (level 4 evidence), and both oncogenic mutations in *PIK3CA* were considered clinically actionable (level 3A evidence for breast cancer, Table 2). Taken together, our results suggest that a small but important subset of genetic alterations private to, enriched in or associated with secondary LOH events in the brain lesions may be clinically targetable.

**Table 2:** Potentially targetable genes and systemic therapies for genes harboring likely pathogenic mutations that were private to, enriched in or associated with secondary LOH events in the brain lesions, and the genes affected by CNAs private to the brain lesions

Actionable genes (DGIdb)	Private to the brain metastases	Hotspot mutation	Actionable alteration (OncoKB)	Selected systemic therapies
*ATR*	Index case (C2150W)^*^	-	-	Nirapanib, BMN673, Olaparib, Rucaparib, Veliparib
	Case 14 (E1878Q)^*^	-	-	
*BRAF*	Case 14 (E703D)^*^	-	-	Vemurafenib, Dabrafenib, Dabrafenib + Trametinib, Vemurafenib + Cobimetinib, Trametinib (Level 1)
*CDKN2A*	Index case (homozygous deletion)^*^	-	Yes	Palbociclib + Letrozole (Level 4)
*FGFR2*	Index case (D759H)^*^	-	-	JNJ-42756493, Debio1347 (Level 3A)
*KRAS*	Index case (amplification)^*^	-	^#^	Docetaxel + Trametinib, Abemaciclib, Erlotinib + Binimetinib, Selumetinib, Binimetinib, Ribociclib + Trametinib, Palbociclib, Ribociclib, Palbociclib + PD0325901, Trametinib Binimetinib + Alpelisib, Cobimetinib + GDC-0994 (Level 4)
*MLH1*	Case 14 (R100Q)^*^	-	-	-
*PIK3CA*	Case 12 (H1047R)^**^	Yes	Yes	Buparlisib, Serabelisib, Alpelisib + Fulvestrant, Copanlisib, Fulvestrant + Taselisib, GDC-0077, Alpelisib, Buparlisib + Fulvestrant, Taselisib (Level 3A)
	Index case (K111T)^**^	Yes	Yes	
*RAF1*	Case 12 (D19Y and D23H)^*^	-	-	Sorafenib (Level 4)
*TOP2A*	Case 14 (D524N and D526N)^***^	-	-	Doxorrubicin, Teniposide, Valrubicin, Idarubicin, etoposide
*TP53*	Case 1 (Y236C)^*^	Yes	-	-
	Case 2 (R248W)^*^	Yes	-	
	Case 12 (H214R)^**^	Yes	-	
	Case 14 (Q331^*^)^***^	Yes	-	
*ZNF703*	Case 12 (S389W)^*^	-	-	-

^*^Private to the brain metastasis

^**^Enriched in the brain metastasis

^***^Associated with LOH in the brain metastasis

^#^as per OncoKB, oncogenic mutations in *KRAS* are actionable (level 4 evidence).

## DISCUSSION

The development of brain metastases is a major limitation to life expectancy and contributes to the poor outcomes to patients with HER2-positive breast cancer. Patients with HER2-positive breast cancer have a higher risk of developing brain metastases [24]. This may be a direct result of the inability for trastuzumab to efficiently overcome the blood-brain barrier. Although traditional modes of administration of trastuzumab improve the outcome of HER2-positive breast cancer patients with brain metastases [25, 26], intrathecal administration might represent an alternative to overcome blood-brain barrier [27]. Studies comparing the genetic alterations in paired primary breast cancer and distant metastases have been performed [28–31]. However, the unique biology and challenging clinical course of HER2-positive breast cancers metastasized to the brain suggest that they warrant further and specific characterization to identify candidate molecular targets.

To define the genetic alterations associated with the development and progression of brain metastasis from patients with HER2-positive breast cancers, we assessed the somatic genetic alterations in the primary breast cancer and in up to three brain metastatic deposits. Recent studies of paired primary breast cancer and distant metastases (including but not limited to the brain) revealed that mutations in *TP53*, *PTEN*, *KRAS* and *SMAD4* were frequently restricted to the metastases [28–31]. In addition, *ESR1*, *PALB2, FSIP2*, *AGRN*, *FRAS1*, *IGFN1*, *EDC4*, *OSBPL3* were found to be significantly more frequent in prospectively accrued metastatic breast cancers than in primary breast cancers from The Cancer Genome Atlas (TCGA) [32]. These observations, however, largely stem from the analysis of ER-positive/HER2-negative metastatic breast cancers [32]. In our series of six HER2-positive breast cancers with brain metastasis, four cases harbored *TP53* likely pathogenic mutations private to or enriched in the brain metastases. Indeed, our results here, as well as those from previous studies [33–36], suggest that breast cancers may undergo clonal shifts and acquire additional somatic genetic alterations in the progression from primary to metachronous brain metastasis after systemic therapy. *HER2* amplification as a truncal alteration is likely to be true for the majority of HER2-positive breast cancers. A small subset of HER2-positive breast cancers, however, exhibit non-uniform patterns of HER2 overexpression and *HER2* gene amplification [37]. In these cases, it is uncertain whether *HER2* amplification is an early alteration that is lost during tumor development or is gained later in the evolution of the tumor. In fact, loss of HER2-positive status can occur in metastatic tumors from patients with primary HER2-positive breast cancer [38], probably due to eradication of the HER2-positive major clone and secondary expansion of a minor clone lacking *HER2* gene amplification. An important clinical implication of this observation is that sequencing of primary biopsies alone may miss a substantial number of opportunities for targeted therapy in this cohort of patients [34].

Currently, there are increasing efforts to match potential clinically actionable genetic alterations with targeted therapy to accomplish the goals of precision medicine in the context of brain metastases [34]. A recent sequencing analysis of a series of 86 pairs of primary solid tumors and matched brain metastasis, including 21 cases with breast cancer primary, demonstrated that in 53% of cases, at least one clinically actionable genetic alteration was restricted to the brain metastasis, in particular alterations that may confer sensitivity to PI3K/AKT/mTOR, CDK, HER2/EGFR and MAPK pathway inhibitors [34]. In the six patients analyzed in this study, we found alterations that may confer sensitivity to PI3K/AKT/mTOR, CDK and MAPK pathway inhibitors in two (Index case and Case 12, *PIK3CA* K111T and H1047R, respectively), one (Index Case, *CDKN2A* homozygous deletion), and two cases (Case 14, *BRAF* E703D and Case 12, *RAF1* D19Y and D23H), respectively. Additionally, we identified likely pathogenic mutations private to or enriched in the brain metastases that are potentially targetable with PARP inhibitors and/or drugs that target replication stress in two cases (likely pathogenic mutations in *ATR* in Index case and Case 14), or with multi-target angiokinase inhibitors in one case (*FGFR2* D759H in Index case). Taken together, four of six cases (67%) were found to harbor at least one likely pathogenic genetic alteration private to or enriched in the brain metastases that may be clinically targetable (Table 2). Of note, most of these genetic alterations can be targeted with small molecule inhibitors that can potentially cross the blood-brain barrier. Moreover, the majority of brain macrometastases (> 1 mm diameter) show variable extent of disturbance of the blood-brain barrier [39, 40], potentially allowing the small molecule inhibitors and other agents to reach the brain metastases. Our results suggest that different subtypes of HER2-positive breast cancer patients may be defined based on their repertoire of genetic alterations in the brain metastasis and potential targeted drugs directed against these alterations tested in the context of clinical trials.

In Case 14 (ER-negative/HER2-positive) we identified an enrichment in the signatures related to APOBEC-mediated mutagenesis (signatures 2 and 13) [20], which was previously described as enriched in metastatic tumors compared to primary tumors, in the context of HER2-negative breast cancers [32]. Our observation provides evidence to suggest that APOBEC-mediated mutagenesis may contribute to the genetic heterogeneity between the primary tumor and the brain metastasis in HER2-positive breast cancer. Our findings suggest that further studies investigating shifts in mutational signatures in the progression of breast cancers from patients receiving specific modalities of systemic therapies are warranted.

Our study has several limitations. First, due to the challenges posed in tissue procurement of brain metastasis, our cohort size was small. Our patient population comprises patients with HER2-positive breast cancers that developed brain metastasis as a unique or main metastatic site and underwent brain metastasis excision or rapid autopsy. Despite the small sample size, however, we identified potentially actionable genetic alterations private to, enriched in or associated with secondary LOH events in the brain lesions in *ATR, BRAF, FGFR2, MAP2K4, PIK3CA, RAF1* and *TP53*. It should be noted that some of the *TP53* and *PI3KCA* mutations were private mutations (that is, not found in matched primary tumors – *TP53*: cases 1 and 2; *PI3KCA: case* index). These variants are usually clonal in primary breast tumors, although they have also been reported to be subclonal in a subset of primary breast cancers [41]. In this cohort, these alterations were clonal in the brain metastasis of cases index and 1. We cannot exclude the possibility that these mutations would have been detected in the primary tumors with deeper sequencing or as a minor clone in spatially distinct areas of the primary tumor. It is unlikely, however, that these alterations would not have been detected in the primary tumor if they were present in the dominant clone of the primary tumor. In addition, caution should be exercised in the interpretation the clinical impact of these somatic genetic alterations, given that these databases are evolving. For example, *TP53* mutations are not considered therapeutically actionable by OncoKB [23] but *TP53* was considered an actionable gene by DGIdb [21, 22]. Second, we performed a targeted sequencing analysis for the 254 genes frequently mutated in breast cancer and/or involved in DNA repair. Whole-exome/genome sequencing may reveal even more potentially actionable or targetable genetic alterations restricted to or enriched in the metastatic lesions or primary breast cancers [42]. Third, in the expansion cohort, the brain metastases were not amenable to punches or biopsies in spatially separated areas of the tumors. In clinical practice, sampling brain metastases as the sole site of disease or in the context of disseminated extra-cranial breast disease is practically unfeasible [8, 43, 44]. Cell-free DNA from cerebro-spinal fluid [43] may be more appropriate in capturing the genetic heterogeneity and private mutations within the brain metastases, but unlike the current study, we would not have been able to attribute the mutations to the brain metastasis.

Despite these limitations, this study provides evidence that genetic alterations that are private to or enriched in the brain metastases of HER2-positive breast cancers may represent potential targets for pharmacological inhibition. We would contend that studies of larger patient cohorts and of larger collections of genes are warranted to expand the characterization of potentially targetable genetic alterations in HER2-positive breast cancer patients with brain metastasis.

## MATERIALS AND METHODS

### Patients and samples

An index HER2-positive breast cancer patient with multiple anatomically distinct brain metastatic deposits and complete clinical remission of the systemic disease at the time of death was subjected to a rapid autopsy. Five additional HER2-positive breast cancer patients, who had minimal or absent extra-cranial disease, were subjected to brain metastases excision were also included (Table 1, Supplementary Methods). Tumor and matched normal tissue were subjected to microdissection and DNA extraction as previously reported [43].

### Immunohistochemistry and fluorescence *in situ* hybridization

HER2 status was assessed in all primary breast tumor tissues by either immunohistochemistry (IHC) and/or fluorescence *in situ* hybridization (FISH) according to the American Society of Clinical Oncologists (ASCO)/College of American Pathologists (CAP) guidelines [45] (Supplementary Methods). p53 IHC was performed using the clone DO7 antibody (Cell Marque, Rocklin, CA). Tumors were classified as p53-positive if > 10% of morphologically unequivocal neoplastic cells displayed strong nuclear expression as previously described [46].

### Targeted capture massively parallel sequencing

Tumor and matched normal DNA samples were subjected to targeted sequencing using a previously described customized panel targeting all exons of 254 genes recurrently mutated in breast cancer and/or related to DNA repair (Supplementary Table 1 and Supplementary Methods) [47, 48]. Bioinformatics analyses were performed as previously described [46–51]. Allele-specific copy number alterations (CNAs) and loss of heterozygosity (LOH) of the wild-type allele in genes harboring a somatic mutation were inferred using FACETS [19]. Sequence reads have been deposited to the NCBI Sequence Read Archive (SRP070781).

### Validation of mutations by amplicon sequencing

Selected mutations found by targeted sequencing (*n* = 108, consisting of 104 unique mutations) were subjected to orthogonal validation using amplicon resequencing in all samples for a given patient, where sufficient genomic DNA was available (Supplementary Table 3 and Supplementary Methods). The validation rate of the somatic mutations with sufficient coverage was 93% (100/108).

### Identification of mutations private to or enriched in the brain metastatic lesion

ABSOLUTE (v1.0.6) [11, 52] was used to infer the cancer cell fraction (CCF) of mutations and the mutations were classified as clonal or subclonal as previously described [46–48, 53] (Supplementary Methods). Mutations were defined as ‘private to the metastatic lesion’ if they were absent in the primary tumor but present in the brain metastasis. Mutations were defined as ‘enriched in the brain metastasis’ if their CCFs increased by at least 20% in the brain metastases compared to the respective primary tumors. Mutations associated with LOH of the wild-type allele in the metastasis but not in the corresponding primary tumor were defined as ‘LOH in metastasis’.

### Identification of potentially pathogenic and/or actionable somatic genetic alterations

MutationTaster, CHASM (breast) and FATHMM [54–56] were used to define the potential functional effect of missense single nucleotide variants as previously described [46–48, 53, 57] (Supplementary Methods). Frameshift, splice-site and nonsense mutations were considered likely pathogenic if they were targeted by loss of the wild-type allele or affected haploinsufficient genes [58]. The Drug-Gene Interaction database (DGIdb) [21, 22] and the OncoKB platform [23] were used to investigate the clinical actionability of mutated genes (Supplementary Methods).

### Phylogenetic tree construction

A maximum parsimony tree was built for the index case using binary presence/absence matrix based on the somatic non-synonymous and synonymous mutations, gene amplifications and homozygous deletions in the biopsies of the primary tumor and the metastatic lesions, as previously described [59, 60] and Supplementary Methods.

### Mutational signatures

Decomposition of the mutational signature was performed using deconstructSigs [61], based on the set of 30 mutational signatures (“signature.cosmic”, based on the signatures at http://cancer.sanger.ac.uk/cosmic/signatures [15, 20]), for the case with at least 20 somatic mutations.

### Statistical analysis

All statistical analyses were performed in R v3.1.2. Comparisons of continuous variables were performed using Mann-Whitney *U* tests. Association was performed using the Spearman rank-correlation test. All statistical tests were two-tailed and *P* < 0.05 was considered statistically significant.

## SUPPLEMENTARY MATERIALS FIGURES AND TABLES










