# Supplementary material for: Genetic heterogeneity and actionable mutations in HER2-positive primary breast cancers and their brain metastases
Source: Oncotarget. 2018 Apr 17;9(29):20617–30. doi: 10.18632/oncotarget.25041 (PMC5945519; doi:10.18632/oncotarget.25041)
Supplement: Supplementary file 1 [file oncotarget-09-20617-s001.pdf]

# Genetic heterogeneity and actionable mutations in HER2-positive primary breast cancers and their brain metastases

## SUPPLEMENTARY MATERIALS

## SUPPLEMENTARY METHODS

### Patients and samples

An index HER2-positive breast cancer patient subjected to warm autopsy and five additional HER2-positive breast cancer patients were included (Table 1). All patients received anti-HER2 targeted therapy as part of their systemic treatment. The tumor materials of the index patient were obtained in the autopsy program of the Vall d'Hebron University Hospital (VHUH). Formalin-fixed, paraffin-embedded (FFPE) samples of the remaining patients were retrieved from the archives of the Pathology Department of VHUH and Dexeus University Hospital, Barcelona, Spain. The study was approved by the Institutional Review Boards of both hospitals. Histologic sections were cut from representative frozen tumor tissue of the index patient and diagnostic FFPE blocks. Tumor and matched normal tissue were subjected to microdissection and DNA extraction as previously reported [1].

### HER2 immunohistochemistry and fluorescence *in situ* hybridization

Selected primary breast cancers and brain metastases with available tumor tissue were subjected to re-assessment of HER2 status using IHC (anti-HER-2/neu (4B5) Rabbit Monoclonal Primary Antibody kit, Ventana Medical Systems) and/or FISH. FISH for *HER2* was performed on 4 µm-thick sections using the Food and Drug Administration (FDA)-approved Vysis PathVysion HER-2 DNA Probe Kit (Abbott Molecular Inc, Des Plaines IL, USA) according to manufacturer's instructions. Normal tissues including vessels, fibroblasts, lymphocytes or non-tumor breast tissues served as internal controls. Tumor tissues were evaluated using a 100× objective.

### Targeted capture massively parallel sequencing

Tumor and normal DNA samples were subjected to targeted capture massively parallel sequencing at the Memorial Sloan Kettering Cancer Center (MSKCC) Integrated Genomics Operation (IGO). In this study, we employed a previously described customized breast cancer panel targeting all exons of 254 genes recurrently mutated in breast cancer and DNA repair-related genes (Supplementary Table 1) using custom oligonucleotides (NimblegenSeqCap) as described [2, 3]. Barcoded sequence libraries were prepared (New England Biolabs, KapaBiosystems) using 50 ng–250 ng of DNA. Massive parallel sequencing was performed on an Illumina HiSeq2000 (San Diego, CA), and bioinformatics analyses were performed as previously described [2, 3]. Allele-specific copy number alterations (CNAs) and loss of heterozygosity (LOH) of the wild-type allele in genes harboring a somatic mutation were inferred from massively parallel sequencing data using FACETS [4].

### Validation of mutations by amplicon sequencing

Selected mutations found by targeted sequencing ($n$ = 108, consisting of 104 unique mutations) were subjected to orthogonal validation using amplicon resequencing in all samples for a given patient, where genomic DNA was available in the MSKCC IGO (Supplementary Table 2). For amplicon resequencing, 5 ng of genomic DNA was amplified with primers specific for a given mutation using the AmpliTaq gold 360 master mix (Invitrogen Life Science Technologies). Amplicons were bead-purified, quantified and pooled. Pooled amplicons were subjected to standard protocol of Illumina library preparation sequencing (MiSeq). Reads were aligned to the reference human genome GRCh37 using BWA (v0.6.2) [5], and local realignment was performed using GATK (v3.1.1) [6]. Pileup files were generated using SAMtools [7]. Mutations with > 1% of mutant allele frequency (MAF) and covered by at least 50 reads were considered validated. The validation rate of the somatic mutations with sufficient coverage was 93% (100/108). False-positive variants were excluded from further analyses. Given the high validation rate, untested mutations were included in further analyses. In addition, of the 70 mutations that were not detected in a sample of a given case based on the initial targeted sequencing, 11 (16%) were subsequently detected by amplicon

sequencing and were included in the analyses, with the remaining confirmed to be genuinely absent even at a median depth of 10564× (range 272×–49532×).

## Inference of cancer cell fraction (CCF)

ABSOLUTE (v1.0.6) [8] was used to infer the cancer cell fraction (CCF) of each mutation using the number of reads supporting the reference and the alternate alleles and the segmented $Log_2$ ratio from targeted capture massively parallel sequencing. A mutation was classified as clonal if its probability of being clonal was > 50% [9] or if the lower bound of the 95% confidence interval of its CCF was > 90%. Mutations that did not meet the above criteria were considered subclonal.

## Identification of potentially pathogenic mutations

A combination of MutationTaster [10], CHASM (breast) [11] and FATHMM [12] was used to define the potential functional effect of each missense somatic single nucleotide variant (SNV). Missense SNVs defined as non-deleterious/passenger by both MutationTaster [10] and CHASM (breast) [11], a combination of mutation function predictors shown to have a high negative predictive value [13], were considered likely passenger alterations. The remaining missense SNVs were defined as likely pathogenic if they were predicted to be "driver" and/ or "cancer" by CHASM (breast classifier) and/ or FATHMM [12], respectively. Frameshift, splice-site and nonsense mutations were considered likely pathogenic if they were targeted by loss of the wild-type allele or affected haploinsufficient genes [14]. SNVs, including missense and nonsense SNVs, affecting hotspot residues [15] were also considered likely pathogenic and were separately annotated. Mutations were also annotated if they affected genes included in the cancer gene lists described by Kandoth et al. (127 significantly mutated genes) [16], the Cancer Gene Census [17] or Lawrence et al. (Cancer5000-S gene set) [18]. Mutations that were neither likely pathogenic nor likely passenger were considered of indeterminate pathogenicity.

## Actionable somatic genetic alterations

The DGIdb database [19] defines a list of 402 known and potential actionable genes (Supplementary Table 5). The clinically actionability of DGIdb is defined based on Bader Lab Genes, Caris Molecular Intelligence, Foundation One Genes, GO, Guide To Pharmacology Genes, Hopkins Groom, MSK-Impact, Russ Lampel and dGene.

OncoKB [20] is a comprehensive and curated precision oncology database with more than 3,000 unique mutations, fusions, and copy number alterations in 418 cancer-associated genes. It annotates the biologic and oncogenic effects, and provides the level of evidence that a specific molecular alteration is predictive of drug response on the basis of US Food and Drug Administration labeling, National Comprehensive Cancer Network guidelines, disease-focused expert group recommendations, and scientific literature.

## Phylogenetic tree construction

A maximum parsimony tree was built for each case using binary presence/absence matrices based on the repertoire of somatic non-synonymous and synonymous somatic mutations, gene amplifications and homozygous deletions in the biopsies of the primary tumor and the metastatic lesion, as described by Murugaesu *et al.* [21]. A starting tree was constructed using the Neighbor-joining method and Hamming distance and optimized using the parsimony ratchet method implemented in the R package Phangorn [22]. Trees were rooted at the hypothetical normal where all somatic alterations are absent. Branch lengths were determined according to the ACCTRAN criterion as implemented in the Phangorn package and were drawn to scale.

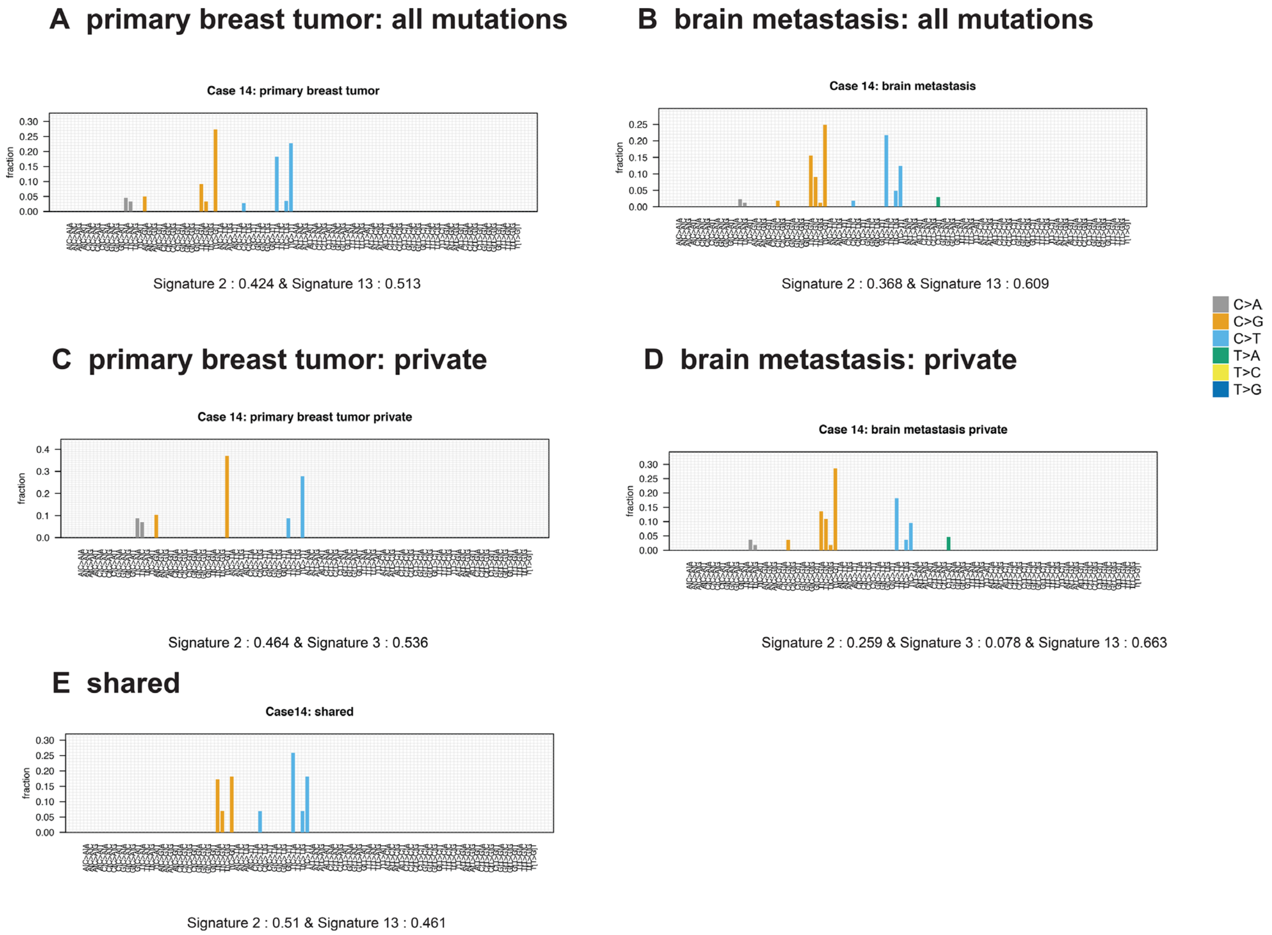


**Supplementary Figure 1:** The mutational signatures of Case 14 were derived from the analyses of the somatic mutations in the primary breast cancer (**A**), mutations in the metastasis (**B**), mutations private to the primary (**C**), mutations private to the metastasis (**D**) and mutations shared between the primary and the metastasis (**E**). Below the graphs is the inferred contribution of the different signatures as defined by deconstructSigs.

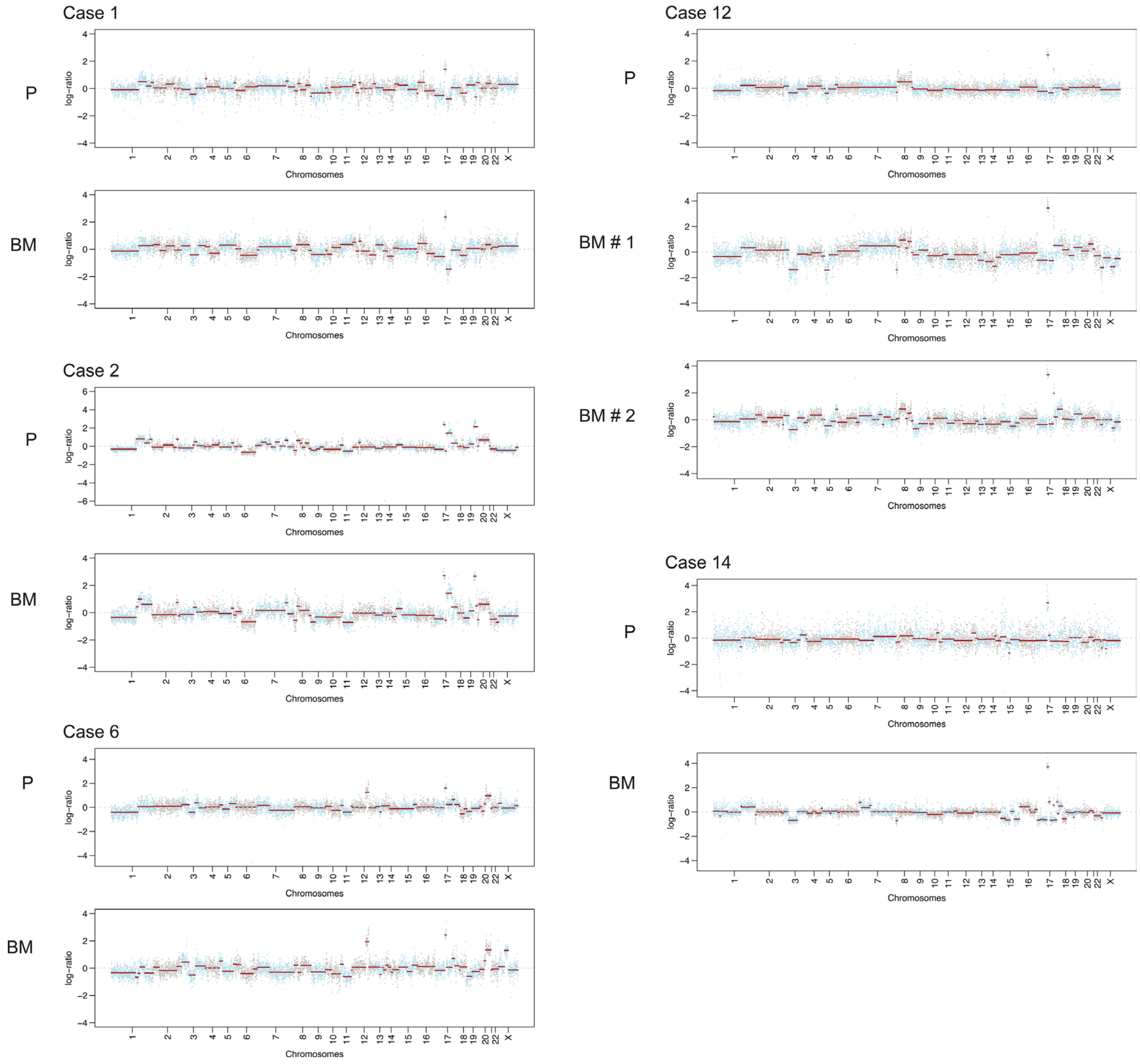


**Supplementary Figure 2: Genome plots of the primary tumor and matched brain metastatic lesions of the expanded cohort.** Smoothed $Log_2$ ratios were plotted on the y-axis according to their genomic positions indicated on the x-axis. P, primary breast tumor; BM, brain metastasis.

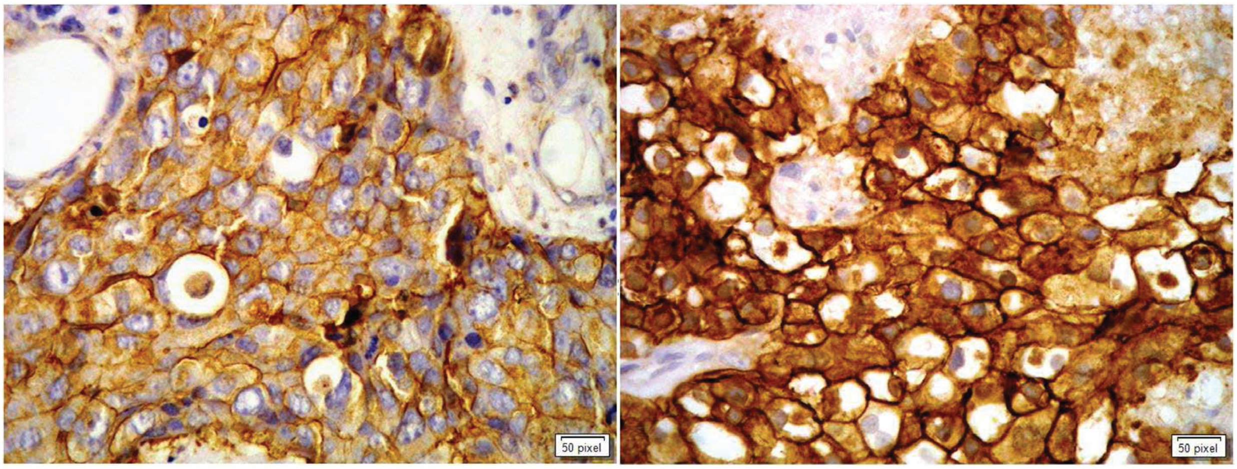


**Supplementary Figure 3: HER2 immunohistochemistry analysis of the brain metastasis # 1 and brain metastasis # 2 of case 12 revealed 2+ and 3+ respectively.** *HER2* FISH was performed (Figure 3) and confirmed the HER2-positive status and the clonality of *HER2* gene amplification in both metastases.

## Supplementary Table 1: List of 254 genes included in the targeted capture massively parallel sequencing platform. See_Supplementary_Table 1

## Supplementary Table 2: Targeted capture massively parallel sequencing metrics

| CASE | SAMPLE TYPE | TOTAL READS | MEAN TARGET COVERAGE | PCT TARGET BASES 2X | PCT TARGET BASES 10X | PCT TARGET BASES 20X | PCT TARGET BASES 30X | PCT TARGET BASES 40X | PCT TARGET BASES 50X | PCT TARGET BASES 100X |
|---|---|---|---|---|---|---|---|---|---|---|
| | Brain metastasis # 1 | 48048307 | 1267,213 | 0,995735 | 0,994316 | 0,993246 | 0,992441 | 0,991607 | 0,990941 | 0,988323 |
| | Brain metastasis # 2 | 50599385 | 1503,425 | 0,995677 | 0,994243 | 0,992957 | 0,992073 | 0,991239 | 0,990539 | 0,988221 |
| Index | Brain metastasis # 3 | 45203969 | 1055,731 | 0,995394 | 0,993717 | 0,992747 | 0,992014 | 0,991103 | 0,990384 | 0,987495 |
| | Normal | 42361375 | 1108,845 | 0,995955 | 0,994126 | 0,993039 | 0,992554 | 0,992276 | 0,991878 | 0,990373 |
| | Primary breast | 34116949 | 238,3156 | 0,993074 | 0,990684 | 0,988394 | 0,985795 | 0,983055 | 0,979023 | 0,904022 |
| | Brain metastasis | 57406724 | 763,2532 | 0,995326 | 0,994141 | 0,993744 | 0,993204 | 0,992741 | 0,992323 | 0,990462 |
| 1 | Normal | 58003315 | 467,4232 | 0,994433 | 0,992679 | 0,99166 | 0,991177 | 0,990648 | 0,990121 | 0,987549 |
| | Primary breast | 61824743 | 645,3389 | 0,994925 | 0,993728 | 0,992702 | 0,992121 | 0,991615 | 0,991126 | 0,988996 |
| | Brain metastasis | 42659079 | 415,2256 | 0,994081 | 0,992218 | 0,991226 | 0,990537 | 0,990026 | 0,989538 | 0,986106 |
| 2 | Normal | 51107027 | 786,7607 | 0,994301 | 0,992785 | 0,991669 | 0,991119 | 0,990707 | 0,990347 | 0,988573 |
| | Primary breast | 44873472 | 511,0891 | 0,994403 | 0,992169 | 0,991239 | 0,990588 | 0,99018 | 0,989803 | 0,987792 |
| | Brain metastasis | 34393591 | 155,1913 | 0,993217 | 0,990699 | 0,989472 | 0,987952 | 0,984919 | 0,976402 | 0,68798 |
| 6 | Normal | 36155954 | 196,0684 | 0,993696 | 0,991216 | 0,990123 | 0,989217 | 0,988143 | 0,9868 | 0,925636 |
| | Primary breast | 38695731 | 378,4272 | 0,993568 | 0,991259 | 0,990466 | 0,989925 | 0,989255 | 0,988534 | 0,985533 |
| | Brain metastasis # 1 | 43375387 | 369,1571 | 0,993638 | 0,991245 | 0,989969 | 0,988864 | 0,987559 | 0,984818 | 0,927521 |
| 12 | Brain metastasis # 2 | 40464724 | 139,4645 | 0,993803 | 0,99082 | 0,988889 | 0,987264 | 0,985473 | 0,981565 | 0,728588 |
| | Normal | 44537893 | 421,6744 | 0,994169 | 0,991955 | 0,99069 | 0,989903 | 0,989129 | 0,988579 | 0,984146 |
| | Primary breast | 38819977 | 83,78619 | 0,994149 | 0,990627 | 0,988843 | 0,985459 | 0,974464 | 0,929262 | 0,204791 |
| | Brain metastasis | 46309989 | 1148,308 | 0,995703 | 0,994259 | 0,993571 | 0,993001 | 0,992515 | 0,992201 | 0,990981 |
| 14 | Normal | 63703135 | 1789,751 | 0,996294 | 0,994981 | 0,994219 | 0,99391 | 0,99346 | 0,993234 | 0,992445 |
| | Primary breast | 39134184 | 446,5772 | 0,994173 | 0,992124 | 0,991113 | 0,990454 | 0,989827 | 0,989226 | 0,985634 |

**Supplementary Table 3: List of primers used for the validation of mutations using amplicon resequencing (MiSeq)**

| Gene and mutation | Primer Forward | Primer Reverse |
|---|---|---|
| ABCA13_S771R | GGAATTTTTTGAGAAATTATTGTTGC | ATTTTCCAAATCACTTCGTTGC |
| AHNAK2_E201Q | AGGGTAGTGAAGCCAGCTGG | AAACATAAAATATGAAGATGCTCTCAA |
| AHNAK2_L4986R | AGGCTTTGTGCTCCTCCC | TTGAAAATGCCTAAGATTAAGCTTC |
| AK9_K1173N | AAACAGATGCTGAACAGATTTATTCA | GATTTTTCCCAGATGCAGCT |
| AKAP9_E102* | GATTCCTGAATCTACAATAATGAGAAC | GACATAAAAATCATGATCAAATTGC |
| AKAP9_Q1130E | GCTACAGATGGAAGCCCAAC | TGAAGTTCCTTGATCTTTTCCTC |
| AKT1_K377R | CGCCTGGCGCAGGGGCAG | TGGTCATGTACGAGATGATGTGCGG |
| ANK3_E3429Q | CTTTGGCTAAAAGAGCGGTC | TTCCATTGCCACCACAGC |
| AOAH_R630T | TTGAGAGAAAGACATTTGCAAAGA | AGGAGAATCCGTTCAACCC |
| APOBEC3A_L55V | CTTTAACAATGGCATTGGAAGG | AAACCACAAGTACAATCCGGA |
| APOBEC4_S25F | TGTCCCATAAGGGAATCCAA | TGCAGCTAGAAGACAGTAAATCTAGC |
| ARID1A_S138L | GGGAACGCGGGCCCTA | TTGGTGGAAGACGGCGG |
| ATM_E2744 | TTCATGCTTAATTATTCTGAAGGG | CACCCAACCAAATGGCAT |
| ATR_C2150W | TTGACACAGCTGTCATCATCC | ATGATTTGGGTAAAATAAACAAGGTT |
| ATR_E1878Q | GTAGTGTGAGAAACCTTTTGTTGAG | AAGGCTGAATATGATAACATTTTAACA |
| ATRX_P711H | TCTGAAGAAGAACTGTGACTCATCC | ACCCTGTAACATCTAATTCAGATGAAG |
| BRAF_E703D | CACTGTGTGCCCAAAAAAGT | GACGAGGATACCTGTCTCCAGA |
| BRAF_L232R | AGTTCCAAAATTACTCATCCATATTTC | ATAACCAAGAAAGGCTTGTGTCTAC |
| BRCA1_S449C | AATTAGATTTTCAGTTACATGGCTTAA | CTAAATGAGGTAGATGAATATTCTGGT |
| BRCA2_E3343K | AACACCCATAAAGAAAAAAGAACTGA | GAACTGGTGGGAGCAGTCC |
| BRCA2_L997V | AAAAATGACTCTAGGTCAAGATTTAAA | AACATTTTGCTCTTCTTAATGTTATGT |
| CACNA1A_G682R | CCAGGCCTGTCCCCTTCT | GTGGCTGAGAGGGGGGTT |
| CACNA1A_K1365T | ATGTGTTGTCCTTGAGCAGTGT | CTCCACAGTGGCAATAGCAA |
| CACNA1A_L1377R | AGTGCAGTGGAAGAATTTCCC | GATGGGCCACTGGAGG |
| CACNA1C_A317V | TTCTTTCTCTGCCCACATCTC | GGCAAAGTTGTCAAAGTTGGT |
| CACNA1C_E1948K | TGCTTTCCGGGGGTG | GCGGTGCATTTACCTAGTGAG |
| CACNA1E_L1575V | TGAGGGATAAATACACAAATACTAGTG | TCAGAGAGGATTTGCTGGTACC |
| CACNA1E_L2058V | TCGGGATAAGCGTTCAAATT | GTAAGTGCATCAGGGCAGG |
| CDH1_Q23 | GGAGGGGCGGCGCTGTT | TCACCTCTGCCCAGGACGC |
| CDH1_Q346 | TGTGTTGGGCTGGGCTAG | GTGGTGGGATTGAAGATCG |
| CEP164_S1123C | TCCTGCTCCAGCCTGTCC | CTTGATGCCTGGTGGGTC |
| CHD4_D314Y | CGGAGTTTCTTGCGGCT | GTCCCCAGTGAGAGAGTACTCC |
| CHD4_E138D | AAAGGGCAGTAAGGTGTCTAGGA | GTGAGGGCAGCGACTATACTC |
| CHD6_K2587T | TCCAGGAGATACTCCTGGGAT | CTATCAAGCACAACGAAAAGTGG |
| CHD6_L1643H | ATCAGGCAGGCTGAGATGAT | GATGCCTATAGAAACTATGCCCAG |
| CHEK2_K298T | TTCCTGAGTTTAAAAATCATCAATCT | AAAACATTCAGACAATCACTATCTTTG |
| CUBN_E1520K | GTGAAGTTCAAGAGAACACGATG | CCAGGTAATGGATTATTTTCGAG |
| DEPTOR_P325L | TGGATAGAGGACTTGGAAGTGC | ATAAGCTACCCATTGACACATGG |
| DOCK11_S1189F | TTTCAGAACCAACAAGCCAA | CATATAGCATATGACCCCAATTCC |
| EPPK1_-1464 | AGGGCCGCAGCCCTC | GTTGCCACTGCCCTCAGA |
| EPPK1_M2050I | GGAACAGCAGCCAGCC | TCGACCCACAGCACCAC |
| EPPK1_T568P | GAGGCCAGGCTGCCC | GACCCTCTCCGTGGAGAAG |
| ERBB2_D769Y | CCAGGGCATCTGGATCC | CATATCTCCCCAAACCCCA |
| ERBB2_P1227S | CCCCGAGTACTTGACACCC | GGCCTTCTGGTTCACACTG |
| ERBB3_M1114I | CAGTCTCTCTACACCCAATGCC | GGGAGAGTGGGGTAACAGG |
| ERBB3_V1101I | GGAGTCTGCAGTTTCTGGGA | GAATGGTAGGCGCTATCTCC |
| ERCC2_R196Q | AGCAAGCAACAGACAGACATG | GCCCATGGGCGTGAG |
| FANCD2_E14Q | TAACTTCTGTTTCCCGATTTTGC | AAGCAGGAACTTTATATCTCTCACATG |
| FANCD2_Q802H | AACTCAAAGATCTTGCTTTTCATAGAC | TTCATCTCAGGTGATGTTTCCT |
| FANCE_L369R | CTCTCAGACCTCGGTCTCCT | TGGGAATCCCCTCTCTCAA |
| FGFR2_D759H | GTGTGTGTAAAACACTACGCATGTC | TTCAAGGAAATCCATTTTTCCC |
| GATA3_P426P? | TCCCCAAGAACAGCTCGTT | ATGCAAGTCGAAAGGGACTG |
| HECW1_D448A | CCAGCTGAGGAAGCAGCA | GCTGGCTGGGGCTTCA |
| HIST1H3B_D78H | CAAAGGTTTGTGTCCTCAAAGAG | GGGCACTGTGGCTCTGC |
| HIST1H3B_E134Q | AAATTATCACAGCTACTTTCGTTGG | CTTGGTAGGGCTCTTTGAGGA |
| HRNR_P573L | ATGACCCGAGCTAGATCCG | TCTCCTAGCCCTAGCCGTG |
| INPP4B_Q498H | TTGAGAGGCAAGCGATATACAA | TTGTCAGGAGTGCTCTTTTAGC |
| KMT2C_D4530H | GGAAGCAGCTGACCAATTGT | TAAGGACAAAACTATGCTTTGCC |
| KMT2C_K2769N | TTTTGTTTTCTTGTTCCTTTTTTTT | ATAATTTGGAAACTAATGATCCCAAC |

| | | |
|---|---|---|
| KMT2C_R41S | GACAGCTCCGGCCCG | GAGCGTGGAGCAGCCG |
| KMT2D_E1861K | ACCCTTATACACAAAGAGGTACGG | GTGGCCATGACCCATCCT |
| LAMA5_A907T | CCTGAGGCCTGGGCCTTCAA | TACCTCCCGGACCTGCACCA |
| MACF1_G3580D | GGGATCAGAGACTCCTCATTCC | ATAAGTAGACTGGAACTTGGTGGC |
| MACF1_L617V | TCCTACCAAGCCAGCAGTAAA | GGATTCTGAAGGCTACGCAC |
| MAP2K4_S303R | CATTTGCCTATTCCTTGAGTGTAA | ATTATAGTTCTTAACGAACGGGGA |
| MAP2K6_L229H | CCATCCCCAGAGACTATTGAG | TCATTACCCACAACCTCATAGTTC |
| MDN1_-585 | AGCAAAAAATCCTAGCCTCCTT | GTTTCTCCCTCTTGTGGCC |
| MED12_E1740Q | AACAGTCCGAGTGGACCG | CCCGGTTTGTCAGTTTTGG |
| MED12_P561S | AAAAGTCAGTTCTACAATTTGTTCTGT | TAGGCAGAGGTAACTAGAGAGTGGTAG |
| MLH1_R100Q | TCTGGATATTGTATGTGAAAGGTTC | CAAGTAAAAGGTACCAAACCTTATTT |
| NBEAL2_T1422A | TCCTTCGCCACTGGATG | TGTACCAGAAGTGTCCTCCCTT |
| NBN_E510A | TTCTGCAGCATGAGATTTACTGG | TCATGCAAATCAGCAAGAATAGA |
| NBPF1_E168K | ACCACCCATTACTTGCTCCC | ACATAGATGTTCATGTCTCTGTGCA |
| NBPF1_Q974 | GGAACTTCCATAGGGCAGG | TAGCCATGAAATGTAGCTGGG |
| NCOA3_Q1358 | CTTTGGTCGAGTGTCTAGTCCTC | TGGACTTCTGGGACTAGGATGT |
| NEB_R7478Q | GCATGCCTTGGCAATG | AAAAAAAGCCTCTCTCTTGAGTAAAC |
| PARP1_D217H | TGTCGAAAGGAGACACAGAGC | CACCGCAAGTCATTGGGT |
| PAXIP1_L537F | GGTGGAACCTGATGCTGC | GCTGCAGCAGCACCAG |
| PIK3CA_E542K | TTATTTTATTTTACAGAGTAACA | TTCAGTTATTTTTTCTGTAATAAA |
| PIK3CA_H1047R | CAATCTTTTGATGACATTGCATACA | CCAGAGTGAGCTTTCATTTTCTC |
| PIK3CA_K111T | GAAAGGGAAGAATTTTTTGATGAA | TTAGACAGAAATATTTAGAAAGGGAC |
| PIK3CB_S769 | AGGCTGACAGCTGGCC | GCCAAAGGGAAGGAGGC |
| PIK3R1_D529H | AAACCATAGTGAAACTTTTCATAAACT | CGTTTGTCAATTTCTCGATACTCA |
| POLD1_E795Q | CCGATTCGGCGTGTC | CTTCACACTGTGGCTTTTGTG |
| POLH_R105G | CAGACAATCTCAAGGTTGCCT | GAGATAGGCTGACCTTGTAGCTTT |
| PRKCA_K91T | GCTCGTATGTTAAATCATGTCTATTCT | TCCCATTAACAGTAAGTGCCAA |
| PRKD1_D893N | AGTTCCACAGTGTTTTGACAGATT | CATCACCCATGAAAGTGATGAC |
| RAF1_D19Y | GAAGGATCTGTGAGTTTGCCA | TTATTTGTGTTTCTTACCAGGTTTAAG |
| RAF1_D23H | TTGCTTGTCTTAGAAGGATCTGTG | CTTACCAGGTTTAAGAATTGTTTAAGC |
| RELN_E1824Q | TAGTAGTAATGTTGGGAAGGAGAATCT | TGGACCCTATTGTGTTCCTGT |
| RELN_E701G | GACTTCAGCACCACGGATAGA | CAAAGTAGATTCGAGAATCTCCG |
| RELN_L1854R | GCACAGGGGAGAATTCTCTTG | TATAAAATCAAAGGGATACAATTTTGC |
| RELN_N290T | AGGCATATAGGAGAGTAGAAC | TTTTAACCTTGAAATTTATTGTTA |
| RICTOR_D861Y | AGAAGCAGAATATACAAAAACTCAGAA | TCCTCTAGGAATACAACTCCAAA |
| RPGR_E709K | TCCTCTTCTTCTCCTTCTCCATG | GAAGGGGGAGAAAGACAAGG |
| RPTOR_E163Q | AGTCAAGAAGCTCTGCACGTC | GATGGGCAGGACCTGCTC |
| SOS1_D169H | CTGGAGATATTCCCCAACACA | TGCAGACATTTTAAAGCTGGTT |
| SOS1_R1041C | TCTGTTTCACTTTCAGGGATCC | GAAAAACTTTCCTTTCTACTACAGTGT |
| SRCAP_E2638K | TTCTGCACCCAGCCTGAC | TCAGCCTCCAGTGGCTCC |
| SRCAP_H2925Q | CTGACCCAGTCCCTGGGC | CCCCTGCAGAGGAAATGGTC |
| STAT1_L312F | TAAACCCTTGTAAATCATCTGAATTAA | AAAGTTGGAGGAATTGGAACAG |
| TENM1_D477H | TAAAACGCCAGATACCAAGGTC | AAAAAGTAGTGGCTCATCTTCATGT |
| TENM1_S1855L | AATTTTCCCATCAGCCCAA | CGAAAATTCACCCTTCGAATT |
| TOP2A_D524N | AGACATGCTTAATTTAGTTGAACAATC | TTTTTGGTATATGTCCATTTCAGA |
| TOP2A_D526N | GACATGCTTAATTTAGTTGAACAATCT | TTTTTTTGGTATATGTCCATTTCAGA |
| TOP2A_E1226K | CACCTTGGCCCCCAA | CGATGCTTTTTTTGGTATATGTCC |
| TOP2A_V983G | AATTGAAACGTGTACATCTCACAAAA | TGTTGAATGGCACCGAGAAG |
| TP53_H214R | GCTCACCTGGAGGGCC | CCTCACTGATTGCTCTTAGGTCT |
| TP53_Q331 | AATGCCCCAATTGCAGGTA | TTTATCACCTTTCCTTGCCTCTT |
| TP53_R248W | GAGGCAAGCAGAGGCTG | GGCCTCATCTTGGGCC |
| TP53_Y236C | TGCAGGGTGGCAAGTG | TCCCCTGCTTGCCACA |
| TYK2_E437K | ACCCTTGGCGGTGGC | GGAGCTGAGCTTGCCTTC |
| TYK2_S134L | CCACAAATAGTCTTGCCACCC | GTCCCTCCTTCCTGCAGG |
| UBR4_E1114 | TCAGTCTCAGCAGCCATCTTAG | TTTCTACCATAGCCCATTGCC |
| UBR4_K1150T | GCATCTCTGGGCAGGC | AGTCTGCAGTCCATCTACACCC |
| WDFY3_K2603N | TTCAGAATAAAGAAACATGATAAAACA | TGGAACAGTGAATTTATCCCTTG |
| WDFY3_Q2808H | AGATAATGATGTTTTAAAAGGTGACAT | TTTCTTAAACAGGAGAAACTCCTGC |
| ZFHX3_L357R | GAGAGCTTCCTCCCCTTCC | AAGAACATCTCCGCTATCATCC |
| ZFHX3_Q2952E | AGTCCAATGTCATTGCCCA | AAGCCTTGCAGATCCCTG |
| ZFHX4_L686P | TACAAATATCAGCAGACCCTGG | CTGCATATGAATACTGAGGTTGC |
| ZFP36L1_D304N | AGCCCCATGCTAGGGG | CTTTAGCTTTGCTGGGTTTCC |
| ZNF703_S389W | AAGCCCCCCAGCTCCA | CGGGTGCCCGGGGTA |

**Supplementary Table 4: Somatic single nucleotide variants (SNVs) and insertion/deletions (indels) present in the samples analyzed.** See_Supplementary_Table 4

**Supplementary Table 5: List of potential clinical actionable genes according to the http://dgidb.genome.wustl.edu.** See_Supplementary_Table 5

**Supplementary Table 6: List of potential clinical actionable genes according to the http://oncokb.org/api/v1/utils/allActionableVariants.txt.** See_Supplementary_Table 6

## SUPPLEMENTARY REFERENCES


1. De Mattos-Arruda L, Mayor R, Ng CK, Weigelt B, Martinez-Ricarte F, Torrejon D, Oliveira M, Arias A, Raventos C, Tang J, Guerini-Rocco E, Martinez-Saez E, Lois S, et al. Cerebrospinal fluid-derived circulating tumour DNA better represents the genomic alterations of brain tumours than plasma. Nat Commun. 2015; 6:8839.
2. Piscuoglio S, Ng CK, Murray M, Burke KA, Edelweiss M, Geyer FC, Macedo GS, Inagaki A, Papanastasiou AD, Martelotto LG, Marchio C, Lim RS, Ioris RA, et al. Massively parallel sequencing of phyllodes tumours of the breast reveals actionable mutations, and TERT promoter hotspot mutations and TERT gene amplification as likely drivers of progression. J Pathol. 2016; 238:508–518.
3. Piscuoglio S, Geyer FC, Burke KA, Murray MP, Ng CK, Mota A, Marchio C, Berman SH, Norton L, Brogi E, Weigelt B, Reis-Filho JS. Massively parallel sequencing analysis of synchronous fibroepithelial lesions supports the concept of progression from fibroadenoma to phyllodes tumor. NPJ Breast Cancer. 2016; 2:16035.
4. Shen R, Seshan VE. FACETS: allele-specific copy number and clonal heterogeneity analysis tool for high-throughput DNA sequencing. Nucleic Acids Res. 2016; 44: e131.
5. Li H, Durbin R. Fast and accurate short read alignment with Burrows-Wheeler transform. Bioinformatics. 2009; 25:1754–1760.
6. McKenna A, Hanna M, Banks E, Sivachenko A, Cibulskis K, Kernytsky A, Garimella K, Altshuler D, Gabriel S, Daly M, DePristo MA. The Genome Analysis Toolkit: a MapReduce framework for analyzing next-generation DNA sequencing data. Genome Res. 2010; 20:1297–1303.
7. Li H, Handsaker B, Wysoker A, Fennell T, Ruan J, Homer N, Marth G, Abecasis G, Durbin R, Genome Project Data Processing S. The Sequence Alignment/Map format and SAMtools. Bioinformatics. 2009; 25:2078–2079.
8. Carter SL, Cibulskis K, Helman E, McKenna A, Shen H, Zack T, Laird PW, Onofrio RC, Winckler W, Weir BA, Beroukhim R, Pellman D, Levine DA, et al. Absolute quantification of somatic DNA alterations in human cancer. Nat Biotechnol. 2012; 30:413–421.
9. Landau DA, Carter SL, Stojanov P, McKenna A, Stevenson K, Lawrence MS, Sougnez C, Stewart C, Sivachenko A, Wang L, Wan Y, Zhang W, Shukla SA, et al. Evolution and impact of subclonal mutations in chronic lymphocytic leukemia. Cell. 2013; 152:714–726.
10. Schwarz JM, Rödelsperger C, Schuelke M, Seelow D. MutationTaster evaluates disease-causing potential of sequence alterations. Nat Methods. 2010; 7:575–576.
11. Carter H, Chen S, Isik L, Tyekucheva S, Velculescu VE, Kinzler KW, Vogelstein B, Karchin R. Cancer-specific high-throughput annotation of somatic mutations: computational prediction of driver missense mutations. Cancer Res. 2009; 69:6660–6667.
12. Shihab HA, Gough J, Cooper DN, Stenson PD, Barker GL, Edwards KJ, Day IN, Gaunt TR. Predicting the functional, molecular, and phenotypic consequences of amino acid substitutions using hidden Markov models. Hum Mutat. 2013; 34:57–65.
13. Martelotto LG, Ng CK, De Filippo MR, Zhang Y, Piscuoglio S, Lim RS, Shen R, Norton L, Reis-Filho JS, Weigelt B. Benchmarking mutation effect prediction algorithms using functionally validated cancer-related missense mutations. Genome Biol. 2014; 15:484.
14. Dang VT, Kassahn KS, Marcos AE, Ragan MA. Identification of human haploinsufficient genes and their genomic proximity to segmental duplications. Eur J Hum Genet. 2008; 16:1350–1357.
15. Chang MT, Asthana S, Gao SP, Lee BH, Chapman JS, Kandoth C, Gao J, Socci ND, Solit DB, Olshen AB, Schultz N, Taylor BS. Identifying recurrent mutations in cancer reveals widespread lineage diversity and mutational specificity. Nat Biotechnol. 2016; 34:155–163.
16. Kandoth C, McLellan MD, Vandin F, Ye K, Niu B, Lu C, Xie M, Zhang Q, McMichael JF, Wyczalkowski MA, Leiserson MD, Miller CA, Welch JS, et al. Mutational landscape and significance across 12 major cancer types. Nature. 2013; 502:333–339.
17. Futreal PA, Coin L, Marshall M, Down T, Hubbard T, Wooster R, Rahman N, Stratton MR. A census of human cancer genes. Nat Rev Cancer. 2004; 4:177–183.
18. Lawrence MS, Stojanov P, Mermel CH, Robinson JT, Garraway LA, Golub TR, Meyerson M, Gabriel SB, Lander ES, Getz G. Discovery and saturation analysis of cancer genes across 21 tumour types. Nature. 2014; 505:495–501.
19. Wagner AH, Coffman AC, Ainscough BJ, Spies NC, Skidmore ZL, Campbell KM, Krysiak K, Pan D, McMichael JF, Eldred JM, Walker JR, Wilson RK, Mardis ER, et al. DGIdb 2.0: mining clinically relevant drug-gene interactions. Nucleic Acids Res. 2016; 44:D1036–1044.
20. Chakravarty D, Gao J, Phillips S, Kundra R, Zhang H, Wang J, Rudolph JE, Yaeger R, Soumerai T, Nissan MH, Chang MT, Chandarlapaty S, Traina TA, et al. OncoKB: A Precision Oncology Knowledge Base. JCO Precision Oncology. 2017: 1-16.
21. Murugaesu N, Wilson GA, Birkbak NJ, Watkins TB, McGranahan N, Kumar S, Abbassi-Ghadi N, Salm M, Mitter R, Horswell S, Rowan A, Phillimore B, Biggs J, et al. Tracking the genomic evolution of esophageal adenocarcinoma through neoadjuvant chemotherapy. Cancer Discov. 2015; 5:821–831.
22. Schliep KP. phangorn: phylogenetic analysis in R. Bioinformatics. 2011; 27:592–593.
