# Supplementary material for: Genetic heterogeneity and actionable mutations in HER2-positive primary breast cancers and their brain metastases
Source: Oncotarget. 2018 Apr 17;9(29):20617–30. doi: 10.18632/oncotarget.25041 (PMC5945519; doi:10.18632/oncotarget.25041)
Supplement: Supplementary file 2 [file oncotarget-09-20617-s002.doc]

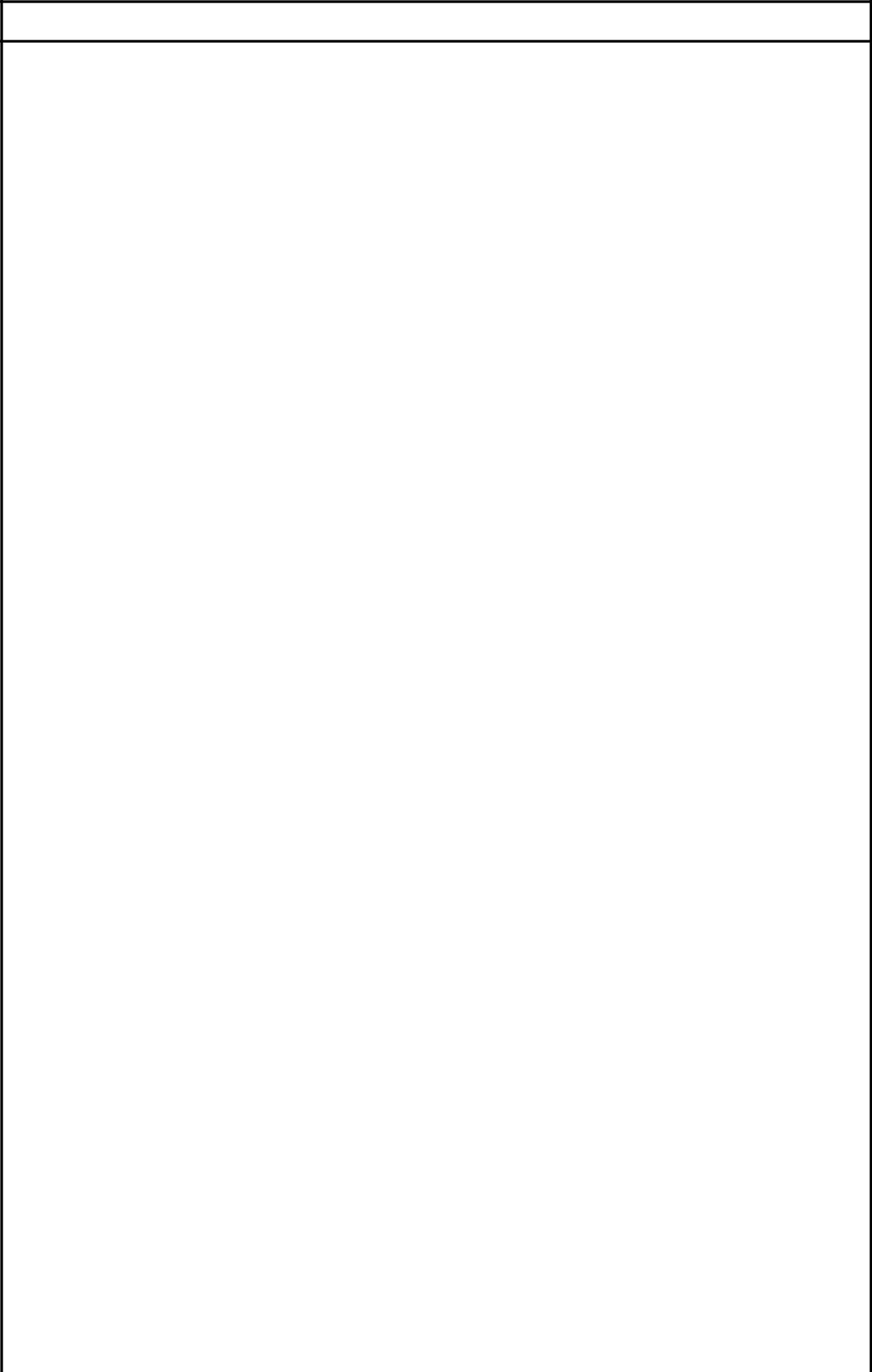


**Supplementary Table 1: List of 254 genes included in the targeted capture massively parallel sequencing platform**

|  | **The Cancer Genome Atlas (TCGA) Breast Invasive Carcinoma project** | | |  |
| --- | --- | --- | --- | --- |
|  | **All tumors: 825 cases** | | **HER2+ breast cancers: 91 cases** | |
| **Gene symbol** | **Number of cases with** | **Percentage of cases** | **Number of cases with** | **Percentage of cases** |
|  | **mutation** | **with mutation** | **mutation** | **with mutation** |
|  |  |  |  |  |
|  |  |  |  |  |
| ABCA13 | 14 | 2,8% | 2 | 2,2% |
| ABCB1 | 3 | 0,6% | 0 | 0,0% |
| ADAMTSL1 | 3 | 0,6% | 0 | 0,0% |
| AGFG2 | 3 | 0,6% | 2 | 2,2% |
| AHNAK2 | 9 | 1,8% | 3 | 3,3% |
| AK9 | 4 | 0,8% | 0 | 0,0% |
| AKAP9 | 9 | 1,8% | 1 | 1,1% |
| AKT1 | 12 | 2,4% | 0 | 0,0% |
| AKT2 | 1 | 0,2% | 0 | 0,0% |
| AKT3 | 3 | 0,6% | 0 | 0,0% |
| ANK3 | 10 | 2,0% | 5 | 5,5% |
| AOAH | 0 | 0,0% | 0 | 0,0% |
| APC | 3 | 0,6% | 1 | 1,1% |
| APOBEC1 | 1 | 0,2% | 0 | 0,0% |
| APOBEC2 | 1 | 0,2% | 0 | 0,0% |
| APOBEC3A | 1 | 0,2% | 0 | 0,0% |
| APOBEC3C | 0 | 0,0% | 0 | 0,0% |
| APOBEC3D | 0 | 0,0% | 0 | 0,0% |
| APOBEC3F | 0 | 0,0% | 0 | 0,0% |
| APOBEC3G | 1 | 0,2% | 0 | 0,0% |
| APOBEC3H | 0 | 0,0% | 0 | 0,0% |
| APOBEC4 | 1 | 0,2% | 0 | 0,0% |
| ARAF | 1 | 0,2% | 0 | 0,0% |
| ARID1A | 11 | 2,2% | 4 | 4,4% |
| ATM | 16 | 3,2% | 2 | 2,2% |
| ATN1 | 8 | 1,6% | 0 | 0,0% |
| ATR | 2 | 0,4% | 1 | 1,1% |
| ATRX | 9 | 1,8% | 2 | 2,2% |
| AURKA | 1 | 0,2% | 0 | 0,0% |
| AURKB | 0 | 0,0% | 0 | 0,0% |
| AURKC | 1 | 0,2% | 0 | 0,0% |
| BIRC5 | 0 | 0,0% | 0 | 0,0% |
| BRAF | 3 | 0,6% | 2 | 2,2% |
| BRCA1 | 15 | 3,0% | 1 | 1,1% |
| BRCA2 | 22 | 4,3% | 4 | 4,4% |
| BRIP1 | 8 | 1,6% | 2 | 2,2% |
| CACNA1A | 6 | 1,2% | 0 | 0,0% |
| CACNA1C | 6 | 1,2% | 3 | 3,3% |
| CACNA1E | 13 | 2,6% | 4 | 4,4% |
| CBFB | 8 | 1,6% | 0 | 0,0% |
| CDC25A | 1 | 0,2% | 1 | 1,1% |
| CDC25B | 3 | 0,6% | 1 | 1,1% |
| CDC25C | 1 | 0,2% | 0 | 0,0% |
| CDH1 | 33 | 6,5% | 4 | 4,4% |
| CDK1 | 0 | 0,0% | 0 | 0,0% |
| CDK4 | 0 | 0,0% | 0 | 0,0% |
| CDK6 | 1 | 0,2% | 0 | 0,0% |
| CDKN1A | 0 | 0,0% | 0 | 0,0% |
| CDKN1B | 5 | 1,0% | 0 | 0,0% |
| CDKN2A | 0 | 0,0% | 0 | 0,0% |
| CDKN2B | 0 | 0,0% | 0 | 0,0% |
| CEP164 | 4 | 0,8% | 1 | 1,1% |
| CHD4 | 7 | 1,4% | 3 | 3,3% |
| CHD6 | 7 | 1,4% | 0 | 0,0% |
| CHEK1 | 0 | 0,0% | 0 | 0,0% |
| CHEK2 | 4 | 0,8% | 0 | 0,0% |
| COL12A1 | 10 | 2,0% | 3 | 3,3% |
| CTCF | 13 | 2,6% | 2 | 2,2% |
| CTNNB1 | 0 | 0,0% | 0 | 0,0% |

| 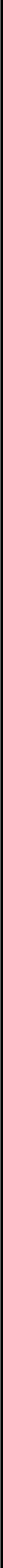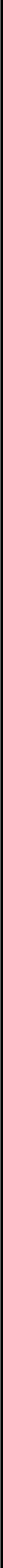CUBN | 7 | 1,4% | 0 | 0,0% |
| --- | --- | --- | --- | --- |
| DCHS2 | 8 | 1,6% | 1 | 1,1% |
| DCLRE1C | 5 | 1,0% | 2 | 2,2% |
| DEPTOR | 0 | 0,0% | 0 | 0,0% |
| DMC1 | 2 | 0,4% | 0 | 0,0% |
| DOCK11 | 9 | 1,8% | 2 | 2,2% |
| EGFR | 4 | 0,8% | 1 | 1,1% |
| EIF4A2 | 4 | 0,8% | 1 | 1,1% |
| EME1 | 0 | 0,0% | 0 | 0,0% |
| EME2 | 0 | 0,0% | 0 | 0,0% |
| EPPK1 | 1 | 0,2% | 0 | 0,0% |
| ERBB2 | 7 | 1,4% | 3 | 3,3% |
| ERBB3 | 8 | 1,6% | 5 | 5,5% |
| ERBB4 | 6 | 1,2% | 1 | 1,1% |
| ERCC1 | 1 | 0,2% | 0 | 0,0% |
| ERCC2 | 0 | 0,0% | 0 | 0,0% |
| ERCC3 | 2 | 0,4% | 1 | 1,1% |
| ERCC5 | 4 | 0,8% | 0 | 0,0% |
| ESR1 | 2 | 0,4% | 1 | 1,1% |
| ESR2 | 2 | 0,4% | 0 | 0,0% |
| FAM157B | 1 | 0,2% | 1 | 1,1% |
| FANCA | 7 | 1,4% | 1 | 1,1% |
| FANCB | 0 | 0,0% | 0 | 0,0% |
| FANCC | 1 | 0,2% | 0 | 0,0% |
| FANCD2 | 3 | 0,6% | 0 | 0,0% |
| FANCE | 1 | 0,2% | 0 | 0,0% |
| FANCF | 1 | 0,2% | 0 | 0,0% |
| FANCG | 1 | 0,2% | 0 | 0,0% |
| FANCI | 3 | 0,6% | 0 | 0,0% |
| FANCL | 1 | 0,2% | 1 | 1,1% |
| FANCM | 2 | 0,4% | 1 | 1,1% |
| FBN1 | 7 | 1,4% | 0 | 0,0% |
| FGFR1 | 0 | 0,0% | 0 | 0,0% |
| FGFR2 | 4 | 0,8% | 1 | 1,1% |
| FGFR3 | 1 | 0,2% | 0 | 0,0% |
| FGFR4 | 3 | 0,6% | 2 | 2,2% |
| FMN2 | 8 | 1,6% | 1 | 1,1% |
| FOXA1 | 8 | 1,6% | 3 | 3,3% |
| FOXC2 | 0 | 0,0% | 0 | 0,0% |
| FRG1B | 0 | 0,0% | 0 | 0,0% |
| GATA3 | 54 | 10,7% | 10 | 11,0% |
| GPS2 | 6 | 1,2% | 2 | 2,2% |
| GRB2 | 1 | 0,2% | 0 | 0,0% |
| GRIN2A | 10 | 2,0% | 0 | 0,0% |
| GRIN2B | 8 | 1,6% | 2 | 2,2% |
| HECW1 | 8 | 1,6% | 2 | 2,2% |
| HERC2 | 6 | 1,2% | 1 | 1,1% |
| HIF1A | 1 | 0,2% | 1 | 1,1% |
| HIST1H3B | 4 | 0,8% | 1 | 1,1% |
| HRAS | 0 | 0,0% | 0 | 0,0% |
| HRNR | 9 | 1,8% | 2 | 2,2% |
| HSP90AA1 | 2 | 0,4% | 0 | 0,0% |
| HSP90AB1 | 2 | 0,4% | 0 | 0,0% |
| HUWE1 | 9 | 1,8% | 3 | 3,3% |
| IGF1R | 2 | 0,4% | 0 | 0,0% |
| INPP4B | 4 | 0,8% | 0 | 0,0% |
| IRS1 | 1 | 0,2% | 1 | 1,1% |
| JAK1 | 2 | 0,4% | 1 | 1,1% |
| JAK2 | 5 | 1,0% | 2 | 2,2% |
| KIT | 5 | 1,0% | 0 | 0,0% |
| KMT2C | 37 | 7,3% | 8 | 8,8% |
| KMT2D | 8 | 1,6% | 2 | 2,2% |
| KRAS | 4 | 0,8% | 0 | 0,0% |
| LAMA1 | 7 | 1,4% | 1 | 1,1% |
| LAMA5 | 7 | 1,4% | 2 | 2,2% |
| MACF1 | 11 | 2,2% | 2 | 2,2% |
| MAP1A | 10 | 2,0% | 4 | 4,4% |
| MAP2K1 | 1 | 0,2% | 0 | 0,0% |

| 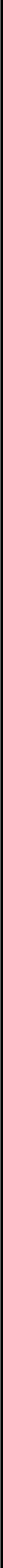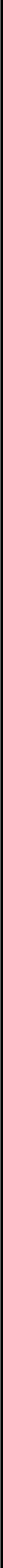MAP2K2 | 1 | 0,2% | 0 | 0,0% |
| --- | --- | --- | --- | --- |
| MAP2K3 | 1 | 0,2% | 1 | 1,1% |
| MAP2K4 | 21 | 4,1% | 0 | 0,0% |
| MAP2K6 | 0 | 0,0% | 0 | 0,0% |
| MAP3K1 | 39 | 7,7% | 5 | 5,5% |
| MAP3K10 | 4 | 0,8% | 1 | 1,1% |
| MAP3K4 | 4 | 0,8% | 1 | 1,1% |
| MAP4K4 | 1 | 0,2% | 0 | 0,0% |
| MAPK1 | 1 | 0,2% | 0 | 0,0% |
| MAPK8 | 2 | 0,4% | 0 | 0,0% |
| MAPK9 | 0 | 0,0% | 0 | 0,0% |
| MDM2 | 2 | 0,4% | 0 | 0,0% |
| MDN1 | 11 | 2,2% | 3 | 3,3% |
| MED12 | 8 | 1,6% | 1 | 1,1% |
| MET | 3 | 0,6% | 0 | 0,0% |
| MGAM | 6 | 1,2% | 2 | 2,2% |
| MGMT | 2 | 0,4% | 0 | 0,0% |
| MLH1 | 1 | 0,2% | 0 | 0,0% |
| MLH3 | 3 | 0,6% | 1 | 1,1% |
| MRE11A | 1 | 0,2% | 1 | 1,1% |
| MSH2 | 2 | 0,4% | 0 | 0,0% |
| MSH3 | 2 | 0,4% | 0 | 0,0% |
| MSH5 | 1 | 0,2% | 0 | 0,0% |
| MSH6 | 4 | 0,8% | 2 | 2,2% |
| MST1L | 5 | 1,0% | 0 | 0,0% |
| MTOR | 8 | 1,6% | 3 | 3,3% |
| MUTYH | 2 | 0,4% | 0 | 0,0% |
| MXRA5 | 7 | 1,4% | 1 | 1,1% |
| MYB | 8 | 1,6% | 1 | 1,1% |
| NBEAL2 | 6 | 1,2% | 0 | 0,0% |
| NBN | 2 | 0,4% | 0 | 0,0% |
| NBPF1 | 1 | 0,2% | 1 | 1,1% |
| NCOA3 | 6 | 1,2% | 3 | 3,3% |
| NCOR1 | 17 | 3,4% | 1 | 1,1% |
| NCOR2 | 3 | 0,6% | 0 | 0,0% |
| NEB | 19 | 3,7% | 2 | 2,2% |
| NF1 | 14 | 2,8% | 1 | 1,1% |
| NF2 | 2 | 0,4% | 1 | 1,1% |
| NR1H2 | 3 | 0,6% | 1 | 1,1% |
| NRAS | 0 | 0,0% | 0 | 0,0% |
| PALB2 | 0 | 0,0% | 0 | 0,0% |
| PARP1 | 1 | 0,2% | 0 | 0,0% |
| PARP2 | 1 | 0,2% | 1 | 1,1% |
| PARP3 | 2 | 0,4% | 0 | 0,0% |
| PAXIP1 | 3 | 0,6% | 0 | 0,0% |
| PCNXL2 | 9 | 1,8% | 1 | 1,1% |
| PDGFRA | 3 | 0,6% | 1 | 1,1% |
| PDGFRB | 2 | 0,4% | 0 | 0,0% |
| PGR | 2 | 0,4% | 0 | 0,0% |
| PIK3CA | 178 | 35,1% | 27 | 29,7% |
| PIK3CB | 4 | 0,8% | 2 | 2,2% |
| PIK3R1 | 13 | 2,6% | 5 | 5,5% |
| PLEC | 7 | 1,4% | 1 | 1,1% |
| PLK1 | 2 | 0,4% | 1 | 1,1% |
| PLXNA4 | 8 | 1,6% | 2 | 2,2% |
| PMS1 | 5 | 1,0% | 1 | 1,1% |
| PMS2 | 1 | 0,2% | 0 | 0,0% |
| POLB | 1 | 0,2% | 0 | 0,0% |
| POLD1 | 0 | 0,0% | 0 | 0,0% |
| POLE | 5 | 1,0% | 0 | 0,0% |
| POLH | 2 | 0,4% | 0 | 0,0% |
| POLQ | 6 | 1,2% | 2 | 2,2% |
| PRKCA | 0 | 0,0% | 0 | 0,0% |
| PRKCB | 3 | 0,6% | 0 | 0,0% |
| PRKCD | 0 | 0,0% | 0 | 0,0% |
| PRKCG | 2 | 0,4% | 1 | 1,1% |
| PRKD1 | 2 | 0,4% | 0 | 0,0% |
| PTCH1 | 6 | 1,2% | 0 | 0,0% |

| PTEN | 18 | 3,6% | 2 | 2,2% |
| --- | --- | --- | --- | --- |
| PTK2 | 3 | 0,6% | 0 | 0,0% |
| RAD50 | 2 | 0,4% | 1 | 1,1% |
| RAD51 | 1 | 0,2% | 0 | 0,0% |
| RAD51B | 0 | 0,0% | 0 | 0,0% |
| RAD51C | 2 | 0,4% | 1 | 1,1% |
| RAD51D | 1 | 0,2% | 0 | 0,0% |
| RAD52 | 0 | 0,0% | 0 | 0,0% |
| RAD54B | 4 | 0,8% | 2 | 2,2% |
| RAD54L | 2 | 0,4% | 1 | 1,1% |
| RAF1 | 1 | 0,2% | 0 | 0,0% |
| RB1 | 9 | 1,8% | 1 | 1,1% |
| RBBP8 | 0 | 0,0% | 0 | 0,0% |
| RELN | 13 | 2,6% | 4 | 4,4% |
| RICTOR | 3 | 0,6% | 1 | 1,1% |
| RIF1 | 6 | 1,2% | 1 | 1,1% |
| RPGR | 10 | 2,0% | 0 | 0,0% |
| RPS6KB1 | 0 | 0,0% | 0 | 0,0% |
| RPTOR | 2 | 0,4% | 0 | 0,0% |
| RUNX1 | 17 | 3,4% | 2 | 2,2% |
| SAAL1 | 5 | 1,0% | 0 | 0,0% |
| SF3B1 | 10 | 2,0% | 2 | 2,2% |
| SHC1 | 1 | 0,2% | 1 | 1,1% |
| SHROOM4 | 5 | 1,0% | 1 | 1,1% |
| SMO | 2 | 0,4% | 0 | 0,0% |
| SOS1 | 4 | 0,8% | 2 | 2,2% |
| SPEN | 13 | 2,6% | 5 | 5,5% |
| SPRY1 | 0 | 0,0% | 0 | 0,0% |
| SPTA1 | 14 | 2,8% | 3 | 3,3% |
| SRCAP | 9 | 1,8% | 2 | 2,2% |
| STAT1 | 1 | 0,2% | 0 | 0,0% |
| STAT3 | 1 | 0,2% | 0 | 0,0% |
| SVEP1 | 11 | 2,2% | 3 | 3,3% |
| TBL1XR1 | 8 | 1,6% | 2 | 2,2% |
| TBX3 | 13 | 2,6% | 1 | 1,1% |
| TENM1 | 10 | 2,0% | 3 | 3,3% |
| TGFBR1 | 2 | 0,4% | 1 | 1,1% |
| TGFBR2 | 3 | 0,6% | 0 | 0,0% |
| TGFBR3 | 2 | 0,4% | 0 | 0,0% |
| TOP2A | 1 | 0,2% | 0 | 0,0% |
| TP53 | 187 | 36,9% | 45 | 49,5% |
| TP53BP1 | 3 | 0,6% | 0 | 0,0% |
| TSC1 | 3 | 0,6% | 2 | 2,2% |
| TSC2 | 2 | 0,4% | 0 | 0,0% |
| TYK2 | 4 | 0,8% | 0 | 0,0% |
| UBR4 | 12 | 2,4% | 3 | 3,3% |
| USP36 | 3 | 0,6% | 0 | 0,0% |
| WDFY3 | 9 | 1,8% | 4 | 4,4% |
| XBP1 | 2 | 0,4% | 0 | 0,0% |
| XPA | 0 | 0,0% | 0 | 0,0% |
| XPC | 0 | 0,0% | 0 | 0,0% |
| XRCC1 | 1 | 0,2% | 0 | 0,0% |
| XRCC2 | 0 | 0,0% | 0 | 0,0% |
| XRCC3 | 0 | 0,0% | 0 | 0,0% |
| ZFHX3 | 10 | 2,0% | 4 | 4,4% |
| ZFHX4 | 16 | 3,2% | 4 | 4,4% |
| ZFP36L1 | 7 | 1,4% | 2 | 2,2% |
| ZNF384 | 0 | 0,0% | 0 | 0,0% |
| ZNF703 | 0 | 0,0% | 0 | 0,0% |
