# Supplementary material for: Genetic heterogeneity and actionable mutations in HER2-positive primary breast cancers and their brain metastases
Source: Oncotarget. 2018 Apr 17;9(29):20617–30. doi: 10.18632/oncotarget.25041 (PMC5945519; doi:10.18632/oncotarget.25041)
Supplement: Supplementary file 3 [file oncotarget-09-20617-s003.doc]

Supplementary Table 4: Somatic single nucleotide variants (SNVs) and insertion/deletions (indels) present in the samples analyzed.

| **Case** | **Sample** | **Gene** | **Amino acid** | **Effect** | **Mutant allele franction** | **Depth** | **Chromosome** | **Genomic Position** | **Reference base** | **Alternative base** | **Loss of heterozygozity** | **Mutation Taster** | **CHASM** | **FATHMM** | **Kandoth *et al* . Nature 2013** | **Cancer Census (COSMIC)** | **Lawrence *et al*. Nature 2014** | **Hotspot** | **Haploinsufficient** | **Pathogenicity** | **Cancer cell fraction (CCF) (ABSOLUTE)** | **Probability of mutation being clonal** | **Lower bound of 95% confidence interval of CCF** | **Clonal/Subclonal mutation** | **Sequencing** | **Private to or enriched for in the brain metastasis** | **Shared mutations associated with secondary LOH only in the brain metastasis** |
| --- | --- | --- | --- | --- | --- | --- | --- | --- | --- | --- | --- | --- | --- | --- | --- | --- | --- | --- | --- | --- | --- | --- | --- | --- | --- | --- | --- |
| 1 | Case 1: brain metastasis | HRNR | P573L | missense_mutation | 27,2% | 633 | 1 | 152.192.387 | G | A | No LOH | N | Passenger | PASSENGER/OTHER |  |  |  |  |  | Likely passenger | 100% | 0,0202 | 94,18% | Clonal | Targeted and amplicon sequencing | private to the metastasis |  |
| 1 | Case 1: brain metastasis | PIK3CA | E542K | missense_mutation | 41,8% | 691 | 3 | 178.936.082 | G | A | No LOH | D | Driver | CANCER | TRUE | TRUE | TRUE | Hotspot |  | Likely pathogenic | 100% | 0,9836 | 98,03% | Clonal | Targeted sequencing |  |  |
| 1 | Case 1: brain metastasis | WDFY3 | K2603N | missense_mutation | 35,3% | 374 | 4 | 85.638.115 | C | G | No LOH | D | Passenger | CANCER |  |  |  |  |  | Likely pathogenic | 100% | 0,9339 | 86,80% | Clonal | Targeted and amplicon sequencing |  |  |
| 1 | Case 1: brain metastasis | DEPTOR | P325L | missense_mutation | 19,6% | 814 | 8 | 121.019.092 | C | T | No LOH | D | Passenger | PASSENGER/OTHER |  |  |  |  |  | Indeterminate | 98% | 0,8799 | 83,10% | Clonal | Targeted and amplicon sequencing | private to the metastasis |  |
| 1 | Case 1: brain metastasis | EPPK1 | G1464fs | frameshift_deletion | 12,0% | 801 | 8 | 144.943.029 | ACC | A | No LOH | . | . | . | TRUE |  |  |  |  | Indeterminate | 59% | 0,0000 | 48,71% | Subclonal | Targeted sequencing | private to the metastasis |  |
| 1 | Case 1: brain metastasis | CACNA1C | A317V | missense_mutation | 40,5% | 766 | 12 | 2.602.389 | C | T | No LOH | D | Passenger | PASSENGER/OTHER |  |  |  |  |  | Indeterminate | 100% | 0,0237 | 98,13% | Clonal | Targeted and amplicon sequencing | private to the metastasis |  |
| 1 | Case 1: brain metastasis | CACNA1C | E1948K | missense_mutation | 6,0% | 671 | 12 | 2.794.921 | G | A | No LOH | N | Passenger | PASSENGER/OTHER |  |  |  |  |  | Likely passenger | 36% | 0,0000 | 25,81% | Subclonal | Targeted and amplicon sequencing | enriched for in the metastasis |  |
| 1 | Case 1: brain metastasis | CDH1 | Q346* | nonsense_mutation | 75,7% | 486 | 16 | 68.846.065 | C | T | LOH | A | . | . | TRUE | TRUE | TRUE |  |  | Likely pathogenic | 100% | 0,0482 | 98,54% | Clonal | Targeted and amplicon sequencing |  |  |
| 1 | Case 1: brain metastasis | TP53 | Y236C | missense_mutation | 70,7% | 482 | 17 | 7.577.574 | T | C | LOH | D | Driver | CANCER | TRUE | TRUE | TRUE | Hotspot | TRUE | Likely pathogenic | 100% | 0,9752 | 98,33% | Clonal | Targeted and amplicon sequencing | private to the metastasis |  |
| 1 | Case 1: brain metastasis | MED12 | E1740Q | missense_mutation | 21,4% | 595 | X | 70.356.323 | G | C | No LOH | D | Passenger | PASSENGER/OTHER |  | TRUE | TRUE |  |  | Indeterminate | 100% | 0,9289 | 85,88% | Clonal | Targeted and amplicon sequencing | private to the metastasis |  |
| 1 | Case 1: primary breast tumor | PIK3CA | E542K | missense_mutation | 28,8% | 462 | 3 | 178.936.082 | G | A | No LOH | D | Driver | CANCER | TRUE | TRUE | TRUE | Hotspot |  | Likely pathogenic | 100% | 0,9353 | 88,80% | Clonal | Targeted sequencing |  |  |
| 1 | Case 1: primary breast tumor | WDFY3 | K2603N | missense_mutation | 30,9% | 480 | 4 | 85.638.115 | C | G | No LOH | D | Passenger | CANCER |  |  |  |  |  | Likely pathogenic | 100% | 0,8671 | 91,56% | Clonal | Targeted and amplicon sequencing |  |  |
| 1 | Case 1: primary breast tumor | CACNA1C | E1948K | missense_mutation | 2,21% | 2539 | 12 | 2.794.921 | G | A | No LOH | N | Passenger | PASSENGER/OTHER |  |  |  |  |  | Likely passenger | 9% | 0,0000 | 6,08% | Subclonal | Amplicon sequencing | enriched for in the metastasis |  |
| 1 | Case 1: primary breast tumor | CDH1 | Q346* | nonsense_mutation | 42,7% | 386 | 16 | 68.846.065 | C | T | LOH | A | . | . | TRUE | TRUE | TRUE |  |  | Likely pathogenic | 100% | 0,1328 | 96% | Clonal | Targeted and amplicon sequencing |  |  |
| 12 | Case 12: brain metastasis # 1 | NBPF1 | Q974* | nonsense_mutation | 50,0% | 10 | 1 | 16.892.277 | G | A | No LOH | . | . | . |  | TRUE |  |  |  | Indeterminate | 100% | 0,8371 | 47% | Clonal | Targeted sequencing | private to the metastasis |  |
| 12 | Case 12: brain metastasis # 1 | UBR4 | E1114* | nonsense_mutation | 26,2% | 127 | 1 | 19.499.539 | C | A | No LOH | D | . | . |  |  |  |  |  | Indeterminate | 86% | 0,6246 | 62% | Clonal | Targeted and amplicon sequencing | private to the metastasis |  |
| 12 | Case 12: brain metastasis # 1 | RAF1 | D23H | missense_mutation | 40,1% | 167 | 3 | 12.660.154 | C | G | No LOH | D | Passenger | CANCER |  | TRUE | TRUE |  |  | Likely pathogenic | 100% | 0,7160 | 88% | Clonal | Targeted and amplicon sequencing | private to the metastasis |  |
| 12 | Case 12: brain metastasis # 1 | RAF1 | D19Y | missense_mutation | 37,0% | 165 | 3 | 12.660.166 | C | A | No LOH | D | Passenger | CANCER |  | TRUE | TRUE |  |  | Likely pathogenic | 100% | 0,8996 | 85% | Clonal | Targeted and amplicon sequencing | private to the metastasis |  |
| 12 | Case 12: brain metastasis # 1 | PIK3CA | H1047R | missense_mutation | 32,1% | 106 | 3 | 178.952.085 | A | G | No LOH | D | Driver | CANCER | TRUE | TRUE | TRUE | Hotspot |  | Likely pathogenic | 100% | 0,8518 | 73% | Clonal | Targeted sequencing | enriched for in the metastasis |  |
| 12 | Case 12: brain metastasis # 1 | WDFY3 | Q2808H | missense_mutation | 26,3% | 76 | 4 | 85.625.509 | C | G | No LOH | D | Driver | PASSENGER/OTHER |  |  |  |  |  | Likely pathogenic | 87% | 0,6532 | 57% | Clonal | Targeted and amplicon sequencing | private to the metastasis |  |
| 12 | Case 12: brain metastasis # 1 | RICTOR | D861Y | missense_mutation | 26,5% | 117 | 5 | 38.955.725 | C | A | No LOH | D | Passenger | PASSENGER/OTHER |  |  |  |  |  | Indeterminate | 88% | 0,6627 | 62% | Clonal | Targeted and amplicon sequencing | private to the metastasis |  |
| 12 | Case 12: brain metastasis # 1 | KMT2C | K2769N | missense_mutation | 16,7% | 164 | 7 | 151.874.231 | C | G | No LOH | D | Passenger | PASSENGER/OTHER | TRUE | TRUE | TRUE |  |  | Indeterminate | 71% | 0,2104 | 49% | Subclonal | Targeted sequencing | private to the metastasis |  |
| 12 | Case 12: brain metastasis # 1 | ZNF703 | S389W | missense_mutation | 8,5% | 757 | 8 | 37.555.585 | C | G | No LOH | D | Driver | PASSENGER/OTHER |  |  |  |  |  | Likely pathogenic | 36% | 0,0000 | 28% | Subclonal | Targeted and amplicon sequencing | private to the metastasis |  |
| 12 | Case 12: brain metastasis # 1 | ZFHX3 | Q2952E | missense_mutation | 32,2% | 345 | 16 | 72.827.727 | G | C | No LOH | D | Passenger | PASSENGER/OTHER |  | TRUE |  |  |  | Indeterminate | 100% | 0,9249 | 86% | Clonal | Targeted and amplicon sequencing | private to the metastasis |  |
| 12 | Case 12: brain metastasis # 1 | TP53 | H214R | missense_mutation | 82,0% | 200 | 17 | 7.578.208 | T | C | LOH | D | Driver | CANCER | TRUE | TRUE | TRUE | Hotspot | TRUE | Likely pathogenic | 100% | 0,9558 | 97% | Clonal | Targeted and amplicon sequencing | enriched for in the metastasis |  |
| 12 | Case 12: brain metastasis # 1 | TENM1 | S1855L | missense_mutation | 17,04% | 21353 | X | 123.526.026 | G | A | No LOH | D | Passenger | PASSENGER/OTHER |  |  |  |  |  | Indeterminate | 39% | 0,0000 | 38% | Subclonal | Amplicon sequencing |  |  |
| 12 | Case 12: brain metastasis # 2 | UBR4 | E1114* | nonsense_mutation | 14,5% | 76 | 1 | 19.499.539 | C | A | No LOH | D | . | . |  |  |  |  |  | Indeterminate | 70% | 0,3606 | 39% | Subclonal | Targeted and amplicon sequencing | private to the metastasis |  |
| 12 | Case 12: brain metastasis # 2 | SOS1 | R1041C | missense_mutation | 5,4% | 93 | 2 | 39.222.489 | G | A | No LOH | D | Passenger | PASSENGER/OTHER |  |  |  |  |  | Indeterminate | 37% | 0,0271 | 16% | Subclonal | Targeted and amplicon sequencing | private to the metastasis |  |
| 12 | Case 12: brain metastasis # 2 | RAF1 | D23H | missense_mutation | 47,4% | 97 | 3 | 12.660.154 | C | G | No LOH | D | Passenger | CANCER |  | TRUE | TRUE |  |  | Likely pathogenic | 100% | 0,3573 | 91% | Clonal | Targeted and amplicon sequencing | private to the metastasis |  |
| 12 | Case 12: brain metastasis # 2 | RAF1 | D19Y | missense_mutation | 47,9% | 96 | 3 | 12.660.166 | C | A | No LOH | D | Passenger | CANCER |  | TRUE | TRUE |  |  | Likely pathogenic | 100% | 0,3443 | 91% | Clonal | Targeted and amplicon sequencing | private to the metastasis |  |
| 12 | Case 12: brain metastasis # 2 | NBEAL2 | T1422A | missense_mutation | 12,5% | 88 | 3 | 47.041.853 | A | G | LOH | N | Passenger | PASSENGER/OTHER |  |  |  |  |  | Likely passenger | 48% | 0,0140 | 27% | Subclonal | Targeted and amplicon sequencing | private to the metastasis |  |
| 12 | Case 12: brain metastasis # 2 | PIK3CB | S769* | nonsense_mutation | 23,1% | 65 | 3 | 138.403.476 | G | C | No LOH | A | . | . |  |  |  |  |  | Indeterminate | 100% | 0,8136 | 65% | Clonal | Targeted sequencing | private to the metastasis |  |
| 12 | Case 12: brain metastasis # 2 | PIK3CA | H1047R | missense_mutation | 19,0% | 79 | 3 | 178.952.085 | A | G | No LOH | D | Driver | CANCER | TRUE | TRUE | TRUE | Hotspot |  | Likely pathogenic | 92% | 0,6527 | 54% | Clonal | Targeted sequencing | enriched for in the metastasis |  |
| 12 | Case 12: brain metastasis # 2 | WDFY3 | Q2808H | missense_mutation | 12,2% | 98 | 4 | 85.625.509 | C | G | No LOH | D | Driver | PASSENGER/OTHER |  |  |  |  |  | Likely pathogenic | 84% | 0,5602 | 46% | Clonal | Targeted and amplicon sequencing | private to the metastasis |  |
| 12 | Case 12: brain metastasis # 2 | RICTOR | D861Y | missense_mutation | 29,8% | 94 | 5 | 38.955.725 | C | A | No LOH | D | Passenger | PASSENGER/OTHER |  |  |  |  |  | Indeterminate | 100% | 0,9362 | 81% | Clonal | Targeted sequencing | private to the metastasis |  |
| 12 | Case 12: brain metastasis # 2 | ZFHX3 | Q2952E | missense_mutation | 14,1% | 99 | 16 | 72.827.727 | G | C | No LOH | D | Passenger | PASSENGER/OTHER |  | TRUE |  |  |  | Indeterminate | 82% | 0,5475 | 48% | Clonal | Targeted sequencing | private to the metastasis |  |
| 12 | Case 12: brain metastasis # 2 | TP53 | H214R | missense_mutation | 60,0% | 75 | 17 | 7.578.208 | T | C | LOH | D | Driver | CANCER | TRUE | TRUE | TRUE | Hotspot | TRUE | Likely pathogenic | 100% | 0,9243 | 90% | Clonal | Targeted and amplicon sequencing | enriched for in the metastasis |  |
| 12 | Case 12: brain metastasis # 2 | PRKCA | K91T | missense_mutation | 11,7% | 111 | 17 | 64.492.385 | A | C | LOH | D | Driver | PASSENGER/OTHER |  |  |  |  |  | Likely pathogenic | 68% | 0,3058 | 40% | Subclonal | Targeted sequencing | private to the metastasis |  |
| 12 | Case 12: brain metastasis # 2 | MAP2K6 | L229H | missense_mutation | 8,5% | 117 | 17 | 67.521.064 | T | A | LOH | D | Driver | CANCER |  |  |  |  |  | Likely pathogenic | 75% | 0,4636 | 40% | Subclonal | Targeted and amplicon sequencing | private to the metastasis |  |
| 12 | Case 12: brain metastasis # 2 | CACNA1A | L1377R | missense_mutation | 9,6% | 83 | 19 | 13.372.396 | A | C | No LOH | D | Driver | PASSENGER/OTHER |  |  |  |  |  | Likely pathogenic | 56% | 0,1761 | 29% | Subclonal | Targeted and amplicon sequencing | private to the metastasis |  |
| 12 | Case 12: brain metastasis # 2 | CHD6 | K2587T | missense_mutation | 5,8% | 103 | 20 | 40.033.621 | T | G | No LOH | D | Driver | PASSENGER/OTHER |  |  |  |  |  | Likely pathogenic | 34% | 0,0050 | 15% | Subclonal | Targeted sequencing | private to the metastasis |  |
| 12 | Case 12: brain metastasis # 2 | MED12 | P561S | missense_mutation | 9,0% | 100 | X | 70.343.507 | C | T | No LOH | D | Passenger | PASSENGER/OTHER |  | TRUE | TRUE |  |  | Indeterminate | 52% | 0,0927 | 28% | Subclonal | Targeted and amplicon sequencing | private to the metastasis |  |
| 12 | Case 12: brain metastasis # 2 | TENM1 | S1855L | missense_mutation | 13,7% | 73 | X | 123.526.026 | G | A | No LOH | D | Passenger | PASSENGER/OTHER |  |  |  |  |  | Indeterminate | 66% | 0,3056 | 36% | Subclonal | Targeted and amplicon sequencing |  |  |
| 12 | Case 12: primary breast tumor | PIK3CA | H1047R | missense_mutation | 1,44% | 4373 | 3 | 178.952.085 | A | G | No LOH | D | Driver | CANCER | TRUE | TRUE | TRUE | Hotspot |  | Likely pathogenic | 9% | 0,0000 | 6% | Subclonal | Amplicon sequencing | enriched for in the metastasis |  |
| 12 | Case 12: primary breast tumor | AKAP9 | Q1130E | missense_mutation | 10,0% | 50 | 7 | 91.641.812 | C | G | No LOH | D | Passenger | PASSENGER/OTHER |  |  | TRUE |  |  | Indeterminate | 71% | 0,3994 | 29% | Subclonal | Targeted sequencing |  |  |
| 12 | Case 12: primary breast tumor | CDH1 | Q23* | nonsense_mutation | 7,0% | 115 | 16 | 68.772.218 | C | T | No LOH | A | . | . | TRUE | TRUE | TRUE | Hotspot |  | Likely pathogenic | 49% | 0,0679 | 25% | Subclonal | Targeted and amplicon sequencing |  |  |
| 12 | Case 12: primary breast tumor | TP53 | H214R | missense_mutation | 8,5% | 59 | 17 | 7.578.208 | T | C | LOH | D | Driver | CANCER | TRUE | TRUE | TRUE | Hotspot | TRUE | Likely pathogenic | 52% | 0,1761 | 22% | Subclonal | Targeted and amplicon sequencing | enriched for in the metastasis |  |
| 12 | Case 12: primary breast tumor | DOCK11 | S1189F | missense_mutation | 13,2% | 76 | X | 117.758.596 | C | T | No LOH | D | Passenger | PASSENGER/OTHER |  |  |  |  |  | Indeterminate | 80% | 0,4789 | 42% | Subclonal | Targeted sequencing |  |  |
| 12 | Case 12: primary breast tumor | TENM1 | S1855L | missense_mutation | 18,79% | 26106 | X | 123.526.026 | G | A | LOH | D | Passenger | PASSENGER/OTHER |  |  |  |  |  | Indeterminate | 100% | 0,0000 | 99% | Clonal | Amplicon sequencing |  |  |
| 14 | Case 14: brain metastasis | NBPF1 | E168K | missense_mutation | 13,3% | 731 | 1 | 16.914.284 | C | T | LOH | . | Passenger | PASSENGER/OTHER |  | TRUE |  |  |  | Indeterminate | 63% | 0,0000 | 51% | Subclonal | Targeted and amplicon sequencing | private to the metastasis |  |
| 14 | Case 14: brain metastasis | ARID1A | S138L | missense_mutation | 8,2% | 477 | 1 | 27.023.307 | C | T | LOH | D | Passenger | PASSENGER/OTHER | TRUE | TRUE | TRUE |  |  | Indeterminate | 47% | 0,0000 | 34% | Subclonal | Targeted sequencing | private to the metastasis |  |
| 14 | Case 14: brain metastasis | CACNA1E | L1575V | missense_mutation | 12,2% | 790 | 1 | 181.732.575 | C | G | No LOH | D | Passenger | PASSENGER/OTHER |  |  |  |  |  | Indeterminate | 94% | 0,8423 | 76% | Clonal | Targeted and amplicon sequencing | enriched for in the metastasis |  |
| 14 | Case 14: brain metastasis | PARP1 | D217H | missense_mutation | 3,8% | 811 | 1 | 226.576.425 | C | G | No LOH | D | Passenger | PASSENGER/OTHER |  |  |  |  |  | Indeterminate | 30% | 0,0000 | 20% | Subclonal | Targeted and amplicon sequencing | private to the metastasis |  |
| 14 | Case 14: brain metastasis | SOS1 | D169H | missense_mutation | 15,7% | 606 | 2 | 39.283.848 | C | G | No LOH | D | Passenger | PASSENGER/OTHER |  |  |  |  |  | Indeterminate | 90% | 0,7553 | 74% | Clonal | Targeted and amplicon sequencing |  |  |
| 14 | Case 14: brain metastasis | MLH1 | R100Q | missense_mutation | 4,0% | 401 | 3 | 37.042.537 | G | A | LOH | D | Driver | CANCER |  | TRUE | TRUE |  |  | Likely pathogenic | 15% | 0,0000 | 9% | Subclonal | Targeted and amplicon sequencing | private to the metastasis |  |
| 14 | Case 14: brain metastasis | ATR | E1878Q | missense_mutation | 36,8% | 782 | 3 | 142.215.961 | C | G | No LOH | D | Passenger | CANCER | TRUE | TRUE |  |  | TRUE | Likely pathogenic | 100% | 0,9786 | 98% | Clonal | Targeted and amplicon sequencing | private to the metastasis |  |
| 14 | Case 14: brain metastasis | PIK3R1 | D529H | missense_mutation | 18,9% | 630 | 5 | 67.590.992 | G | C | No LOH | D | Passenger | PASSENGER/OTHER | TRUE | TRUE | TRUE |  |  | Indeterminate | 100% | 0,9462 | 86% | Clonal | Targeted and amplicon sequencing | private to the metastasis |  |
| 14 | Case 14: brain metastasis | HIST1H3B | D78H | missense_mutation | 75,2% | 880 | 6 | 26.032.057 | C | G | LOH | D | Passenger | PASSENGER/OTHER |  | TRUE | TRUE |  |  | Indeterminate | 100% | 0,0074 | 99% | Clonal | Targeted and amplicon sequencing | enriched for in the metastasis |  |
| 14 | Case 14: brain metastasis | POLH | R105G | missense_mutation | 35,9% | 815 | 6 | 43.555.049 | C | G | No LOH | D | Passenger | PASSENGER/OTHER |  |  |  |  |  | Indeterminate | 100% | 0,9863 | 98% | Clonal | Targeted and amplicon sequencing | private to the metastasis |  |
| 14 | Case 14: brain metastasis | AK9 | K1173N | missense_mutation | 17,2% | 746 | 6 | 109.854.505 | C | G | No LOH | N | Passenger | PASSENGER/OTHER |  |  |  |  |  | Likely passenger | 98% | 0,9060 | 82% | Clonal | Targeted and amplicon sequencing | private to the metastasis |  |
| 14 | Case 14: brain metastasis | AOAH | R630T | missense_mutation | 12,3% | 828 | 7 | 36.552.816 | C | G | No LOH | N | Passenger | PASSENGER/OTHER |  |  |  |  |  | Likely passenger | 95% | 0,8618 | 78% | Clonal | Targeted and amplicon sequencing | private to the metastasis |  |
| 14 | Case 14: brain metastasis | AKAP9 | E102* | nonsense_mutation | 3,6% | 552 | 7 | 91.603.280 | G | T | LOH | A | . | . |  |  | TRUE |  |  | Likely pathogenic | 21% | 0,0000 | 13% | Subclonal | Targeted sequencing | private to the metastasis |  |
| 14 | Case 14: brain metastasis | RELN | E1824Q | missense_mutation | 37,0% | 744 | 7 | 103.202.038 | C | G | LOH | D | Passenger | PASSENGER/OTHER |  |  |  |  | TRUE | Indeterminate | 100% | 0,9768 | 98% | Clonal | Targeted and amplicon sequencing | private to the metastasis |  |
| 14 | Case 14: brain metastasis | BRAF | E703D | missense_mutation | 6,0% | 580 | 7 | 140.439.630 | C | G | LOH | D | Passenger | CANCER | TRUE | TRUE | TRUE |  | TRUE | Likely pathogenic | 35% | 0,0000 | 25% | Subclonal | Targeted and amplicon sequencing | private to the metastasis |  |
| 14 | Case 14: brain metastasis | KMT2C | D4530H | missense_mutation | 73,1% | 869 | 7 | 151.845.424 | C | G | LOH | D | Passenger | PASSENGER/OTHER | TRUE | TRUE | TRUE |  |  | Indeterminate | 100% | 0,2980 | 99% | Clonal | Targeted and amplicon sequencing |  |  |
| 14 | Case 14: brain metastasis | KMT2C | R41S | missense_mutation | 20,0% | 795 | 7 | 152.132.751 | G | T | LOH | D | Passenger | PASSENGER/OTHER | TRUE | TRUE | TRUE |  |  | Indeterminate | 100% | 0,9473 | 90% | Clonal | Targeted and amplicon sequencing | private to the metastasis |  |
| 14 | Case 14: brain metastasis | EPPK1 | M2050I | missense_mutation | 39,6% | 889 | 8 | 144.941.272 | C | T | No LOH | N | Passenger | PASSENGER/OTHER | TRUE |  |  |  |  | Likely passenger | 100% | 0,5756 | 98% | Clonal | Targeted and amplicon sequencing | private to the metastasis |  |
| 14 | Case 14: brain metastasis | CEP164 | S1123C | missense_mutation | 35,3% | 858 | 11 | 117.267.896 | C | G | No LOH | D | Passenger | PASSENGER/OTHER |  |  |  |  |  | Indeterminate | 100% | 0,9886 | 98% | Clonal | Targeted and amplicon sequencing | private to the metastasis |  |
| 14 | Case 14: brain metastasis | KMT2D | E1861K | missense_mutation | 40,4% | 850 | 12 | 49.436.922 | C | T | No LOH | D | Passenger | PASSENGER/OTHER | TRUE | TRUE | TRUE |  |  | Indeterminate | 100% | 0,2835 | 98% | Clonal | Targeted and amplicon sequencing | private to the metastasis |  |
| 14 | Case 14: brain metastasis | BRCA2 | E3343K | missense_mutation | 16,0% | 674 | 13 | 32.972.677 | G | A | No LOH | D | Passenger | PASSENGER/OTHER | TRUE | TRUE | TRUE |  | TRUE | Indeterminate | 92% | 0,8042 | 76% | Clonal | Targeted and amplicon sequencing | private to the metastasis |  |
| 14 | Case 14: brain metastasis | PRKD1 | D893N | missense_mutation | 32,7% | 693 | 14 | 30.046.506 | C | T | No LOH | N | Passenger | PASSENGER/OTHER |  |  |  |  |  | Likely passenger | 100% | 0,9754 | 97% | Clonal | Targeted and amplicon sequencing | enriched for in the metastasis |  |
| 14 | Case 14: brain metastasis | ZFP36L1 | D304N | missense_mutation | 38,9% | 876 | 14 | 69.256.564 | C | T | No LOH | D | Passenger | PASSENGER/OTHER |  |  |  |  |  | Indeterminate | 100% | 0,8099 | 98% | Clonal | Targeted and amplicon sequencing | enriched for in the metastasis |  |
| 14 | Case 14: brain metastasis | AHNAK2 | E201Q | missense_mutation | 18,3% | 872 | 14 | 105.421.344 | C | G | No LOH | N | Passenger | PASSENGER/OTHER |  |  |  |  |  | Likely passenger | 100% | 0,9493 | 87% | Clonal | Targeted and amplicon sequencing | private to the metastasis |  |
| 14 | Case 14: brain metastasis | SRCAP | E2638K | missense_mutation | 4,7% | 904 | 16 | 30.749.273 | G | A | No LOH | N | Passenger | PASSENGER/OTHER |  |  |  |  |  | Likely passenger | 36% | 0,0000 | 26% | Subclonal | Targeted and amplicon sequencing | private to the metastasis |  |
| 14 | Case 14: brain metastasis | TP53 | Q331* | nonsense_mutation | 59,2% | 689 | 17 | 7.576.855 | G | A | LOH | A | . | . | TRUE | TRUE | TRUE | Hotspot | TRUE | Likely pathogenic | 100% | 0,6256 | 99% | Clonal | Targeted and amplicon sequencing | enriched for in the metastasis | Yes |
| 14 | Case 14: brain metastasis | ERBB2 | P1227S | missense_mutation | 8,63% | 46858 | 17 | 37.884.208 | C | T | LOH | D | Passenger | CANCER |  | TRUE | TRUE |  |  | Likely pathogenic | 100% | 0,0000 | 99% | Clonal | Amplicon sequencing |  |  |
| 14 | Case 14: brain metastasis | TOP2A | D526N | missense_mutation | 60,3% | 832 | 17 | 38.563.851 | C | T | LOH | D | Passenger | PASSENGER/OTHER |  |  |  |  |  | Indeterminate | 100% | 0,6935 | 99% | Clonal | Targeted and amplicon sequencing |  | Yes |
| 14 | Case 14: brain metastasis | TOP2A | D524N | missense_mutation | 60,5% | 827 | 17 | 38.563.857 | C | T | LOH | D | Passenger | PASSENGER/OTHER |  |  |  |  |  | Indeterminate | 100% | 0,7050 | 99% | Clonal | Targeted and amplicon sequencing |  | Yes |
| 14 | Case 14: brain metastasis | BRCA1 | S449C | missense_mutation | 11,2% | 463 | 17 | 41.246.202 | G | C | LOH | N | Passenger | PASSENGER/OTHER | TRUE | TRUE | TRUE |  | TRUE | Likely passenger | 42% | 0,0000 | 32% | Subclonal | Targeted and amplicon sequencing | private to the metastasis |  |
| 14 | Case 14: brain metastasis | RPTOR | E163Q | missense_mutation | 79,3% | 911 | 17 | 78.681.779 | G | C | LOH | D | Passenger | PASSENGER/OTHER |  |  |  |  |  | Indeterminate | 100% | 0,7170 | 99% | Clonal | Targeted and amplicon sequencing |  |  |
| 14 | Case 14: brain metastasis | TYK2 | E437K | missense_mutation | 36,2% | 816 | 19 | 10.475.348 | C | T | No LOH | D | Passenger | PASSENGER/OTHER |  |  |  |  |  | Indeterminate | 100% | 0,9852 | 98% | Clonal | Targeted and amplicon sequencing | enriched for in the metastasis |  |
| 14 | Case 14: brain metastasis | CACNA1A | G682R | missense_mutation | 35,4% | 855 | 19 | 13.414.644 | C | G | No LOH | D | Passenger | PASSENGER/OTHER |  |  |  |  |  | Indeterminate | 100% | 0,9884 | 98% | Clonal | Targeted and amplicon sequencing | private to the metastasis |  |
| 14 | Case 14: brain metastasis | POLD1 | E795Q | missense_mutation | 33,9% | 814 | 19 | 50.917.131 | G | C | No LOH | D | Passenger | PASSENGER/OTHER |  |  |  |  |  | Indeterminate | 100% | 0,9865 | 98% | Clonal | Targeted and amplicon sequencing | private to the metastasis |  |
| 14 | Case 14: brain metastasis | CHD6 | L1643H | missense_mutation | 18,2% | 843 | 20 | 40.050.347 | A | T | No LOH | D | Passenger | PASSENGER/OTHER |  |  |  |  |  | Indeterminate | 100% | 0,9444 | 86% | Clonal | Targeted and amplicon sequencing | private to the metastasis |  |
| 14 | Case 14: brain metastasis | NCOA3 | Q1358* | nonsense_mutation | 36,9% | 667 | 20 | 46.281.275 | C | T | No LOH | A | . | . |  |  |  |  |  | Indeterminate | 100% | 0,9782 | 98% | Clonal | Targeted and amplicon sequencing | enriched for in the metastasis |  |
| 14 | Case 14: brain metastasis | RPGR | E709K | missense_mutation | 34,0% | 727 | X | 38.146.127 | C | T | No LOH | N | Passenger | PASSENGER/OTHER |  |  |  |  |  | Likely passenger | 100% | 0,9863 | 97% | Clonal | Targeted and amplicon sequencing | private to the metastasis |  |
| 14 | Case 14: brain metastasis | TENM1 | D477H | missense_mutation | 17,7% | 665 | X | 123.785.914 | C | G | No LOH | N | Passenger | PASSENGER/OTHER |  |  |  |  |  | Likely passenger | 100% | 0,9255 | 83% | Clonal | Targeted and amplicon sequencing | private to the metastasis |  |
| 14 | Case 14: primary breast tumor | CACNA1E | L1575V | missense_mutation | 11,45% | 4890 | 1 | 181.732.575 | C | G | No LOH | D | Passenger | PASSENGER/OTHER |  |  |  |  |  | Indeterminate | 61% | 0,0000 | 56% | Subclonal | Amplicon sequencing | enriched for in the metastasis |  |
| 14 | Case 14: primary breast tumor | APOBEC4 | S25F | missense_mutation | 8,1% | 185 | 1 | 183.617.843 | G | A | No LOH | D | Passenger | PASSENGER/OTHER |  |  |  |  |  | Indeterminate | 44% | 0,0005 | 26% | Subclonal | Targeted and amplicon sequencing |  |  |
| 14 | Case 14: primary breast tumor | SOS1 | D169H | missense_mutation | 15,21% | 4155 | 2 | 39.283.848 | C | G | No LOH | D | Passenger | PASSENGER/OTHER |  |  |  |  |  | Indeterminate | 82% | 0,0000 | 75% | Subclonal | Amplicon sequencing |  |  |
| 14 | Case 14: primary breast tumor | FANCD2 | E14Q | missense_mutation | 12,9% | 179 | 3 | 10.070.381 | G | C | No LOH | N | Passenger | PASSENGER/OTHER |  | TRUE | TRUE |  |  | Likely passenger | 69% | 0,1346 | 46% | Subclonal | Targeted and amplicon sequencing |  |  |
| 14 | Case 14: primary breast tumor | HIST1H3B | E134Q | missense_mutation | 10,3% | 224 | 6 | 26.031.889 | C | G | LOH | D | Passenger | PASSENGER/OTHER |  | TRUE | TRUE |  |  | Indeterminate | 55% | 0,0042 | 37% | Subclonal | Targeted and amplicon sequencing |  |  |
| 14 | Case 14: primary breast tumor | HIST1H3B | D78H | missense_mutation | 12,7% | 776 | 6 | 26.032.057 | C | G | LOH | D | Passenger | PASSENGER/OTHER |  | TRUE | TRUE |  |  | Indeterminate | 68% | 0,0002 | 56% | Subclonal | Targeted and amplicon sequencing | enriched for in the metastasis |  |
| 14 | Case 14: primary breast tumor | KMT2C | D4530H | missense_mutation | 18,4% | 317 | 7 | 151.845.424 | C | G | LOH | D | Passenger | PASSENGER/OTHER | TRUE | TRUE | TRUE |  |  | Indeterminate | 100% | 0,8422 | 82% | Clonal | Targeted and amplicon sequencing |  |  |
| 14 | Case 14: primary breast tumor | CUBN | E1520K | missense_mutation | 13,5% | 156 | 10 | 17.024.620 | C | T | No LOH | D | Passenger | PASSENGER/OTHER |  |  |  |  |  | Indeterminate | 72% | 0,2231 | 48% | Subclonal | Targeted and amplicon sequencing |  |  |
| 14 | Case 14: primary breast tumor | ANK3 | E3429Q | missense_mutation | 27,5% | 218 | 10 | 61.830.354 | C | G | LOH | D | Passenger | CANCER |  |  |  |  |  | Likely pathogenic | 100% | 0,3994 | 88% | Subclonal | Targeted and amplicon sequencing |  |  |
| 14 | Case 14: primary breast tumor | ATM | E2744* | nonsense_mutation | 10,9% | 176 | 11 | 108.206.650 | G | T | No LOH | D | . | . | TRUE | TRUE | TRUE |  | TRUE | Likely pathogenic | 58% | 0,0237 | 37% | Subclonal | Targeted and amplicon sequencing |  |  |
| 14 | Case 14: primary breast tumor | CHD4 | D314Y | missense_mutation | 10,3% | 145 | 12 | 6.709.823 | C | A | No LOH | D | Passenger | PASSENGER/OTHER |  | TRUE |  |  |  | Indeterminate | 56% | 0,0290 | 34% | Subclonal | Targeted and amplicon sequencing |  |  |
| 14 | Case 14: primary breast tumor | PRKD1 | D893N | missense_mutation | 14,9% | 221 | 14 | 30.046.506 | C | T | No LOH | N | Passenger | PASSENGER/OTHER |  |  |  |  |  | Likely passenger | 80% | 0,3439 | 57% | Subclonal | Targeted and amplicon sequencing | enriched for in the metastasis |  |
| 14 | Case 14: primary breast tumor | ZFP36L1 | D304N | missense_mutation | 13,3% | 760 | 14 | 69.256.564 | C | T | No LOH | D | Passenger | PASSENGER/OTHER |  |  |  |  |  | Indeterminate | 71% | 0,0011 | 59% | Subclonal | Targeted and amplicon sequencing | enriched for in the metastasis |  |
| 14 | Case 14: primary breast tumor | SRCAP | H2925Q | missense_mutation | 11,9% | 371 | 16 | 30.750.136 | C | G | No LOH | N | Passenger | PASSENGER/OTHER |  |  |  |  |  | Likely passenger | 64% | 0,0037 | 48% | Subclonal | Targeted and amplicon sequencing |  |  |
| 14 | Case 14: primary breast tumor | TP53 | Q331* | nonsense_mutation | 13,3% | 135 | 17 | 7.576.855 | G | A | No LOH | A | . | . | TRUE | TRUE | TRUE | Hotspot | TRUE | Likely pathogenic | 72% | 0,2364 | 45% | Subclonal | Targeted and amplicon sequencing | enriched for in the metastasis |  |
| 14 | Case 14: primary breast tumor | ERBB2 | P1227S | missense_mutation | 3,7% | 911 | 17 | 37.884.208 | C | T | LOH | D | Passenger | CANCER |  | TRUE | TRUE |  |  | Likely pathogenic | 100% | 0,7072 | 70% | Clonal | Targeted and amplicon sequencing |  |  |
| 14 | Case 14: primary breast tumor | TOP2A | E1226K | missense_mutation | 10,5% | 181 | 17 | 38.552.579 | C | T | No LOH | D | Passenger | PASSENGER/OTHER |  |  |  |  |  | Indeterminate | 67% | 0,1376 | 43% | Subclonal | Targeted and amplicon sequencing |  |  |
| 14 | Case 14: primary breast tumor | TOP2A | D526N | missense_mutation | 15,8% | 152 | 17 | 38.563.851 | C | T | No LOH | D | Passenger | PASSENGER/OTHER |  |  |  |  |  | Indeterminate | 100% | 0,6719 | 64% | Clonal | Targeted sequencing |  |  |
| 14 | Case 14: primary breast tumor | TOP2A | D524N | missense_mutation | 14,2% | 148 | 17 | 38.563.857 | C | T | No LOH | D | Passenger | PASSENGER/OTHER |  |  |  |  |  | Indeterminate | 90% | 0,5606 | 58% | Clonal | Targeted sequencing |  |  |
| 14 | Case 14: primary breast tumor | RPTOR | E163Q | missense_mutation | 15,3% | 536 | 17 | 78.681.779 | G | C | LOH | D | Passenger | PASSENGER/OTHER |  |  |  |  |  | Indeterminate | 82% | 0,2164 | 67% | Subclonal | Targeted and amplicon sequencing |  |  |
| 14 | Case 14: primary breast tumor | TYK2 | E437K | missense_mutation | 10,8% | 716 | 19 | 10.475.348 | C | T | No LOH | D | Passenger | PASSENGER/OTHER |  |  |  |  |  | Indeterminate | 58% | 0,0000 | 46% | Subclonal | Targeted and amplicon sequencing | enriched for in the metastasis |  |
| 14 | Case 14: primary breast tumor | TYK2 | S134L | missense_mutation | 9,8% | 451 | 19 | 10.478.795 | G | A | No LOH | N | Passenger | PASSENGER/OTHER |  |  |  |  |  | Likely passenger | 52% | 0,0000 | 39% | Subclonal | Targeted and amplicon sequencing |  |  |
| 14 | Case 14: primary breast tumor | NCOA3 | Q1358* | nonsense_mutation | 13,2% | 167 | 20 | 46.281.275 | C | T | No LOH | A | . | . |  |  |  |  |  | Indeterminate | 58% | 0,0098 | 38% | Subclonal | Targeted and amplicon sequencing | enriched for in the metastasis |  |
| 14 | Case 14: primary breast tumor | APOBEC3A | L55V | missense_mutation | 16,5% | 297 | 22 | 39.355.680 | C | G | No LOH | N | Passenger | PASSENGER/OTHER |  |  |  |  |  | Likely passenger | 89% | 0,5425 | 67% | Clonal | Targeted and amplicon sequencing |  |  |
| 2 | Case 2: brain metastasis | TP53 | R248W | missense_mutation | 3,8% | 318 | 17 | 7.577.539 | G | A | LOH | A | Driver | CANCER | TRUE | TRUE | TRUE | Hotspot | TRUE | Likely pathogenic | 20% | 0,0000 | 10,98% | Subclonal | Targeted sequencing | private to the metastasis |  |
| 2 | Case 2: primary breast tumor | MACF1 | G3580D | missense_mutation | 8,3% | 411 | 1 | 39.895.535 | G | A | No LOH | D | Passenger | PASSENGER/OTHER |  |  |  |  |  | Indeterminate | 45% | 0,0000 | 32,10% | Subclonal | Targeted sequencing |  |  |
| 6 | Case 6: brain metastasis | ATRX | P711H | missense_mutation | 25,4% | 67 | X | 76.938.616 | G | T | No LOH | D | Passenger | PASSENGER/OTHER | TRUE | TRUE | TRUE |  |  | Indeterminate | 100% | 0,8017 | 64,66% | Clonal | Targeted and amplicon sequencing | enriched for in the metastasis |  |
| 6 | Case 6: primary breast tumor | ATRX | P711H | missense_mutation | 12,3% | 213 | X | 76.938.616 | G | T | No LOH | D | Passenger | PASSENGER/OTHER | TRUE | TRUE | TRUE |  |  | Indeterminate | 46% | 0,0000 | 31,53% | Subclonal | Targeted and amplicon sequencing | enriched for in the metastasis |  |
| Index | Index: brain metastasis # 1 | UBR4 | K1150T | missense_mutation | 6,4% | 867 | 1 | 19.499.430 | T | G | No LOH | D | Passenger | PASSENGER/OTHER |  |  |  |  |  | Indeterminate | 27% | 0,0000 | 20,05% | Subclonal | Targeted and amplicon sequencing | private to the metastasis |  |
| Index | Index: brain metastasis # 1 | CACNA1E | L2058V | missense_mutation | 5,2% | 690 | 1 | 181.764.144 | T | G | No LOH | D | Passenger | PASSENGER/OTHER |  |  |  |  |  | Indeterminate | 32% | 0,0000 | 23,00% | Subclonal | Targeted and amplicon sequencing | private to the metastasis |  |
| Index | Index: brain metastasis # 1 | ATR | C2150W | missense_mutation | 3,0% | 862 | 3 | 142.188.281 | A | C | No LOH | D | Driver | PASSENGER/OTHER | TRUE | TRUE |  |  | TRUE | Likely pathogenic | 16% | 0,0000 | 10,20% | Subclonal | Targeted and amplicon sequencing | private to the metastasis |  |
| Index | Index: brain metastasis # 1 | RELN | L1854R | missense_mutation | 11,7% | 835 | 7 | 103.198.465 | A | C | LOH | D | Passenger | PASSENGER/OTHER |  |  |  |  | TRUE | Indeterminate | 61% | 0,0000 | 49,95% | Subclonal | Targeted and amplicon sequencing | private to the metastasis |  |
| Index | Index: brain metastasis # 1 | RELN | E701G | missense_mutation | 3,1% | 886 | 7 | 103.276.883 | T | C | LOH | D | Passenger | PASSENGER/OTHER |  |  |  |  | TRUE | Indeterminate | 16% | 0,0000 | 10,37% | Subclonal | Targeted and amplicon sequencing | private to the metastasis |  |
| Index | Index: brain metastasis # 1 | BRAF | L232R | missense_mutation | 8,1% | 886 | 7 | 140.507.776 | A | C | No LOH | D | Passenger | PASSENGER/OTHER | TRUE | TRUE | TRUE |  | TRUE | Indeterminate | 67% | 0,0009 | 52,74% | Subclonal | Targeted sequencing | private to the metastasis |  |
| Index | Index: brain metastasis # 1 | PAXIP1 | L537F | missense_mutation | 20,6% | 755 | 7 | 154.760.302 | G | A | No LOH | N | Passenger | PASSENGER/OTHER |  |  |  |  |  | Likely passenger | 100% | 0,6486 | 95,21% | Clonal | Targeted sequencing | enriched for in the metastasis |  |
| Index | Index: brain metastasis # 1 | NBN | E510A | missense_mutation | 10,3% | 907 | 8 | 90.965.788 | T | G | No LOH | N | Passenger | PASSENGER/OTHER |  | TRUE | TRUE |  |  | Likely passenger | 53% | 0,0000 | 43,27% | Subclonal | Targeted and amplicon sequencing | private to the metastasis |  |
| Index | Index: brain metastasis # 1 | FGFR2 | D759H | missense_mutation | 43,7% | 468 | 10 | 123.243.241 | C | G | LOH | D | Passenger | CANCER | TRUE | TRUE | TRUE |  |  | Likely pathogenic | 96% | 0,7949 | 85,13% | Clonal | Targeted and amplicon sequencing | private to the metastasis |  |
| Index | Index: brain metastasis # 1 | BRCA2 | L997V | missense_mutation | 3,3% | 879 | 13 | 32.911.481 | T | G | LOH | N | Passenger | PASSENGER/OTHER | TRUE | TRUE | TRUE |  | TRUE | Likely passenger | 14% | 0,0000 | 9,15% | Subclonal | Targeted and amplicon sequencing | private to the metastasis |  |
| Index | Index: brain metastasis # 1 | AHNAK2 | L4986R | missense_mutation | 9,6% | 920 | 14 | 105.406.831 | A | C | No LOH | N | Passenger | PASSENGER/OTHER |  |  |  |  |  | Likely passenger | 69% | 0,0007 | 55,76% | Subclonal | Targeted and amplicon sequencing | private to the metastasis |  |
| Index | Index: brain metastasis # 1 | ZFHX3 | L357R | missense_mutation | 3,1% | 691 | 16 | 72.992.975 | A | C | No LOH | D | Passenger | PASSENGER/OTHER |  | TRUE |  |  |  | Indeterminate | 10% | 0,0000 | 5,87% | Subclonal | Targeted and amplicon sequencing | private to the metastasis |  |
| Index | Index: brain metastasis # 1 | MAP2K4 | S303R | missense_mutation | 3,0% | 837 | 17 | 12.028.671 | A | C | LOH | D | Passenger | PASSENGER/OTHER | TRUE | TRUE | TRUE |  |  | Indeterminate | 13% | 0,0000 | 8,04% | Subclonal | Targeted and amplicon sequencing | private to the metastasis |  |
| Index | Index: brain metastasis # 1 | TOP2A | V983G | missense_mutation | 16,4% | 860 | 17 | 38.556.513 | A | C | No LOH | D | Passenger | PASSENGER/OTHER |  |  |  |  |  | Indeterminate | 69% | 0,0000 | 58,36% | Subclonal | Targeted and amplicon sequencing | private to the metastasis |  |
| Index | Index: brain metastasis # 1 | CACNA1A | K1365T | missense_mutation | 12,3% | 861 | 19 | 13.373.555 | T | G | No LOH | D | Passenger | PASSENGER/OTHER |  |  |  |  |  | Indeterminate | 64% | 0,0000 | 52,81% | Subclonal | Targeted and amplicon sequencing | private to the metastasis |  |
| Index | Index: brain metastasis # 1 | CHEK2 | K298T | missense_mutation | 6,2% | 941 | 22 | 29.107.925 | T | G | No LOH | D | Passenger | PASSENGER/OTHER | TRUE | TRUE | TRUE |  |  | Indeterminate | 69% | 0,0115 | 53,19% | Subclonal | Targeted sequencing | private to the metastasis |  |
| Index | Index: brain metastasis # 2 | PIK3CA | K111T | missense_mutation | 30,9% | 898 | 3 | 178.916.945 | A | C | No LOH | D | Driver | CANCER | TRUE | TRUE | TRUE | Hotspot |  | Likely pathogenic | 100% | 0,9200 | 89,64% | Clonal | Targeted and amplicon sequencing | private to the metastasis |  |
| Index | Index: brain metastasis # 2 | INPP4B | Q498H | missense_mutation | 16,0% | 921 | 4 | 143.081.580 | T | G | No LOH | D | Passenger | PASSENGER/OTHER |  |  |  |  |  | Indeterminate | 53% | 0,0000 | 44,72% | Subclonal | Targeted and amplicon sequencing | private to the metastasis |  |
| Index | Index: brain metastasis # 2 | FANCE | L369R | missense_mutation | 11,5% | 739 | 6 | 35.426.210 | T | G | No LOH | D | Passenger | PASSENGER/OTHER |  | TRUE | TRUE |  |  | Indeterminate | 49% | 0,0000 | 39,85% | Subclonal | Targeted and amplicon sequencing | private to the metastasis |  |
| Index | Index: brain metastasis # 2 | MDN1 | S585fs | frameshift_deletion | 18,6% | 1693 | 6 | 90.486.385 | AG | A | No LOH | . | . | . |  |  |  |  |  | Indeterminate | 80% | 0,0001 | 71,70% | Subclonal | Targeted and amplicon sequencing | private to the metastasis |  |
| Index | Index: brain metastasis # 2 | HECW1 | D448A | missense_mutation | 6,1% | 936 | 7 | 43.484.114 | A | C | No LOH | N | Passenger | PASSENGER/OTHER |  |  |  |  |  | Likely passenger | 26% | 0,0000 | 19,80% | Subclonal | Targeted and amplicon sequencing | private to the metastasis |  |
| Index | Index: brain metastasis # 2 | RELN | N290T | missense_mutation | 25,4% | 833 | 7 | 103.341.390 | T | G | LOH | D | Passenger | PASSENGER/OTHER |  |  |  |  | TRUE | Indeterminate | 58% | 0,0000 | 51,30% | Subclonal | Targeted and amplicon sequencing | private to the metastasis |  |
| Index | Index: brain metastasis # 2 | PAXIP1 | L537F | missense_mutation | 25,8% | 841 | 7 | 154.760.302 | G | A | No LOH | N | Passenger | PASSENGER/OTHER |  |  |  |  |  | Likely passenger | 100% | 0,9338 | 91,60% | Clonal | Targeted sequencing | enriched for in the metastasis |  |
| Index | Index: brain metastasis # 2 | FGFR2 | D759H | missense_mutation | 47,3% | 800 | 10 | 123.243.241 | C | G | LOH | D | Passenger | CANCER | TRUE | TRUE | TRUE |  |  | Likely pathogenic | 100% | 0,9482 | 94,92% | Clonal | Targeted and amplicon sequencing | private to the metastasis |  |
| Index | Index: brain metastasis # 3 | MACF1 | L617V | missense_mutation | 5,4% | 552 | 1 | 39.757.630 | T | G | No LOH | D | Passenger | PASSENGER/OTHER |  |  |  |  |  | Indeterminate | 13% | 0,0000 | 8,69% | Subclonal | Targeted and amplicon sequencing | private to the metastasis |  |
| Index | Index: brain metastasis # 3 | PAXIP1 | L537F | missense_mutation | 22,3% | 676 | 7 | 154.760.302 | G | A | No LOH | N | Passenger | PASSENGER/OTHER |  |  |  |  |  | Likely passenger | 100% | 0,8299 | 91,40% | Clonal | Targeted sequencing | enriched for in the metastasis |  |
| Index | Index: brain metastasis # 3 | ZFHX4 | L686P | missense_mutation | 2,6% | 884 | 8 | 77.618.380 | T | C | No LOH | D | Passenger | PASSENGER/OTHER |  |  |  |  |  | Indeterminate | 11% | 0,0000 | 7,16% | Subclonal | Targeted and amplicon sequencing | private to the metastasis |  |
| Index | Index: brain metastasis # 3 | EPPK1 | T568P | missense_mutation | 4,6% | 796 | 8 | 144.945.720 | T | G | LOH | N | Passenger | PASSENGER/OTHER | TRUE |  |  |  |  | Likely passenger | 16% | 0,0000 | 11,07% | Subclonal | Targeted sequencing | private to the metastasis |  |
| Index | Index: brain metastasis # 3 | FGFR2 | D759H | missense_mutation | 44,6% | 524 | 10 | 123.243.241 | C | G | LOH | D | Passenger | CANCER | TRUE | TRUE | TRUE |  |  | Likely pathogenic | 100% | 0,9486 | 92,31% | Clonal | Targeted and amplicon sequencing | private to the metastasis |  |
| Index | Index: primary breast tumor | PAXIP1 | L537F | missense_mutation | 17,5% | 155 | 7 | 154.760.302 | G | A | No LOH | N | Passenger | PASSENGER/OTHER |  |  |  |  |  | Likely passenger | 60% | 0,0101 | 41,98% | Subclonal | Targeted sequencing | enriched for in the metastasis |  |
| Index | Index: primary breast tumor | ERCC2 | R196Q | missense_mutation | 27,8% | 79 | 19 | 45.868.103 | C | T | No LOH | D | Passenger | PASSENGER/OTHER | TRUE | TRUE | TRUE |  |  | Indeterminate | 96% | 0,7206 | 62,54% | Clonal | Targeted sequencing |  |  |
| Index | Index: brain metastasis # 1 | TP53 | X261_splice | splice | 94,5% | 1055 | 17 | 7.577.498 | C | A | LOH | D | . | . | TRUE | TRUE | TRUE |  | TRUE | Likely pathogenic | 100% | 0,0000 | 99,02% | Clonal | Targeted sequencing |  |  |
| Index | Index: brain metastasis # 2 | TP53 | X261_splice | splice | 96,0% | 973 | 17 | 7.577.498 | C | A | LOH | D | . | . | TRUE | TRUE | TRUE |  | TRUE | Likely pathogenic | 100% | 0,9612 | 99,02% | Clonal | Targeted sequencing |  |  |
| Index | Index: brain metastasis # 3 | TP53 | X261_splice | splice | 89,3% | 580 | 17 | 7.577.498 | C | A | LOH | D | . | . | TRUE | TRUE | TRUE |  | TRUE | Likely pathogenic | 100% | 0,0162 | 99,02% | Clonal | Targeted sequencing |  |  |
| Index | Index: primary breast tumor | TP53 | X261_splice | splice | 75,2% | 125 | 17 | 7.577.498 | C | A | LOH | D | . | . | TRUE | TRUE | TRUE |  | TRUE | Likely pathogenic | 100% | 0,9468 | 95,12% | Clonal | Targeted sequencing |  |  |
