# Supplementary material for: Genetic heterogeneity and actionable mutations in HER2-positive primary breast cancers and their brain metastases
Source: Oncotarget. 2018 Apr 17;9(29):20617–30. doi: 10.18632/oncotarget.25041 (PMC5945519; doi:10.18632/oncotarget.25041)
Supplement: Supplementary file 4 [file oncotarget-09-20617-s004.doc]

**Supplementary Table 5: List of potential clinical actionable genes according to the http://dgidb.genome.wustl.edu**

| **Gene** | **Gene Description** | **Source(s)** |
| --- | --- | --- |
| APC | adenomatous polyposis coli | MskImpact CarisMolecularIntelligence FoundationOneGenes |
| TP53 | tumor protein p53 | MskImpact CarisMolecularIntelligence FoundationOneGenes |
| TIMM8B | translocase of inner mitochondrial membrane 8 homolog B (yeast) | MskImpact |
| IFNGR1 | interferon gamma receptor 1 | MskImpact |
| SDHB | succinate dehydrogenase complex, subunit B, iron sulfur (Ip) | MskImpact |
| EPHA3 | EPH receptor A3 | MskImpact FoundationOneGenes |
| RPS6KB2 | ribosomal protein S6 kinase, 70kDa, polypeptide 2 | MskImpact |
| TSC2 | tuberous sclerosis 2 | MskImpact FoundationOneGenes |
| ESR1 | estrogen receptor 1 | MskImpact FoundationOneGenes |
| DOT1L | DOT1-like, histone H3 methyltransferase (S. cerevisiae) | MskImpact FoundationOneGenes |
| TOP1 | topoisomerase (DNA) I | MskImpact FoundationOneGenes |
| DNMT3A | DNA (cytosine-5-)-methyltransferase 3 alpha | MskImpact FoundationOneGenes |
| CBL | Cbl proto-oncogene, E3 ubiquitin protein ligase | MskImpact FoundationOneGenes |
| JAK1 | Janus kinase 1 | MskImpact |
| AR | androgen receptor | MskImpact CarisMolecularIntelligence FoundationOneGenes |
| EP300 | E1A binding protein p300 | MskImpact FoundationOneGenes |
| SRC | v-src sarcoma (Schmidt-Ruppin A-2) viral oncogene homolog (avian) | MskImpact FoundationOneGenes |
| CDKN1A | cyclin-dependent kinase inhibitor 1A (p21, Cip1) | MskImpact |
| TBX3 | T-box 3 | MskImpact |
| FIP1L1 | FIP1 like 1 (S. cerevisiae) | MskImpact |
| MYCL | /genes/MYCL | MskImpact |
| MUTYH | mutY homolog (E. coli) | MskImpact FoundationOneGenes |
| PIK3CA | phosphatidylinositol-4,5-bisphosphate 3-kinase, catalytic subunit alph | MskImpact CarisMolecularIntelligence FoundationOneGenes |
| ABL1 | c-abl oncogene 1, non-receptor tyrosine kinase | MskImpact CarisMolecularIntelligence FoundationOneGenes |
| CCNE1 | cyclin E1 | MskImpact FoundationOneGenes |
| MAP2K1 | mitogen-activated protein kinase kinase 1 | MskImpact FoundationOneGenes |
| PTPRS | protein tyrosine phosphatase, receptor type, S | MskImpact |
| RFWD2 | ring finger and WD repeat domain 2, E3 ubiquitin protein ligase | MskImpact |
| CDH1 | cadherin 1, type 1, E-cadherin (epithelial) | MskImpact CarisMolecularIntelligence FoundationOneGenes |
| POLE | polymerase (DNA directed), epsilon, catalytic subunit | MskImpact |
| EPHB1 | EPH receptor B1 | MskImpact FoundationOneGenes |
| RAD51D | RAD51 homolog D (S. cerevisiae) | MskImpact |
| IGF2 | insulin-like growth factor 2 (somatomedin A) | MskImpact |
| E2F3 | E2F transcription factor 3 | MskImpact |
| ALK | anaplastic lymphoma receptor tyrosine kinase | MskImpact CarisMolecularIntelligence FoundationOneGenes |
| FOXL2NB | /genes/FOXL2NB | MskImpact |
| LATS2 | LATS, large tumor suppressor, homolog 2 (Drosophila) | MskImpact |
| ERBB4 | v-erb-a erythroblastic leukemia viral oncogene homolog 4 (avian) | MskImpact CarisMolecularIntelligence FoundationOneGenes |
| BCOR | BCL6 corepressor | MskImpact FoundationOneGenes |
| NKX3-1 | NK3 homeobox 1 | MskImpact |
| FH | fumarate hydratase | MskImpact |
| KIT | v-kit Hardy-Zuckerman 4 feline sarcoma viral oncogene homolog | MskImpact CarisMolecularIntelligence FoundationOneGenes |
| BCL2 | B-cell CLL/lymphoma 2 | MskImpact FoundationOneGenes |
| FGF19 | fibroblast growth factor 19 | MskImpact FoundationOneGenes |
| CARD11 | caspase recruitment domain family, member 11 | MskImpact FoundationOneGenes |
| KMT2C | /genes/KMT2C | MskImpact |
| SMARCD1 | SWI/SNF related, matrix associated, actin dependent regulator of chr | MskImpact |
| FANCA | Fanconi anemia, complementation group A | MskImpact FoundationOneGenes |
| GSK3B | glycogen synthase kinase 3 beta | MskImpact FoundationOneGenes |
| ASXL1 | additional sex combs like 1 (Drosophila) | MskImpact FoundationOneGenes |
| FLT3 | fms-related tyrosine kinase 3 | MskImpact CarisMolecularIntelligence FoundationOneGenes |
| PIK3CG | phosphatidylinositol-4,5-bisphosphate 3-kinase, catalytic subunit gam | MskImpact FoundationOneGenes |
| NRAS | neuroblastoma RAS viral (v-ras) oncogene homolog | MskImpact CarisMolecularIntelligence FoundationOneGenes |
| RAD51B | RAD51 homolog B (S. cerevisiae) | MskImpact |
| ARID1B | AT rich interactive domain 1B (SWI1-like) | MskImpact |
| SF3B1 | splicing factor 3b, subunit 1, 155kDa | MskImpact FoundationOneGenes |
| BAP1 | BRCA1 associated protein-1 (ubiquitin carboxy-terminal hydrolase) | MskImpact FoundationOneGenes |
| BCL2L11 | BCL2-like 11 (apoptosis facilitator) | MskImpact |
| IGF1R | insulin-like growth factor 1 receptor | MskImpact FoundationOneGenes |
| SMAD4 | SMAD family member 4 | MskImpact CarisMolecularIntelligence FoundationOneGenes |
| RPTOR | regulatory associated protein of MTOR, complex 1 | MskImpact FoundationOneGenes |
| RET | ret proto-oncogene | MskImpact CarisMolecularIntelligence FoundationOneGenes |
| AXL | AXL receptor tyrosine kinase | MskImpact FoundationOneGenes |
| IRF4 | interferon regulatory factor 4 | MskImpact FoundationOneGenes |
| PAK7 | p21 protein (Cdc42/Rac)-activated kinase 7 | MskImpact |
| PIK3R3 | phosphoinositide-3-kinase, regulatory subunit 3 (gamma) | MskImpact |
| INPP4A | inositol polyphosphate-4-phosphatase, type I, 107kDa | MskImpact |
| AKT3 | v-akt murine thymoma viral oncogene homolog 3 (protein kinase B, g | MskImpact FoundationOneGenes |
| AXIN2 | axin 2 | MskImpact |
| TSHR | thyroid stimulating hormone receptor | MskImpact FoundationOneGenes |
| BRIP1 | BRCA1 interacting protein C-terminal helicase 1 | MskImpact FoundationOneGenes |
| MAP3K1 | mitogen-activated protein kinase kinase kinase 1, E3 ubiquitin protein | MskImpact FoundationOneGenes |
| KMT2D | /genes/KMT2D | MskImpact |
| NOTCH1 | notch 1 | MskImpact CarisMolecularIntelligence FoundationOneGenes |
| MYOD1 | myogenic differentiation 1 | MskImpact |
| MPL | myeloproliferative leukemia virus oncogene | MskImpact CarisMolecularIntelligence FoundationOneGenes |
| TSC1 | tuberous sclerosis 1 | MskImpact FoundationOneGenes |
| SMAD2 | SMAD family member 2 | MskImpact FoundationOneGenes |
| CTNNB1 | catenin (cadherin-associated protein), beta 1, 88kDa | MskImpact CarisMolecularIntelligence FoundationOneGenes |
| PIK3CD | phosphatidylinositol-4,5-bisphosphate 3-kinase, catalytic subunit delta | MskImpact |
| PIK3CB | phosphatidylinositol-4,5-bisphosphate 3-kinase, catalytic subunit beta | MskImpact |
| IGF1 | insulin-like growth factor 1 (somatomedin C) | MskImpact |
| PMAIP1 | phorbol-12-myristate-13-acetate-induced protein 1 | MskImpact |
| RARA | retinoic acid receptor, alpha | MskImpact FoundationOneGenes |
| TGFBR2 | transforming growth factor, beta receptor II (70/80kDa) | MskImpact FoundationOneGenes |
| HNF1A | HNF1 homeobox A | MskImpact CarisMolecularIntelligence |
| JUN | jun proto-oncogene | MskImpact FoundationOneGenes |
| MET | met proto-oncogene (hepatocyte growth factor receptor) | MskImpact CarisMolecularIntelligence FoundationOneGenes |
| ARID2 | AT rich interactive domain 2 (ARID, RFX-like) | MskImpact FoundationOneGenes |
| MEN1 | multiple endocrine neoplasia I | MskImpact FoundationOneGenes |
| FGFR4 | fibroblast growth factor receptor 4 | MskImpact |
| TMPRSS2 | transmembrane protease, serine 2 | MskImpact FoundationOneGenes |
| PBRM1 | polybromo 1 | MskImpact FoundationOneGenes |
| BRCA2 | breast cancer 2, early onset | MskImpact CarisMolecularIntelligence FoundationOneGenes |
| GNAS | GNAS complex locus | MskImpact CarisMolecularIntelligence FoundationOneGenes |
| GATA2 | GATA binding protein 2 | MskImpact FoundationOneGenes |
| AURKA | aurora kinase A | MskImpact FoundationOneGenes |
| TOP2A | topoisomerase (DNA) II alpha 170kDa | MskImpact CarisMolecularIntelligence |
| HIST1H2BD | histone cluster 1, H2bd | MskImpact |
| AXIN1 | axin 1 | MskImpact |
| CDKN1B | cyclin-dependent kinase inhibitor 1B (p27, Kip1) | MskImpact FoundationOneGenes |
| TP63 | tumor protein p63 | MskImpact |

| MYCN | v-myc myelocytomatosis viral related oncogene, neuroblastoma deriv | MskImpact | FoundationOneGenes |
| --- | --- | --- | --- |
| IDH1 | isocitrate dehydrogenase 1 (NADP ), soluble | MskImpact | CarisMolecularIntelligence FoundationOneGenes |
| STK11 | serine/threonine kinase 11 | MskImpact | CarisMolecularIntelligence FoundationOneGenes |
| GNAQ | guanine nucleotide binding protein (G protein), q polypeptide | MskImpact | CarisMolecularIntelligence FoundationOneGenes |
| PIK3R1 | phosphoinositide-3-kinase, regulatory subunit 1 (alpha) | MskImpact FoundationOneGenes | |
| PARP1 | poly (ADP-ribose) polymerase 1 | MskImpact |  |
| SMARCB1 | SWI/SNF related, matrix associated, actin dependent regulator of chr | MskImpact CarisMolecularIntelligence FoundationOneGenes | |
| RUNX1 | runt-related transcription factor 1 | MskImpact FoundationOneGenes | |
| RNF43 | ring finger protein 43 | MskImpact FoundationOneGenes | |
| ERG | v-ets erythroblastosis virus E26 oncogene homolog (avian) | MskImpact FoundationOneGenes | |
| CRLF2 | cytokine receptor-like factor 2 | MskImpact FoundationOneGenes | |
| CDKN2C | cyclin-dependent kinase inhibitor 2C (p18, inhibits CDK4) | MskImpact FoundationOneGenes | |
| MAP2K4 | mitogen-activated protein kinase kinase 4 | MskImpact FoundationOneGenes | |
| HIST1H3B | histone cluster 1, H3b | MskImpact |  |
| MSH2 | mutS homolog 2, colon cancer, nonpolyposis type 1 (E. coli) | MskImpact FoundationOneGenes | |
| NF1 | neurofibromin 1 | MskImpact FoundationOneGenes | |
| BBC3 | BCL2 binding component 3 | MskImpact |  |
| EPCAM | epithelial cell adhesion molecule | MskImpact |  |
| CSF1R | colony stimulating factor 1 receptor | MskImpact CarisMolecularIntelligence FoundationOneGenes | |
| ERBB3 | v-erb-b2 erythroblastic leukemia viral oncogene homolog 3 (avian) | MskImpact FoundationOneGenes | |
| FGFR3 | fibroblast growth factor receptor 3 | MskImpact FoundationOneGenes | |
| RAD51 | RAD51 homolog (S. cerevisiae) | MskImpact FoundationOneGenes | |
| ATM | ataxia telangiectasia mutated | MskImpact CarisMolecularIntelligence FoundationOneGenes | |
| NSD1 | nuclear receptor binding SET domain protein 1 | MskImpact |  |
| MYC | v-myc myelocytomatosis viral oncogene homolog (avian) | MskImpact FoundationOneGenes | |
| RAC1 | ras-related C3 botulinum toxin substrate 1 (rho family, small GTP bind | MskImpact |  |
| ICOSLG | inducible T-cell co-stimulator ligand | MskImpact |  |
| RAD50 | RAD50 homolog (S. cerevisiae) | MskImpact FoundationOneGenes | |
| ASXL2 | additional sex combs like 2 (Drosophila) | MskImpact |  |
| TERT | telomerase reverse transcriptase | MskImpact |  |
| GATA3 | GATA binding protein 3 | MskImpact FoundationOneGenes | |
| YES1 | v-yes-1 Yamaguchi sarcoma viral oncogene homolog 1 | MskImpact |  |
| PDPK1 | 3-phosphoinositide dependent protein kinase-1 | MskImpact FoundationOneGenes | |
| BLM | Bloom syndrome, RecQ helicase-like | MskImpact FoundationOneGenes | |
| SDHAF2 | succinate dehydrogenase complex assembly factor 2 | MskImpact |  |
| HIST1H1C | histone cluster 1, H1c | MskImpact |  |
| GRIN2A | glutamate receptor, ionotropic, N-methyl D-aspartate 2A | MskImpact FoundationOneGenes | |
| STK40 | serine/threonine kinase 40 | MskImpact |  |
| KDR | kinase insert domain receptor (a type III receptor tyrosine kinase) | MskImpact FoundationOneGenes | |
| SYK | spleen tyrosine kinase | MskImpact |  |
| EZH2 | enhancer of zeste homolog 2 (Drosophila) | MskImpact FoundationOneGenes | |
| FBXW7 | F-box and WD repeat domain containing 7, E3 ubiquitin protein ligase | MskImpact CarisMolecularIntelligence FoundationOneGenes | |
| KDM6A | lysine (K)-specific demethylase 6A | MskImpact FoundationOneGenes | |
| GNA11 | guanine nucleotide binding protein (G protein), alpha 11 (Gq class) | MskImpact CarisMolecularIntelligence FoundationOneGenes | |
| CHEK2 | checkpoint kinase 2 | MskImpact FoundationOneGenes | |
| CTLA4 | cytotoxic T-lymphocyte-associated protein 4 | MskImpact |  |
| RYBP | RING1 and YY1 binding protein | MskImpact |  |
| LATS1 | LATS, large tumor suppressor, homolog 1 (Drosophila) | MskImpact |  |
| MLH1 | mutL homolog 1, colon cancer, nonpolyposis type 2 (E. coli) | MskImpact FoundationOneGenes | |
| TNFRSF14 | tumor necrosis factor receptor superfamily, member 14 | MskImpact FoundationOneGenes | |
| MAX | MYC associated factor X | MskImpact |  |
| LMO1 | LIM domain only 1 (rhombotin 1) | MskImpact |  |
| SDHA | succinate dehydrogenase complex, subunit A, flavoprotein (Fp) | MskImpact |  |
| CD79B | CD79b molecule, immunoglobulin-associated beta | MskImpact FoundationOneGenes | |
| DCUN1D1 | DCN1, defective in cullin neddylation 1, domain containing 1 (S. cere | MskImpact |  |
| HIST1H3C | histone cluster 1, H3c | MskImpact |  |
| FGFR2 | fibroblast growth factor receptor 2 | MskImpact CarisMolecularIntelligence FoundationOneGenes | |
| CD274 | CD274 molecule | MskImpact |  |
| RIT1 | Ras-like without CAAX 1 | MskImpact |  |
| PDGFRB | platelet-derived growth factor receptor, beta polypeptide | MskImpact FoundationOneGenes | |
| SHQ1 | SHQ1 homolog (S. cerevisiae) | MskImpact |  |
| INSR | insulin receptor | MskImpact |  |
| RB1 | retinoblastoma 1 | MskImpact CarisMolecularIntelligence FoundationOneGenes | |
| ARAF | v-raf murine sarcoma 3611 viral oncogene homolog | MskImpact FoundationOneGenes | |
| MED12 | mediator complex subunit 12 | MskImpact FoundationOneGenes | |
| SOX2-OT | SOX2 overlapping transcript (non-protein coding) | MskImpact |  |
| SOX9-AS1 | /genes/SOX9-AS1 | MskImpact |  |
| BCL2L1 | BCL2-like 1 | MskImpact |  |
| BCL6 | B-cell CLL/lymphoma 6 | MskImpact FoundationOneGenes | |
| NOTCH2 | notch 2 | MskImpact FoundationOneGenes | |
| YAP1 | Yes-associated protein 1 | MskImpact |  |
| CIC | capicua homolog (Drosophila) | MskImpact FoundationOneGenes | |
| EGFL7 | EGF-like-domain, multiple 7 | MskImpact |  |
| NF2 | neurofibromin 2 (merlin) | MskImpact FoundationOneGenes | |
| ERBB2 | v-erb-b2 erythroblastic leukemia viral oncogene homolog 2, neuro/glio | MskImpact CarisMolecularIntelligence FoundationOneGenes | |
| EPHA5 | EPH receptor A5 | MskImpact FoundationOneGenes | |
| PTEN | phosphatase and tensin homolog | MskImpact CarisMolecularIntelligence FoundationOneGenes | |
| FOXA1 | forkhead box A1 | MskImpact |  |
| NPM1 | nucleophosmin (nucleolar phosphoprotein B23, numatrin) | MskImpact CarisMolecularIntelligence FoundationOneGenes | |
| CDK6 | cyclin-dependent kinase 6 | MskImpact FoundationOneGenes | |
| TET1 | tet methylcytosine dioxygenase 1 | MskImpact |  |
| DDR2 | discoidin domain receptor tyrosine kinase 2 | MskImpact FoundationOneGenes | |
| MRE11A | MRE11 meiotic recombination 11 homolog A (S. cerevisiae) | MskImpact FoundationOneGenes | |
| CDKN2A | cyclin-dependent kinase inhibitor 2A | MskImpact FoundationOneGenes | |
| DIS3 | DIS3 mitotic control homolog (S. cerevisiae) | MskImpact |  |
| SMARCA4 | SWI/SNF related, matrix associated, actin dependent regulator of chr | MskImpact FoundationOneGenes | |
| MSH6 | mutS homolog 6 (E. coli) | MskImpact FoundationOneGenes | |
| PRKAR1A | protein kinase, cAMP-dependent, regulatory, type I, alpha | MskImpact FoundationOneGenes | |
| MDM2 | Mdm2, p53 E3 ubiquitin protein ligase homolog (mouse) | MskImpact FoundationOneGenes | |
| HGF | hepatocyte growth factor (hepapoietin A; scatter factor) | MskImpact FoundationOneGenes | |
| NOTCH4 | notch 4 | MskImpact |  |
| CCND1 | cyclin D1 | MskImpact FoundationOneGenes | |
| KRAS | v-Ki-ras2 Kirsten rat sarcoma viral oncogene homolog | MskImpact CarisMolecularIntelligence FoundationOneGenes | |
| RASA1 | RAS p21 protein activator (GTPase activating protein) 1 | MskImpact |  |
| IRS1 | insulin receptor substrate 1 | MskImpact |  |
| HRAS | v-Ha-ras Harvey rat sarcoma viral oncogene homolog | MskImpact CarisMolecularIntelligence FoundationOneGenes | |
| SUZ12 | suppressor of zeste 12 homolog (Drosophila) | MskImpact |  |
| ERCC3 | excision repair cross-complementing rodent repair deficiency, comple | MskImpact |  |
| ROS1 | c-ros oncogene 1 , receptor tyrosine kinase | MskImpact CarisMolecularIntelligence FoundationOneGenes | |
| AKT2 | v-akt murine thymoma viral oncogene homolog 2 | MskImpact FoundationOneGenes | |
| RAD51C | RAD51 homolog C (S. cerevisiae) | MskImpact |  |
| CCND3 | cyclin D3 | MskImpact FoundationOneGenes | |
| FAM46C | family with sequence similarity 46, member C | MskImpact FoundationOneGenes | |
| EED | embryonic ectoderm development | MskImpact |  |

| BMPR1A | bone morphogenetic protein receptor, type IA | MskImpact |
| --- | --- | --- |
| NFE2L2 | nuclear factor (erythroid-derived 2)-like 2 | MskImpact FoundationOneGenes |
| CREBBP | CREB binding protein | MskImpact FoundationOneGenes |
| EGFR | epidermal growth factor receptor | MskImpact CarisMolecularIntelligence FoundationOneGenes |
| VHL | von Hippel-Lindau tumor suppressor, E3 ubiquitin protein ligase | MskImpact CarisMolecularIntelligence FoundationOneGenes |
| MAP2K2 | mitogen-activated protein kinase kinase 2 | MskImpact FoundationOneGenes |
| FGF4 | fibroblast growth factor 4 | MskImpact FoundationOneGenes |
| BRD4 | bromodomain containing 4 | MskImpact |
| VTCN1 | V-set domain containing T cell activation inhibitor 1 | MskImpact |
| SOX17 | SRY (sex determining region Y)-box 17 | MskImpact |
| HIST1H4B | histone cluster 1, H4b | MskImpact |
| TNFAIP3 | tumor necrosis factor, alpha-induced protein 3 | MskImpact FoundationOneGenes |
| SUFU | suppressor of fused homolog (Drosophila) | MskImpact FoundationOneGenes |
| GATA1 | GATA binding protein 1 (globin transcription factor 1) | MskImpact FoundationOneGenes |
| PALB2 | partner and localizer of BRCA2 | MskImpact FoundationOneGenes |
| PMS1 | PMS1 postmeiotic segregation increased 1 (S. cerevisiae) | MskImpact |
| MYD88 | myeloid differentiation primary response gene (88) | MskImpact FoundationOneGenes |
| H3F3C | H3 histone, family 3C | MskImpact |
| MAP3K13 | mitogen-activated protein kinase kinase kinase 13 | MskImpact |
| IKBKE | inhibitor of kappa light polypeptide gene enhancer in B-cells, kinase e | MskImpact FoundationOneGenes |
| MITF | microphthalmia-associated transcription factor | MskImpact FoundationOneGenes |
| AKT1 | v-akt murine thymoma viral oncogene homolog 1 | MskImpact CarisMolecularIntelligence FoundationOneGenes |
| RICTOR | RPTOR independent companion of MTOR, complex 2 | MskImpact FoundationOneGenes |
| MDC1 | mediator of DNA-damage checkpoint 1 | MskImpact |
| CRKL | v-crk sarcoma virus CT10 oncogene homolog (avian)-like | MskImpact FoundationOneGenes |
| HIST1H2BB | histone cluster 1, H2bb | MskImpact |
| CBFB | core-binding factor, beta subunit | MskImpact FoundationOneGenes |
| PHOX2B | paired-like homeobox 2b | MskImpact |
| FLT1 | fms-related tyrosine kinase 1 (vascular endothelial growth factor/vasc | MskImpact FoundationOneGenes |
| CDK12 | cyclin-dependent kinase 12 | MskImpact FoundationOneGenes |
| BRAF | v-raf murine sarcoma viral oncogene homolog B1 | MskImpact CarisMolecularIntelligence FoundationOneGenes |
| RMI2 | RMI2, RecQ mediated genome instability 2, homolog (S. cerevisiae) | MskImpact |
| ATR | ataxia telangiectasia and Rad3 related | MskImpact FoundationOneGenes |
| PTCH1 | patched 1 | MskImpact FoundationOneGenes |
| JAK2 | Janus kinase 2 | MskImpact CarisMolecularIntelligence FoundationOneGenes |
| SMO | smoothened, frizzled family receptor | MskImpact CarisMolecularIntelligence FoundationOneGenes |
| PRDM1 | PR domain containing 1, with ZNF domain | MskImpact FoundationOneGenes |
| CD276 | CD276 molecule | MskImpact |
| TGFBR1 | transforming growth factor, beta receptor 1 | MskImpact |
| BARD1 | BRCA1 associated RING domain 1 | MskImpact FoundationOneGenes |
| DNMT3B | DNA (cytosine-5-)-methyltransferase 3 beta | MskImpact |
| BTK | Bruton agammaglobulinemia tyrosine kinase | MskImpact FoundationOneGenes |
| NTRK1 | neurotrophic tyrosine kinase, receptor, type 1 | MskImpact FoundationOneGenes |
| KEAP1 | kelch-like ECH-associated protein 1 | MskImpact FoundationOneGenes |
| CASP8 | caspase 8, apoptosis-related cysteine peptidase | MskImpact |
| STAG2 | stromal antigen 2 | MskImpact FoundationOneGenes |
| WT1 | Wilms tumor 1 | MskImpact FoundationOneGenes |
| CTCF | CCCTC-binding factor (zinc finger protein) | MskImpact FoundationOneGenes |
| EIF1AX | eukaryotic translation initiation factor 1A, X-linked | MskImpact |
| PIK3R2 | phosphoinositide-3-kinase, regulatory subunit 2 (beta) | MskImpact FoundationOneGenes |
| AURKB | aurora kinase B | MskImpact FoundationOneGenes |
| AMER1 | /genes/AMER1 | MskImpact |
| FGF3 | fibroblast growth factor 3 | MskImpact FoundationOneGenes |
| KDM5C | lysine (K)-specific demethylase 5C | MskImpact FoundationOneGenes |
| PTPRT | protein tyrosine phosphatase, receptor type, T | MskImpact |
| NKX2-1 | NK2 homeobox 1 | MskImpact FoundationOneGenes |
| PTPRD | protein tyrosine phosphatase, receptor type, D | MskImpact |
| CCND2 | cyclin D2 | MskImpact FoundationOneGenes |
| ERCC5 | excision repair cross-complementing rodent repair deficiency, comple | MskImpact |
| SOX9 | SRY (sex determining region Y)-box 9 | MskImpact |
| PTPN11 | protein tyrosine phosphatase, non-receptor type 11 | MskImpact CarisMolecularIntelligence FoundationOneGenes |
| NTRK3 | neurotrophic tyrosine kinase, receptor, type 3 | MskImpact FoundationOneGenes |
| BRCA1 | breast cancer 1, early onset | MskImpact CarisMolecularIntelligence FoundationOneGenes |
| RPS6KA4 | ribosomal protein S6 kinase, 90kDa, polypeptide 4 | MskImpact |
| PPP2R1A | protein phosphatase 2, regulatory subunit A, alpha | MskImpact FoundationOneGenes |
| CDK4 | cyclin-dependent kinase 4 | MskImpact FoundationOneGenes |
| RAF1 | v-raf-1 murine leukemia viral oncogene homolog 1 | MskImpact FoundationOneGenes |
| NCOR1 | nuclear receptor corepressor 1 | MskImpact |
| MCL1 | myeloid cell leukemia sequence 1 (BCL2-related) | MskImpact FoundationOneGenes |
| DNMT1 | DNA (cytosine-5-)-methyltransferase 1 | MskImpact |
| RBM10 | RNA binding motif protein 10 | MskImpact |
| MEF2B | myocyte enhancer factor 2B | MskImpact FoundationOneGenes |
| FOXP1 | forkhead box P1 | MskImpact |
| INPP4B | inositol polyphosphate-4-phosphatase, type II, 105kDa | MskImpact |
| RHOA | ras homolog family member A | MskImpact |
| IDH2 | isocitrate dehydrogenase 2 (NADP ), mitochondrial | MskImpact CarisMolecularIntelligence FoundationOneGenes |
| FLT4 | fms-related tyrosine kinase 4 | MskImpact FoundationOneGenes |
| IL7R | interleukin 7 receptor | MskImpact FoundationOneGenes |
| PAK1 | p21 protein (Cdc42/Rac)-activated kinase 1 | MskImpact |
| JAK3 | Janus kinase 3 | MskImpact CarisMolecularIntelligence FoundationOneGenes |
| SMAD3 | SMAD family member 3 | MskImpact |
| ARID1A | AT rich interactive domain 1A (SWI-like) | MskImpact FoundationOneGenes |
| HIST1H3A | histone cluster 1, H3a | MskImpact |
| TET2 | tet methylcytosine dioxygenase 2 | MskImpact FoundationOneGenes |
| FAT1 | FAT tumor suppressor homolog 1 (Drosophila) | MskImpact |
| XIAP | X-linked inhibitor of apoptosis | MskImpact |
| PIK3C2G | phosphatidylinositol-4-phosphate 3-kinase, catalytic subunit type 2 ga | MskImpact |
| CHEK1 | checkpoint kinase 1 | MskImpact FoundationOneGenes |
| ETV6 | ets variant 6 | MskImpact FoundationOneGenes |
| DAXX | death-domain associated protein | MskImpact FoundationOneGenes |
| ALOX12B | arachidonate 12-lipoxygenase, 12R type | MskImpact |
| KDM5A | lysine (K)-specific demethylase 5A | MskImpact FoundationOneGenes |
| CDK8 | cyclin-dependent kinase 8 | MskImpact FoundationOneGenes |
| CUL3 | cullin 3 | MskImpact |
| FGFR1 | fibroblast growth factor receptor 1 | MskImpact CarisMolecularIntelligence FoundationOneGenes |
| PAX5 | paired box 5 | MskImpact FoundationOneGenes |
| CDKN2B | cyclin-dependent kinase inhibitor 2B (p15, inhibits CDK4) | MskImpact FoundationOneGenes |
| PIM1 | pim-1 oncogene | MskImpact |
| IKZF1 | IKAROS family zinc finger 1 (Ikaros) | MskImpact FoundationOneGenes |
| NTRK2 | neurotrophic tyrosine kinase, receptor, type 2 | MskImpact FoundationOneGenes |
| ETV1 | ets variant 1 | MskImpact FoundationOneGenes |
| U2AF1 | U2 small nuclear RNA auxiliary factor 1 | MskImpact |
| KLF4 | Kruppel-like factor 4 (gut) | MskImpact |
| TRAF7 | TNF receptor-associated factor 7, E3 ubiquitin protein ligase | MskImpact |

| FUBP1 | far upstream element (FUSE) binding protein 1 | MskImpact |
| --- | --- | --- |
| MTOR | mechanistic target of rapamycin (serine/threonine kinase) | MskImpact FoundationOneGenes |
| NOTCH3 | notch 3 | MskImpact |
| FOXL2 | forkhead box L2 | MskImpact FoundationOneGenes |
| HIST1H2AC | histone cluster 1, H2ac | MskImpact |
| PLK2 | polo-like kinase 2 | MskImpact |
| XPO1 | exportin 1 (CRM1 homolog, yeast) | MskImpact FoundationOneGenes |
| GREM1 | gremlin 1 | MskImpact |
| SPOP | speckle-type POZ protein | MskImpact FoundationOneGenes |
| SETD2 | SET domain containing 2 | MskImpact FoundationOneGenes |
| ERCC2 | excision repair cross-complementing rodent repair deficiency, comple | MskImpact |
| ERCC4 | excision repair cross-complementing rodent repair deficiency, comple | MskImpact |
| FAM175A | family with sequence similarity 175, member A | MskImpact |
| REL | v-rel reticuloendotheliosis viral oncogene homolog (avian) | MskImpact |
| IL10 | interleukin 10 | MskImpact |
| BCL2L14 | BCL2-like 14 (apoptosis facilitator) | MskImpact |
| MAPK1 | mitogen-activated protein kinase 1 | MskImpact |
| PARK2 | parkinson protein 2, E3 ubiquitin protein ligase (parkin) | MskImpact |
| PMS2 | PMS2 postmeiotic segregation increased 2 (S. cerevisiae) | MskImpact |
| CDC73 | cell division cycle 73, Paf1/RNA polymerase II complex component, h | MskImpact FoundationOneGenes |
| PDCD1 | programmed cell death 1 | MskImpact |
| MDM4 | Mdm4 p53 binding protein homolog (mouse) | MskImpact FoundationOneGenes |
| SDHC | succinate dehydrogenase complex, subunit C, integral membrane pro | MskImpact |
| NBN | nibrin | MskImpact |
| B2M | beta-2-microglobulin | MskImpact |
| FLCN | folliculin | MskImpact |
| FANCC | Fanconi anemia, complementation group C | MskImpact FoundationOneGenes |
| IRS2 | insulin receptor substrate 2 | MskImpact FoundationOneGenes |
| TMEM127 | transmembrane protein 127 | MskImpact |
| ATRX | alpha thalassemia/mental retardation syndrome X-linked | MskImpact FoundationOneGenes |
| RAD54L | RAD54-like (S. cerevisiae) | MskImpact |
| PIK3C3 | phosphatidylinositol 3-kinase, catalytic subunit type 3 | MskImpact |
| RAD52 | RAD52 homolog (S. cerevisiae) | MskImpact |
| SDHD | succinate dehydrogenase complex, subunit D, integral membrane pro | MskImpact |
| RECQL4 | RecQ protein-like 4 | MskImpact |
| KMT2A | /genes/KMT2A | MskImpact |
| ARID5B | AT rich interactive domain 5B (MRF1-like) | MskImpact |
| SPEN | spen homolog, transcriptional regulator (Drosophila) | MskImpact FoundationOneGenes |
| DICER1 | dicer 1, ribonuclease type III | MskImpact |
| PNRC1 | proline-rich nuclear receptor coactivator 1 | MskImpact |
| PDGFRA | platelet-derived growth factor receptor, alpha polypeptide | CarisMolecularIntelligence FoundationOneGenes |
| ERCC1 | excision repair cross-complementing rodent repair deficiency, comple | CarisMolecularIntelligence |
| AREG | amphiregulin | CarisMolecularIntelligence |
| EREG | epiregulin | CarisMolecularIntelligence |
| RRM1 | ribonucleotide reductase M1 | CarisMolecularIntelligence |
| SPARC | secreted protein, acidic, cysteine-rich (osteonectin) | CarisMolecularIntelligence |
| MGMT | O-6-methylguanine-DNA methyltransferase | CarisMolecularIntelligence |
| TUBB3 | tubulin, beta 3 class III | CarisMolecularIntelligence |
| TLE3 | transducin-like enhancer of split 3 (E(sp1) homolog, Drosophila) | CarisMolecularIntelligence |
| PRKDC | protein kinase, DNA-activated, catalytic polypeptide | FoundationOneGenes |
| FGF14 | fibroblast growth factor 14 | FoundationOneGenes |
| NUP93 | nucleoporin 93kDa | FoundationOneGenes |
| FGF10 | fibroblast growth factor 10 | FoundationOneGenes |
| INHBA | inhibin, beta A | FoundationOneGenes |
| GNA13 | guanine nucleotide binding protein (G protein), alpha 13 | FoundationOneGenes |
| FAM123B | family with sequence similarity 123B | FoundationOneGenes |
| FANCL | Fanconi anemia, complementation group L | FoundationOneGenes |
| PAK3 | p21 protein (Cdc42/Rac)-activated kinase 3 | FoundationOneGenes |
| GID4 | GID complex subunit 4, VID24 homolog (S. cerevisiae) | FoundationOneGenes |
| FGF23 | fibroblast growth factor 23 | FoundationOneGenes |
| LRP1B | low density lipoprotein receptor-related protein 1B | FoundationOneGenes |
| FANCF | Fanconi anemia, complementation group F | FoundationOneGenes |
| SOX2 | SRY (sex determining region Y)-box 2 | FoundationOneGenes |
| FANCG | Fanconi anemia, complementation group G | FoundationOneGenes |
| FANCD2 | Fanconi anemia, complementation group D2 | FoundationOneGenes |
| SOCS1 | suppressor of cytokine signaling 1 | FoundationOneGenes |
| SOX10 | SRY (sex determining region Y)-box 10 | FoundationOneGenes |
| ETV4 | ets variant 4 | FoundationOneGenes |
| FANCE | Fanconi anemia, complementation group E | FoundationOneGenes |
| STAT4 | signal transducer and activator of transcription 4 | FoundationOneGenes |
| CEBPA | CCAAT/enhancer binding protein (C/EBP), alpha | FoundationOneGenes |
| ZNF217 | zinc finger protein 217 | FoundationOneGenes |
| ETV5 | ets variant 5 | FoundationOneGenes |
| MYCL1 | v-myc myelocytomatosis viral oncogene homolog 1, lung carcinoma d | FoundationOneGenes |
| BCL2L2 | BCL2-like 2 | FoundationOneGenes |
| MLL | myeloid/lymphoid or mixed-lineage leukemia (trithorax homolog, Dros | FoundationOneGenes |
| BCR | breakpoint cluster region | FoundationOneGenes |
| KAT6A | K(lysine) acetyltransferase 6A | FoundationOneGenes |
| GPR124 | G protein-coupled receptor 124 | FoundationOneGenes |
| C11ORF30 | chromosome 11 open reading frame 30 | FoundationOneGenes |
| FGF6 | fibroblast growth factor 6 | FoundationOneGenes |
| CTNNA1 | catenin (cadherin-associated protein), alpha 1, 102kDa | FoundationOneGenes |
| PDK1 | pyruvate dehydrogenase kinase, isozyme 1 | FoundationOneGenes |
| ARFRP1 | ADP-ribosylation factor related protein 1 | FoundationOneGenes |
| WISP3 | WNT1 inducible signaling pathway protein 3 | FoundationOneGenes |
| KLHL6 | kelch-like 6 (Drosophila) | FoundationOneGenes |
| CD79A | CD79a molecule, immunoglobulin-associated alpha | FoundationOneGenes |
| NFKBIA | nuclear factor of kappa light polypeptide gene enhancer in B-cells inh | FoundationOneGenes |
| BCORL1 | BCL6 corepressor-like 1 | FoundationOneGenes |
| EWSR1 | Ewing sarcoma breakpoint region 1 | FoundationOneGenes |
| ZNF703 | zinc finger protein 703 | FoundationOneGenes |
| MLL2 | myeloid/lymphoid or mixed-lineage leukemia 2 | FoundationOneGenes |
