# Supplementary material for: Genetic heterogeneity and actionable mutations in HER2-positive primary breast cancers and their brain metastases
Source: Oncotarget. 2018 Apr 17;9(29):20617–30. doi: 10.18632/oncotarget.25041 (PMC5945519; doi:10.18632/oncotarget.25041)
Supplement: Supplementary file 5 [file oncotarget-09-20617-s005.doc]

**Supplementary Table 6: List of potential clinical actionable genes according to the http://oncokb.org/api/v1/utils/allActionableVariants.txt**

| **Gene** | **Alteration** | **Cancer Type** | **Level** | **Drugs(s)** | **PMIDs for drug** | **Abstracts for drug** |
| --- | --- | --- | --- | --- | --- | --- |
| EGFR | L833V | Non-Small Cell Lung Cancer | 1 | Afatinib, Erlotinib, Gefitinib | 21949883, 23912954, 25130612, 20808254, 21422421 |  |
|  |  |  |  |  |  |  |
|  |  |  |  |  |  |  |
| EGFR | E709K | Non-Small Cell Lung Cancer | 1 | Afatinib, Erlotinib, Gefitinib | 23566546, 18000506, 25179728, 25130612, 23749122, 15710947 |  |
|  |  |  |  |  |  |  |
|  |  |  |  |  |  |  |
|  |  |  |  |  | 21531810, 15638953, 14570950, 15118073,  26051236, 21670455, 18408761, 16011858,  16187797, 24285021, 20573926 |  |
| EGFR | G719A | Non-Small Cell Lung Cancer | 1 | Afatinib, Erlotinib, Gefitinib |  |  |
|  |  |  |  |  |  |  |
|  |  |  |  |  | 15638953, 22452895, 25589191, 23816960,  14570950, 22285168, 20022809, 19692680,  21670455, 18408761, 22370314, 20573926 |  |
| EGFR | L858R | Non-Small Cell Lung Cancer | 1 | Erlotinib, Afatinib, Gefitinib |  |  |
|  |  |  |  |  |  |  |
| EGFR | L858R | Non-Small Cell Lung Cancer | 4 | Osimertinib | 27198352 |  |
|  |  |  |  |  | 15638953, 22452895, 25589191, 23816960,  14570950, 22285168, 20022809, 19692680,  21670455, 18408761, 22370314, 20573926 |  |
| EGFR | Exon 19 deletion/insertion | Non-Small Cell Lung Cancer | 1 | Erlotinib, Afatinib, Gefitinib |  |  |
|  |  |  |  |  |  |  |
| EGFR | L861Q | Non-Small Cell Lung Cancer | 1 | Afatinib, Erlotinib, Gefitinib | 21531810, 15638953, 14570950, 18408761,  21252719 |  |
|  |  |  |  |  |  |  |
|  |  |  |  |  |  |  |
| EGFR | EGFR-KDD | Non-Small Cell Lung Cancer | 1 | Afatinib, Erlotinib, Gefitinib | 15638953, 14570950, 26286086, 18408761 |  |
|  |  |  |  |  | 21531810, 15638953, 14570950, 15118073,  26051236, 21670455, 18408761, 16011858,  16187797, 24285021, 20573926 |  |
| EGFR | G719D | Non-Small Cell Lung Cancer | 1 | Afatinib, Erlotinib, Gefitinib |  |  |
|  |  |  |  |  |  |  |
| EGFR | L747P | Non-Small Cell Lung Cancer | 1 | Afatinib, Erlotinib, Gefitinib | 22190593 |  |
| EGFR | E709_T710delinsD | Non-Small Cell Lung Cancer | 1 | Afatinib, Erlotinib, Gefitinib | 21531810, 15638953, 14570950, 18408761,  26206867 |  |
|  |  |  |  |  |  |  |
|  |  |  |  |  |  |  |
| EGFR | A763_Y764insFQEA | Non-Small Cell Lung Cancer | 1 | Afatinib, Erlotinib, Gefitinib | 15638953, 24353160, 14570950, 23969006,  18408761 |  |
|  |  |  |  |  |  |  |
|  |  |  |  |  |  |  |
| EGFR | T790M | Non-Small Cell Lung Cancer | R1 | Afatinib, Erlotinib, Gefitinib | 24478319, 26051236, 23816963 | Yang et al. Abstract# O03.05, IASLC 2013 http://library.iaslc.org/search- |
|  |  |  |  |  |  | speaker?search_speaker=17991 |
|  |  |  |  |  |  |  |
|  |  |  |  |  | 21531810, 15638953, 14570950, 15118073,  26051236, 21670455, 18408761, 16011858,  16187797, 24285021, 20573926 |  |
| EGFR | G719S | Non-Small Cell Lung Cancer | 1 | Afatinib, Erlotinib, Gefitinib |  |  |
|  |  |  |  |  |  |  |
| EGFR | Exon 20 insertion | Non-Small Cell Lung Cancer | 4 | AP32788, EGF816 | 26825170 | Gonzalvez, F. et al. Abstract# 2644, AACR Annual Meeting 2016 |
|  |  |  |  |  |  | http://cancerres.aacrjournals.org/content/76/14_Supplement/2644 |
|  |  |  |  |  |  |  |
| EGFR | S768I | Non-Small Cell Lung Cancer | 4 | AP32788 |  | Gonzalvez, F. et al. Abstract# 2644, AACR Annual Meeting 2016 |
|  |  |  |  |  |  | http://cancerres.aacrjournals.org/content/76/14_Supplement/2644 |
|  |  |  |  |  |  |  |
| EGFR | A750P | Non-Small Cell Lung Cancer | 1 | Afatinib, Erlotinib, Gefitinib | 19625781, 25179728, 15737014 |  |
|  |  |  |  |  | 15638953, 22452895, 25589191, 23816960,  14570950, 22285168, 20022809, 19692680,  21670455, 18408761, 22370314, 20573926 |  |
| EGFR | Exon 19 deletion | Non-Small Cell Lung Cancer | 1 | Erlotinib, Afatinib, Gefitinib |  |  |
|  |  |  |  |  |  |  |
| EGFR | L861R | Non-Small Cell Lung Cancer | 4 | AP32788 |  | Gonzalvez, F. et al. Abstract# 2644, AACR Annual Meeting 2016 |
|  |  |  |  |  |  | http://cancerres.aacrjournals.org/content/76/14_Supplement/2644 |
|  |  |  |  |  |  |  |
| EGFR | L861R | Non-Small Cell Lung Cancer | 1 | Afatinib, Erlotinib, Gefitinib | 21531810, 15638953, 14570950, 18408761,  21252719 |  |
|  |  |  |  |  |  |  |
|  |  |  |  |  |  |  |
|  |  |  |  |  | 23371856, 24065731, 19536777, 24353160,  21764376, 18676761, 24893891, 23328547,  1867676, 15897572, 17686547 |  |
| EGFR | Exon 20 insertion | Non-Small Cell Lung Cancer | R1 | Afatinib, Erlotinib, Gefitinib |  |  |
|  |  |  |  |  |  |  |
|  |  |  |  |  | 21531810, 15638953, 14570950, 15118073,  26051236, 21670455, 18408761, 16011858,  16187797, 24285021, 20573926 |  |
| EGFR | G719C | Non-Small Cell Lung Cancer | 1 | Afatinib, Erlotinib, Gefitinib |  |  |
|  |  |  |  |  |  |  |
| EGFR | Exon 19 insertion | Non-Small Cell Lung Cancer | 4 | Osimertinib | 27198352 |  |
| EGFR | G719A | Non-Small Cell Lung Cancer | 4 | AP32788 |  | Gonzalvez, F. et al. Abstract# 2644, AACR Annual Meeting 2016 |
|  |  |  |  |  |  | http://cancerres.aacrjournals.org/content/76/14_Supplement/2644 |
|  |  |  |  |  |  |  |
| EGFR | Exon 19 deletion | Non-Small Cell Lung Cancer | 4 | Osimertinib | 27198352 |  |
| EGFR | S768I | Non-Small Cell Lung Cancer | 1 | Gefitnib, Afatinib, Erlotinib | 17285735, 15638953, 25521405, 14570950,  26051236, 18408761 |  |
|  |  |  |  |  |  |  |
|  |  |  |  |  |  |  |
| EGFR | T790M | Non-Small Cell Lung Cancer | 1 | Osimertinib | 25923549 |  |
| EGFR | Exon 19 insertion | Non-Small Cell Lung Cancer | 1 | Afatinib, Erlotinib, Gefitinib | 22190593 |  |
| ROS1 | D2033N | Non-Small Cell Lung Cancer | 3A | Cabozantinib | 26673800 |  |
| ROS1 | Fusions | Non-Small Cell Lung Cancer | 1 | Crizotinib | 25264305 |  |
| CDK4 | Amplification | Well-Differentiated Liposarcoma | 2A | Abemaciclib, Palbociclib | 23569312, 20601955, 26324739, 21610706 |  |
| CDK4 | Amplification | Dedifferentiated Liposarcoma | 2A | Abemaciclib, Palbociclib | 23569312, 20601955, 26324739, 21610706 |  |
| CDKN2A | Oncogenic Mutations | Breast Cancer | 4 | Palbociclib + Letrozole | 26715889, 25524798 |  |
| CDKN2A | Oncogenic Mutations | Esophagogastric Cancer | 4 | Palbociclib | 26380006 |  |
| ERBB2 | Amplification | Breast Cancer | 3A | Neratinib | 20142587, 22967996, 24077916, 23632474,  25287822, 19318484 |  |
|  |  |  |  |  |  |  |
|  |  |  |  |  |  |  |
| ERBB2 | Amplification | Esophagogastric Cancer | 1 | Trastuzumab | 20728210 |  |
| ERBB2 | V659E | Non-Small Cell Lung Cancer | 3A | Lapatinib | 23950206, 19791830 |  |
| ERBB2 | Exon 20 insertions | Non-Small Cell Lung Cancer | 4 | AP32788 |  | Gonzalvez, F. et al. Abstract# 2644, AACR Annual Meeting 2016 |
|  |  |  |  |  |  | http://cancerres.aacrjournals.org/content/76/14_Supplement/2644 |
|  |  |  |  |  |  |  |
| ERBB2 | Oncogenic Mutations | Breast Cancer | 3A | Neratinib | 23220880 | Hyman et al. Abstract# PD5-05, AACR 2016 |
|  |  |  |  |  |  | http://cancerres.aacrjournals.org/content/76/4_Supplement/PD5-05 |
|  |  |  |  |  |  |  |
|  |  |  |  |  | 10561337, 21768458, 22149875, 16236738,  19060928, 19786658, 11248153, 17591827,  16452222, 16236737, 22153890, 22257673,  23020162, 16091755, 23602601, 20730488,  23871490, 17192538 |  |
|  |  |  |  |  |  |  |
| ERBB2 | Amplification | Breast Cancer | 1 | umab + Trastuzumab, Ado-trastuzumab em |  |  |
|  |  |  |  |  |  |  |
|  |  |  |  |  |  |  |
| ERBB2 | Exon 20 insertions/deletions | Non-Small Cell Lung Cancer | 4 | AP32788 |  | Gonzalvez, F. et al. Abstract# 2644, AACR Annual Meeting 2016 |
|  |  |  |  |  |  | http://cancerres.aacrjournals.org/content/76/14_Supplement/2644 |
|  |  |  |  |  |  |  |
| ERCC2 | Oncogenic Mutations | Bladder Cancer | 3A | Cisplatin | 12208738, 25096233, 27310333 |  |
| ABL1 | BCR-ABL1 Fusion | Chronic Myelogenous Leukemia | 1 | Nilotinib, Imatinib, Dasatnib | 11287973, 12637609, 20525993, 20525995,  22160483, 11287972, 23502220 |  |
|  |  |  |  |  |  |  |
|  |  |  |  |  |  |  |
| ABL1 | BCR-ABL1 Fusion | Acute Lymphoid Leukemia | 1 | Dasatinib, Imatinib | 17496201, 11287973, 20131302, 12200353 |  |
| PDGFRA | Q579R | Gastrointestinal Stromal Tumor | 2A | Imatinib | 24963404, 15928335 |  |
| PDGFRA | E311_K312del | Gastrointestinal Stromal Tumor | 2A | Imatinib | 24963404, 15928335 |  |
| PDGFRA | N659K | Gastrointestinal Stromal Tumor | 2A | Imatinib | 24963404, 15928335 |  |
| PDGFRA | Y555C | Gastrointestinal Stromal Tumor | 2A | Imatinib | 24963404, 15928335 |  |
| PDGFRA | N659R | Gastrointestinal Stromal Tumor | 2A | Imatinib | 24963404, 15928335 |  |
| PDGFRA | R748G | Gastrointestinal Stromal Tumor | 2A | Imatinib | 24963404, 15928335 |  |
| PDGFRA | N659S | Gastrointestinal Stromal Tumor | 2A | Imatinib | 24963404, 15928335 |  |
| PDGFRA | V561A | Gastrointestinal Stromal Tumor | 2A | Imatinib | 24963404, 15928335 |  |
| PDGFRA | H650Q | Gastrointestinal Stromal Tumor | 2A | Imatinib | 24963404, 15928335 |  |
| PDGFRA | 560_561insER | Gastrointestinal Stromal Tumor | 2A | Imatinib | 24963404, 15928335 |  |
| PDGFRA | D842V | Gastrointestinal Stromal Tumor | 2A | Dasatinib | 15928335, 18794084, 17419150 | Trent et al. Abstract# 10006, ASCO 2011 |
|  |  |  |  |  |  | http://meetinglibrary.asco.org/content/79120-102 |
|  |  |  |  |  |  |  |
| PDGFRA | 544_L545insAVLVLLVIVIISL | Gastrointestinal Stromal Tumor | 2A | Imatinib | 24963404, 15928335 |  |
| PDGFRA | H845Y | Gastrointestinal Stromal Tumor | 2A | Imatinib | 24963404, 15928335 |  |
| PDGFRA | Fusions | Myelodysplasia | 1 | Imatinib | 12944919, 17555450, 12660384, 16498388,  14504092, 16845659, 15034867 |  |
|  |  |  |  |  |  |  |
|  |  |  |  |  |  |  |
| PDGFRA | A633T | Gastrointestinal Stromal Tumor | 2A | Imatinib | 24963404, 15928335 |  |
|  |  |  |  |  | 25905001, 17087936, 12949711, 24963404,  22718859, 15928335, 18955458, 23752188,  22745105, 15685537, 18794084 |  |
| PDGFRA | D842V | Gastrointestinal Stromal Tumor | R1 | Imatinib |  |  |
|  |  |  |  |  |  |  |
| PDGFRA | Y375_K455del | Gastrointestinal Stromal Tumor | 2A | Imatinib | 24963404, 15928335 |  |
| PDGFRA | Fusions | Myeloproliferative Neoplasm | 1 | Imatinib | 12944919, 17555450, 12660384, 16498388,  14504092, 16845659, 15034867 |  |
|  |  |  |  |  |  |  |
|  |  |  |  |  |  |  |
|  |  |  |  |  | 16754777, 12781364, 19212337, 12660384, |  |
| PDGFRA | FIP1L1-PDGFRA Fusion | Leukemia | 1 | Imatinib | 24963404, 14504092, 24407160, 27021554, |  |
|  |  |  |  |  | 22745105, 21224473 |  |
| PDGFRA | V658A | Gastrointestinal Stromal Tumor | 2A | Imatinib | 24963404, 15928335 |  |
| PDGFRA | V536E | Gastrointestinal Stromal Tumor | 2A | Imatinib | 24963404, 15928335 |  |
| PDGFRA | V561D | Gastrointestinal Stromal Tumor | 2A | Imatinib | 24963404, 15928335 |  |
| PDGFRA | D568N | Gastrointestinal Stromal Tumor | 2A | Imatinib | 24963404, 15928335 |  |
| PDGFRA | D842_H845del | Gastrointestinal Stromal Tumor | 2A | Imatinib | 24963404, 15928335 |  |
| PDGFRA | Y849C | Gastrointestinal Stromal Tumor | 2A | Imatinib | 24963404, 15928335 |  |
| PDGFRA | S584L | Gastrointestinal Stromal Tumor | 2A | Imatinib | 24963404, 15928335 |  |
| PDGFRA | W559_R560del | Gastrointestinal Stromal Tumor | 2A | Imatinib | 24963404, 15928335 |  |
| PDGFRA | V561_I562insER | Gastrointestinal Stromal Tumor | 2A | Imatinib | 24963404, 15928335 |  |
| PDGFRA | V469A | Gastrointestinal Stromal Tumor | 2A | Imatinib | 24963404, 15928335 |  |
| PDGFRA | Y849S | Gastrointestinal Stromal Tumor | 2A | Imatinib | 24963404, 15928335 |  |
| PDGFRA | G853D | Gastrointestinal Stromal Tumor | 2A | Imatinib | 24963404, 15928335 |  |
| PDGFRA | C456_R481del | Gastrointestinal Stromal Tumor | 2A | Imatinib | 24963404, 15928335 |  |
| PDGFRA | C456_N468del | Gastrointestinal Stromal Tumor | 2A | Imatinib | 24963404, 15928335 |  |
| PDGFRA | P577S | Gastrointestinal Stromal Tumor | 2A | Imatinib | 24963404, 15928335 |  |
| PDGFRA | H845_N848delinsP | Gastrointestinal Stromal Tumor | 2A | Imatinib | 24963404, 15928335 |  |
| PDGFRA | D842I | Gastrointestinal Stromal Tumor | 2A | Imatinib | 24963404, 15928335 |  |
| PDGFRA | D846Y | Gastrointestinal Stromal Tumor | 2A | Imatinib | 24963404, 15928335 |  |
| PDGFRA | D842_M844del | Gastrointestinal Stromal Tumor | 2A | Imatinib | 24963404, 15928335 |  |
| PDGFRA | C450_K451insMIEWMI | Gastrointestinal Stromal Tumor | 2A | Imatinib | 24963404, 15928335 |  |
| PDGFRA | R841K | Gastrointestinal Stromal Tumor | 2A | Imatinib | 24963404, 15928335 |  |
| PDGFRA | I843del | Gastrointestinal Stromal Tumor | 2A | Imatinib | 24963404, 15928335 |  |
| PDGFRA | N848K | Gastrointestinal Stromal Tumor | 2A | Imatinib | 24963404, 15928335 |  |
| PDGFRA | S566_E571delinsR | Gastrointestinal Stromal Tumor | 2A | Imatinib | 24963404, 15928335 |  |
|  |  |  |  |  | 16960151, 12676775, 12181402, 24963404,  14504092, 18950453, 24687085, 22897847,  1516603 |  |
| PDGFRB | Fusions | Myelodysplasia | 1 | Imatinib |  |  |
|  |  |  |  |  |  |  |
| PDGFRB | Fusions | Dermatofibrosarcoma Protuberans | 1 | Imatinib | 20439456, 21128251, 24963404, 15503291,  15746584, 15681532, 19620561  16960151, 12676775, 12181402, 24963404,  14504092, 18950453, 24687085, 22897847,  1516603 |  |
|  |  |  |  |  |  |  |
|  |  |  |  |  |  |  |
|  |  |  |  |  |  |  |
| PDGFRB | Fusions | Myeloproliferative Neoplasm | 1 | Imatinib |  |  |
|  |  |  |  |  |  |  |
| ESR1 | D538G | Breast Cancer | 4 | GDC-0810 | 24185512, 27410477 |  |
| ESR1 | Oncogenic Mutations | Breast Cancer | 3A | AZD9496, Fulvestrant | 27986707, 27269946 |  |
| ESR1 | Y537S | Breast Cancer | 4 | GDC-0810 | 24185512, 27410477 |  |
|  |  |  |  |  | 16981987, 15595939, 24729041, 23312829,  10533067, 26540169, 23158522, 21047224,  17304050, 24143074 |  |
| TSC1 | Oncogenic Mutations | CNS Cancer | 2A | Everolimus |  |  |
|  |  |  |  |  |  |  |
| TSC1 | Oncogenic Mutations | Renal Cell Carcinoma | 2A | Everolimus | 26831717 |  |

|  |  |  |  |  | 16981987, 15595939, 24729041, 23312829, |  |
| --- | --- | --- | --- | --- | --- | --- |
| TSC2 | Oncogenic Mutations | CNS Cancer | 2A | Everolimus | 10533067, 26540169, 23158522, 21047224, |  |
|  |  |  |  |  | 17304050, 24143074 |  |
| MDM2 | Amplification | Liposarcoma | 4 | SAR405838 | 26475335 |  |
| MDM2 | Amplification | Liposarcoma | 3A | DS-3032b, RG7112 | 23084521 | Gounder et al. Abstract# 2581, ASCO 2016 |
|  |  |  |  |  |  | http://meetinglibrary.asco.org/content/166204-176 |
|  |  |  |  |  |  |  |
| EZH2 | Oncogenic Mutations | Diffuse Large B-Cell Lymphoma | 4 | Tazemetostat, GSK126 | 24563539, 23051747 | Ribrag et al. American Society of Hematology 2015 |
|  |  |  |  |  |  | http://www.bloodjournal.org/content/126/23/473?sso-checked=true |
|  |  |  |  |  |  |  |
| MET | 963_D1010splice | Non-Small Cell Lung Cancer | 2A | Crizotinib | 25971939, 26729443, 25971938, 15735036, |  |
|  |  |  |  |  | 26215952, 16397241, 19096300 |  |
|  |  |  |  |  |  |  |
| MET | X1007_splice | Non-Small Cell Lung Cancer | 2A | Crizotinib | 25971939, 26729443, 25971938, 15735036, |  |
|  |  |  |  |  | 26215952, 16397241, 19096300 |  |
|  |  |  |  |  |  |  |
| MET | X1008_splice | Non-Small Cell Lung Cancer | 3A | Cabozatinib, Capmatinib | 21918175, 25971939, 25971938 |  |
| MET | D1010Y | Non-Small Cell Lung Cancer | 3A | Cabozatinib, Capmatinib | 21918175, 25971939, 25971938 |  |
| MET | D1010N | Non-Small Cell Lung Cancer | 2A | Crizotinib | 25971939, 26729443, 25971938, 15735036, |  |
|  |  |  |  |  | 16397241, 19096300 |  |
|  |  |  |  |  |  |  |
| MET | Amplification | Renal Cell Carcinoma | 2A | Cabozantinib | 27279544, 27462141 |  |
| MET | D1010H | Non-Small Cell Lung Cancer | 2A | Crizotinib | 25971939, 26729443, 25971938, 15735036, |  |
|  |  |  |  |  | 16397241, 19096300 |  |
|  |  |  |  |  |  |  |
| MET | X1007_splice | Non-Small Cell Lung Cancer | 3A | Cabozatinib, Capmatinib | 21918175, 25971939, 25971938 |  |
| MET | X1008_splice | Non-Small Cell Lung Cancer | 2A | Crizotinib | 25971939, 26729443, 25971938, 15735036, |  |
|  |  |  |  |  | 26215952, 16397241, 19096300 |  |
|  |  |  |  |  |  |  |
| MET | 981_1028splice | Non-Small Cell Lung Cancer | 3A | Cabozatinib, Capmatinib | 21918175, 25971939, 25971938 |  |
| MET | X1009_splice | Non-Small Cell Lung Cancer | 2A | Crizotinib | 25971939, 26729443, 25971938, 15735036, |  |
|  |  |  |  |  | 26215952, 16397241, 19096300 |  |
|  |  |  |  |  |  |  |
| MET | D1010N | Non-Small Cell Lung Cancer | 3A | Cabozatinib, Capmatinib | 21918175, 25971939, 25971938 |  |
| MET | X1009_splice | Non-Small Cell Lung Cancer | 3A | Cabozatinib, Capmatinib | 21918175, 25971939, 25971938 |  |
| MET | X963_splice | Non-Small Cell Lung Cancer | 2A | Crizotinib | 25971939, 26729443, 25971938, 15735036, |  |
|  |  |  |  |  | 26215952, 16397241, 19096300 |  |
|  |  |  |  |  |  |  |
| MET | X1006_splice | Non-Small Cell Lung Cancer | 3A | Cabozatinib, Capmatinib | 21918175, 25971939, 25971938 |  |
| MET | Amplification | Non-Small Cell Lung Cancer | 2A | Crizotinib | 21623265 | Camidge et al. Abstract# 8001, ASCO 2014 |
|  |  |  |  |  |  | http://meetinglibrary.asco.org/content/132030-144 |
|  |  |  |  |  |  |  |
| MET | D1010Y | Non-Small Cell Lung Cancer | 2A | Crizotinib | 25971939, 26729443, 25971938, 15735036, |  |
|  |  |  |  |  | 16397241, 19096300 |  |
|  |  |  |  |  |  |  |
| MET | D1010H | Non-Small Cell Lung Cancer | 3A | Cabozatinib, Capmatinib | 21918175, 25971939, 25971938 |  |
| MET | 981_1028splice | Non-Small Cell Lung Cancer | 2A | Crizotinib | 25971939, 26729443, 25971938, 15735036, |  |
|  |  |  |  |  | 26215952, 16397241, 19096300 |  |
|  |  |  |  |  |  |  |
| MET | 963_D1010splice | Non-Small Cell Lung Cancer | 3A | Cabozatinib, Capmatinib | 21918175, 25971939, 25971938 |  |
| MET | X963_splice | Non-Small Cell Lung Cancer | 3A | Cabozatinib, Capmatinib | 21918175, 25971939, 25971938 |  |
| MET | X1006_splice | Non-Small Cell Lung Cancer | 2A | Crizotinib | 25971939, 26729443, 25971938, 15735036, |  |
|  |  |  |  |  | 26215952, 16397241, 19096300 |  |
|  |  |  |  |  |  |  |
|  |  |  |  |  |  | Di Leo et al. Abstract# S4-07, SABCS 2016 |
| PIK3CA | Oncogenic Mutations | Endometrial Cancer | 4 | lvestrant, Fulvestrant + Taselisib, Buparlisib | 25877889 | https://www.sabcs.org/Portals/SABCS2016/Documents/SABCS-2016- |
|  |  |  |  |  |  | Abstracts.pdf?v=1; Janku et al. Abstract# PD5-5, AACR 2014 |
|  |  |  |  |  |  |  |
|  |  |  |  |  |  | http://cancerres.aacrjournals.org/content/75/9_Supplement/PD5-5 |
|  |  |  |  |  |  | Di Leo et al. Abstract# S4-07, SABCS 2016 |
| PIK3CA | Oncogenic Mutations | Ovarian Cancer | 4 | lvestrant, Fulvestrant + Taselisib, Buparlisib | 25877889 | https://www.sabcs.org/Portals/SABCS2016/Documents/SABCS-2016- |
|  |  |  |  |  |  | Abstracts.pdf?v=1; Janku et al. Abstract# PD5-5, AACR 2014 |
|  |  |  |  |  |  |  |
|  |  |  |  |  |  | http://cancerres.aacrjournals.org/content/75/9_Supplement/PD5-5 |
|  |  |  |  |  |  | Dickler et al. Abstract# 520, ASCO 2016 |
|  |  |  |  |  |  | http://meetinglibrary.asco.org/content/165518-176; Janku et al. |
|  |  |  |  |  | 21362200, 23903756, 24310736, 23662903, | Abstract# PD5-5, AACR 2014 |
|  |  |  |  |  |  | http://cancerres.aacrjournals.org/content/75/9_Supplement/PD5-5; Di |
|  |  |  |  |  | 25172762, 24076665, 22340590, 22049316, |  |
|  |  |  |  |  |  | Leo et al. Abstract# S4-07, SABCS 2016 |
|  |  |  |  |  | 24561032, 22355357, 20881279, 21169255, |  |
|  |  |  |  |  |  | https://www.sabcs.org/Portals/SABCS2016/Documents/SABCS-2016- |
| PIK3CA | Oncogenic Mutations | Breast Cancer | 3A | Copanlisib, Fulvestrant + Taselisib, GDC-00 | 24244612, 23850807, 24900266, 23721513, |  |
|  |  |  |  |  |  | Abstracts.pdf?v=1; Staben et al. Abstract# DDT02-01, AACR 2017 |
|  |  |  |  |  | 23136191, 23258246, 25877889, 24608574, |  |
|  |  |  |  |  |  | http://www.abstractsonline.com/pp8/#!/4292/presentation/11034; Di |
|  |  |  |  |  | 22065080, 22188813, 27672108, 23726034, |  |
|  |  |  |  |  |  | Leo et al. Abstract# S4-07, SABCS 2016 |
|  |  |  |  |  | 22653967 |  |
|  |  |  |  |  |  | https://www.sabcs.org/Portals/SABCS2016/Documents/SABCS-2016- |
|  |  |  |  |  |  |  |
|  |  |  |  |  |  | Abstracts.pdf?v=1; Juric D et al. Abstract# 2501, ASCO 2015 |
|  |  |  |  |  |  | http://meetinglibrary.asco.org/content/147739-156 |
|  |  |  |  |  |  | Michalarea et al. Abstract# CT323, AACR 2015 |
|  |  |  |  |  |  | http://www.abstractsonline.com/Plan/ViewAbstract.aspx?sKey=d60a28 |
| PIK3CA | Oncogenic Mutations | All Tumors | 4 | ARQ 751, GDC-0077, AZD5363 + Olaparib | 26469692 | c6-91a3-4a37-a419-d79f96801b30&cKey=edbea616-0ae5-4520-936b- |
|  |  |  |  |  |  | 897b8d25b2d6&mKey=%7b19573A54-AE8F-4E00-9C23- |
|  |  |  |  |  |  |  |
|  |  |  |  |  |  | BD6D62268424%7d; Staben et al. Abstract# DDT02-01, AACR 2017 |
|  |  |  |  |  |  | http://www.abstractsonline.com/pp8/#!/4292/presentation/11034 |
|  |  |  |  |  |  | Brake et al. Abstract# C176, AACR 2013 |
|  |  |  |  |  |  | http://mct.aacrjournals.org/content/12/11_Supplement/C176.short; |
|  |  |  |  |  | 27402769, 23844554, 23903756, 23085766, | Hudson et al. Abstract# 2665, AACR 2015 |
|  |  |  |  |  |  | http://www.abstractsonline.com/Plan/ViewAbstract.aspx?mID=3682&s |
|  |  |  |  |  | 24900266, 23275335, 27126994, 12177099, |  |
|  |  |  |  |  |  | Key=14323955-7e99-4856-aec7-b2f95e96b99b&cKey=7627fe75-d2b2- |
| PIK3CA | Oncogenic Mutations | Breast Cancer | 4 | + Fulvestrant, Alpelisib + Olaparib, AZD883 | 24774538, 25877889, 22188813, 22915752, |  |
|  |  |  |  |  |  | 4175-8cd6-98aaeb2524e2&mKey=19573a54-ae8f-4e00-9c23- |
|  |  |  |  |  | 22915751, 23394218, 24198241, 25425103, |  |
|  |  |  |  |  |  | bd6d62268424; Shah et al. Abstract# 79, ASCO 2015 |
|  |  |  |  |  | 23726034 |  |
|  |  |  |  |  |  | http://meetinglibrary.asco.org/content/145702-156; Juric et al. |
|  |  |  |  |  |  |  |
|  |  |  |  |  |  | Abstract# P3-14-01, AACR 2016 |
|  |  |  |  |  |  | http://cancerres.aacrjournals.org/content/76/4_Supplement/P3-14-01 |
| AKT1 | E17K | Ovarian Cancer | 3A | AZD5363 | 23394218, 26351323 | Hyman et al. Abstract# B109, AACR-NCI-EORTC 2015 |
|  |  |  |  |  |  | http://mct.aacrjournals.org/content/14/12_Supplement_2/B109 |
|  |  |  |  |  |  |  |
| AKT1 | E17K | Breast Cancer | 3A | AZD5363 | 22294718, 23394218, 26351323 | Hyman et al. Abstract# B109, AACR-NCI-EORTC 2015 |
|  |  |  |  |  |  | http://mct.aacrjournals.org/content/14/12_Supplement_2/B109 |
|  |  |  |  |  |  |  |
| AKT1 | E17K | All Tumors | 4 | ARQ 751 | 26469692 |  |
|  |  |  |  |  |  | Voss MH et al. Abstract# TPS2629, ASCO 2014 |
| FGFR1 | Amplification | Lung Squamous Cell Carcinoma | 3A | AZD4547, Debio1347 | 25465127, 23082000 | http://meetinglibrary.asco.org/content/127043-144; Paik PK et al. |
|  |  |  |  |  |  | Abstract# 8035, ASCO 2014 |
|  |  |  |  |  |  |  |
|  |  |  |  |  |  | http://meetinglibrary.asco.org/content/128825-144 |
| FGFR1 | BCR-FGFR1 Fusion | Leukemia | 4 | Ponatinib | 22781593, 26055304 |  |
| FGFR3 | K650T | Bladder Cancer | 3A | JNJ-42756493, Debio1347 | 26324363, 22837387 | Voss, MH et al. Abstract# TPS2629, ASCO 2014 |
|  |  |  |  |  |  | http://meetinglibrary.asco.org/content/127043-144 |
|  |  |  |  |  |  |  |
| FGFR3 | K650E | Breast Cancer | 4 | Debio1347 |  | Voss MH et al. Abstract# TPS2629, ASCO 2014 |
|  |  |  |  |  |  | http://meetinglibrary.asco.org/content/127043-144 |
|  |  |  |  |  |  |  |
| FGFR3 | S249C | Breast Cancer | 4 | Debio1347 |  | Voss MH et al. Abstract# TPS2629, ASCO 2014 |
|  |  |  |  |  |  | http://meetinglibrary.asco.org/content/127043-144 |
|  |  |  |  |  |  |  |
| FGFR3 | R248C | Breast Cancer | 4 | Debio1347 |  | Voss MH et al. Abstract# TPS2629, ASCO 2014 |
|  |  |  |  |  |  | http://meetinglibrary.asco.org/content/127043-144 |
|  |  |  |  |  |  |  |
| FGFR3 | K650N | Bladder Cancer | 3A | JNJ-42756493, Debio1347 | 26324363, 22837387 | Voss, MH et al. Abstract# TPS2629, ASCO 2014 |
|  |  |  |  |  |  | http://meetinglibrary.asco.org/content/127043-144 |
|  |  |  |  |  |  |  |
| FGFR3 | S371C | Bladder Cancer | 3A | JNJ-42756493, Debio1347 | 26324363, 22837387 | Voss, MH et al. Abstract# TPS2629, ASCO 2014 |
|  |  |  |  |  |  | http://meetinglibrary.asco.org/content/127043-144 |
|  |  |  |  |  |  |  |
| FGFR3 | K650R | Breast Cancer | 4 | Debio1347 |  | Voss MH et al. Abstract# TPS2629, ASCO 2014 |
|  |  |  |  |  |  | http://meetinglibrary.asco.org/content/127043-144 |
|  |  |  |  |  |  |  |
| FGFR3 | K650M | Breast Cancer | 4 | Debio1347 |  | Voss MH et al. Abstract# TPS2629, ASCO 2014 |
|  |  |  |  |  |  | http://meetinglibrary.asco.org/content/127043-144 |
|  |  |  |  |  |  |  |
| FGFR3 | S371C | Breast Cancer | 4 | Debio1347 |  | Voss MH et al. Abstract# TPS2629, ASCO 2014 |
|  |  |  |  |  |  | http://meetinglibrary.asco.org/content/127043-144 |
|  |  |  |  |  |  |  |
| FGFR3 | G370C | Bladder Cancer | 3A | JNJ-42756493, Debio1347 | 26324363, 22837387 | Voss, MH et al. Abstract# TPS2629, ASCO 2014 |
|  |  |  |  |  |  | http://meetinglibrary.asco.org/content/127043-144 |
|  |  |  |  |  |  |  |
| FGFR3 | K650N | Breast Cancer | 4 | Debio1347 |  | Voss MH et al. Abstract# TPS2629, ASCO 2014 |
|  |  |  |  |  |  | http://meetinglibrary.asco.org/content/127043-144 |
|  |  |  |  |  |  |  |
| FGFR3 | K650M | Bladder Cancer | 3A | JNJ-42756493, Debio1347 | 26324363, 22837387 | Voss, MH et al. Abstract# TPS2629, ASCO 2014 |
|  |  |  |  |  |  | http://meetinglibrary.asco.org/content/127043-144 |
|  |  |  |  |  |  |  |
| FGFR3 | S249C | Bladder Cancer | 3A | JNJ-42756493, Debio1347 | 26324363, 22837387 | Voss, MH et al. Abstract# TPS2629, ASCO 2014 |
|  |  |  |  |  |  | http://meetinglibrary.asco.org/content/127043-144 |
|  |  |  |  |  |  |  |
| FGFR3 | K650T | Breast Cancer | 4 | Debio1347 |  | Voss MH et al. Abstract# TPS2629, ASCO 2014 |
|  |  |  |  |  |  | http://meetinglibrary.asco.org/content/127043-144 |
|  |  |  |  |  |  |  |
| FGFR3 | K650R | Bladder Cancer | 3A | JNJ-42756493, Debio1347 | 26324363, 22837387 | Voss, MH et al. Abstract# TPS2629, ASCO 2014 |
|  |  |  |  |  |  | http://meetinglibrary.asco.org/content/127043-144 |
|  |  |  |  |  |  |  |
| FGFR3 | Y373C | Breast Cancer | 4 | Debio1347 |  | Voss MH et al. Abstract# TPS2629, ASCO 2014 |
|  |  |  |  |  |  | http://meetinglibrary.asco.org/content/127043-144 |
|  |  |  |  |  |  |  |
| FGFR3 | K650Q | Breast Cancer | 4 | Debio1347 |  | Voss MH et al. Abstract# TPS2629, ASCO 2014 |
|  |  |  |  |  |  | http://meetinglibrary.asco.org/content/127043-144 |
|  |  |  |  |  |  |  |
| FGFR3 | K650E | Bladder Cancer | 3A | JNJ-42756493, Debio1347 | 26324363, 22837387 | Voss, MH et al. Abstract# TPS2629, ASCO 2014 |
|  |  |  |  |  |  | http://meetinglibrary.asco.org/content/127043-144 |
|  |  |  |  |  |  |  |
| FGFR3 | K650Q | Bladder Cancer | 3A | JNJ-42756493, Debio1347 | 26324363, 22837387 | Voss, MH et al. Abstract# TPS2629, ASCO 2014 |
|  |  |  |  |  |  | http://meetinglibrary.asco.org/content/127043-144 |
|  |  |  |  |  |  |  |
| FGFR3 | Fusions | Bladder Cancer | 3A | JNJ-42756493, Debio1347 | 26324363, 22837387 | Voss, MH et al. Abstract# TPS2629, ASCO 2014 |
|  |  |  |  |  |  | http://meetinglibrary.asco.org/content/127043-144 |
|  |  |  |  |  |  |  |
| FGFR3 | G380R | Bladder Cancer | 3A | JNJ-42756493, Debio1347 | 26324363, 22837387 | Voss, MH et al. Abstract# TPS2629, ASCO 2014 |
|  |  |  |  |  |  | http://meetinglibrary.asco.org/content/127043-144 |
|  |  |  |  |  |  |  |
| FGFR3 | G370C | Breast Cancer | 4 | Debio1347 |  | Voss MH et al. Abstract# TPS2629, ASCO 2014 |
|  |  |  |  |  |  | http://meetinglibrary.asco.org/content/127043-144 |
|  |  |  |  |  |  |  |
| FGFR3 | Y373C | Bladder Cancer | 3A | JNJ-42756493, Debio1347 | 26324363, 22837387 | Voss, MH et al. Abstract# TPS2629, ASCO 2014 |
|  |  |  |  |  |  | http://meetinglibrary.asco.org/content/127043-144 |
|  |  |  |  |  |  |  |
| FGFR3 | R248C | Bladder Cancer | 3A | JNJ-42756493, Debio1347 | 26324363, 22837387 | Voss, MH et al. Abstract# TPS2629, ASCO 2014 |
|  |  |  |  |  |  | http://meetinglibrary.asco.org/content/127043-144 |
|  |  |  |  |  |  |  |
| FGFR3 | Fusions | Adrenocortical Carcinoma | 3A | JNJ-42756493, Debio1347 | 26324363, 22837387 | Voss, MH et al. Abstract# TPS2629, ASCO 2014 |
|  |  |  |  |  |  | http://meetinglibrary.asco.org/content/127043-144 |
|  |  |  |  |  |  |  |
| FGFR3 | Fusions | Glioma | 3A | JNJ-42756493, Debio1347 | 26324363, 22837387 | Voss, MH et al. Abstract# TPS2629, ASCO 2014 |
|  |  |  |  |  |  | http://meetinglibrary.asco.org/content/127043-144 |
|  |  |  |  |  |  |  |
| FGFR3 | G380R | Breast Cancer | 4 | Debio1347 |  | Voss MH et al. Abstract# TPS2629, ASCO 2014 |
|  |  |  |  |  |  | http://meetinglibrary.asco.org/content/127043-144 |
|  |  |  |  |  |  |  |
| FGFR2 | Fusions | Bladder Cancer | 3A | JNJ-42756493, Debio1347 | 26324363, 24122810 | Voss, MH et al. Abstract# TPS2629, ASCO 2014 |
|  |  |  |  |  |  | http://meetinglibrary.asco.org/content/127043-144 |
|  |  |  |  |  |  |  |
| FGFR2 | Fusions | Endometrial Cancer | 3A | JNJ-42756493, Debio1347 | 26324363, 24122810 | Voss, MH et al. Abstract# TPS2629, ASCO 2014 |
|  |  |  |  |  |  | http://meetinglibrary.asco.org/content/127043-144 |
|  |  |  |  |  |  |  |
|  |  |  |  |  |  | Voss, MH et al. Abstract# TPS2629, ASCO 2014 |
| FGFR2 | Fusions | Cholangiocarcinoma | 3A | BGJ398, Debio1347 | 27216979 | http://meetinglibrary.asco.org/content/127043-144; Javle et al. |
|  |  |  |  |  |  | Abstract# 335, ASCO 2016 |
|  |  |  |  |  |  |  |
|  |  |  |  |  |  | http://meetinglibrary.asco.org/content/159420-173 |
| FGFR2 | Fusions | Adrenocortical Carcinoma | 3A | JNJ-42756493, Debio1347 | 26324363, 24122810 | Voss, MH et al. Abstract# TPS2629, ASCO 2014 |
|  |  |  |  |  |  | http://meetinglibrary.asco.org/content/127043-144 |
|  |  |  |  |  |  |  |
| ALK | Fusions | Non-Small Cell Lung Cancer | 1 | Crizotinib, Alectinib, Ceritinib | 24670165, 26708155, 26598747, 26973324, |  |
|  |  |  |  |  | 23724913 |  |
|  |  |  |  |  |  |  |
| ALK | Oncogenic Mutations | Non-Small Cell Lung Cancer | 1 | Brigatinib | 25322323, 21502504, 27836716 | Kim et al. Abstract# 9007, ASCO 2016 |
|  |  |  |  |  |  | http://meetinglibrary.asco.org/content/165056-176 |
|  |  |  |  |  |  |  |
| ALK | Fusions | Soft Tissue Sarcoma | 2A | Crizotinib, Ceritinib | 24670165, 20979472, 23598171 |  |

| ALK | R1275Q | Embryonal Tumor | 4 | Crizotinib | 22072639, 23598171 |  |
| --- | --- | --- | --- | --- | --- | --- |
|  |  |  |  |  |  | Rossell et al. Abstract# 99O, ELCC 2015 |
|  |  |  |  |  |  | http://oncologypro.esmo.org/Meeting-Resources/ELCC-2015/Phase-1- |
| ALK | L1196M | Non-Small Cell Lung Cancer | 3A | Brigatinib | 25322323, 21502504 | 2-study-of-AP26113-in-patients-Pts-with-advanced-malignancies- |
|  |  |  |  |  |  | including-anaplastic-lymphoma-kinase-ALK-positive-non-small-cell-lung- |
|  |  |  |  |  |  |  |
|  |  |  |  |  |  | cancer-NSCLC-Analysis-of-safety-and-efficacy-at-selected-phase-2- |
|  |  |  |  |  |  | doses |
|  |  |  |  |  |  | Rossell et al. Abstract# 99O, ELCC 2015 |
|  |  |  |  |  |  | http://oncologypro.esmo.org/Meeting-Resources/ELCC-2015/Phase-1- |
| ALK | L1196Q | Non-Small Cell Lung Cancer | 3A | Brigatinib | 25322323, 21502504 | 2-study-of-AP26113-in-patients-Pts-with-advanced-malignancies- |
|  |  |  |  |  |  | including-anaplastic-lymphoma-kinase-ALK-positive-non-small-cell-lung- |
|  |  |  |  |  |  |  |
|  |  |  |  |  |  | cancer-NSCLC-Analysis-of-safety-and-efficacy-at-selected-phase-2- |
|  |  |  |  |  |  | doses |
| FLT3 | T3 internal tandem duplicatio | Acute Myeloid Leukemia | 3A | Sorafenib | 18230792 |  |
|  |  |  |  |  |  | Panknin et al. Abstract# 2645, AACR 2016 |
| IDH1 | R132C | All Tumors | 4 | BAY1436032, CB-839 | 28232670, 24523301 | http://cancerres.aacrjournals.org/content/76/14_Supplement/2645; |
|  |  |  |  |  |  | Matre et al. Abstract# 3763, ASH 2014 |
|  |  |  |  |  |  |  |
|  |  |  |  |  |  | http://www.bloodjournal.org/content/124/21/3763 |
| IDH1 | R132Q | Acute Myeloid Leukemia | 3A | AG-120 | 23393090, 23558169 | Hansen et al. Abstract# 3734, ASH 2014 |
|  |  |  |  |  |  | http://www.bloodjournal.org/content/124/21/3734?sso-checked=true |
|  |  |  |  |  |  |  |
| IDH1 | R132G | Acute Myeloid Leukemia | 3A | AG-120 | 23393090, 23558169 | Hansen et al. Abstract# 3734, ASH 2014 |
|  |  |  |  |  |  | http://www.bloodjournal.org/content/124/21/3734?sso-checked=true |
|  |  |  |  |  |  |  |
|  |  |  |  |  |  | Matre et al. Abstract# 3763, ASH 2014 |
| IDH1 | R132Q | All Tumors | 4 | BAY1436032, CB-839 | 28232670, 24523301 | http://www.bloodjournal.org/content/124/21/3763; Panknin et al. |
|  |  |  |  |  |  | Abstract# 2645, AACR 2016 |
|  |  |  |  |  |  |  |
|  |  |  |  |  |  | http://cancerres.aacrjournals.org/content/76/14_Supplement/2645 |
|  |  |  |  |  |  | Matre et al. Abstract# 3763, ASH 2014 |
| IDH1 | R132H | All Tumors | 4 | BAY1436032, CB-839 | 28232670, 24523301 | http://www.bloodjournal.org/content/124/21/3763; Panknin et al. |
|  |  |  |  |  |  | Abstract# 2645, AACR 2016 |
|  |  |  |  |  |  |  |
|  |  |  |  |  |  | http://cancerres.aacrjournals.org/content/76/14_Supplement/2645 |
|  |  |  |  |  |  | Panknin et al. Abstract# 2645, AACR 2016 |
| IDH1 | R132S | All Tumors | 4 | BAY1436032, CB-839 | 28232670, 24523301 | http://cancerres.aacrjournals.org/content/76/14_Supplement/2645; |
|  |  |  |  |  |  | Matre et al. Abstract# 3763, ASH 2014 |
|  |  |  |  |  |  |  |
|  |  |  |  |  |  | http://www.bloodjournal.org/content/124/21/3763 |
| IDH1 | R132H | Acute Myeloid Leukemia | 3A | AG-120 | 23393090, 23558169 | Hansen et al. Abstract# 3734, ASH 2014 |
|  |  |  |  |  |  | http://www.bloodjournal.org/content/124/21/3734?sso-checked=true |
|  |  |  |  |  |  |  |
| IDH1 | R132S | Acute Myeloid Leukemia | 3A | AG-120 | 23393090, 23558169 | Hansen et al. Abstract# 3734, ASH 2014 |
|  |  |  |  |  |  | http://www.bloodjournal.org/content/124/21/3734?sso-checked=true |
|  |  |  |  |  |  |  |
| IDH1 | R132C | Acute Myeloid Leukemia | 3A | AG-120 | 23393090, 23558169 | Hansen et al. Abstract# 3734, ASH 2014 |
|  |  |  |  |  |  | http://www.bloodjournal.org/content/124/21/3734?sso-checked=true |
|  |  |  |  |  |  |  |
|  |  |  |  |  |  | Matre et al. Abstract# 3763, ASH 2014 |
| IDH1 | R132G | All Tumors | 4 | BAY1436032, CB-839 | 28232670, 24523301 | http://www.bloodjournal.org/content/124/21/3763; Panknin et al. |
|  |  |  |  |  |  | Abstract# 2645, AACR 2016 |
|  |  |  |  |  |  |  |
|  |  |  |  |  |  | http://cancerres.aacrjournals.org/content/76/14_Supplement/2645 |
| IDH2 | R172M | All Liquid Tumors | 3A | AG-221 | 23558173 | Stein et al. Abstract# 115, ASH Annual Meeting 2014 |
|  |  |  |  |  |  | http://www.bloodjournal.org/content/124/21/115?sso-checked=true |
|  |  |  |  |  |  |  |
| IDH2 | R172K | All Liquid Tumors | 3A | AG-221 | 23558173 | Stein et al. Abstract# 115, ASH Annual Meeting 2014 |
|  |  |  |  |  |  | http://www.bloodjournal.org/content/124/21/115?sso-checked=true |
|  |  |  |  |  |  |  |
| IDH2 | R172S | All Liquid Tumors | 3A | AG-221 | 23558173 | Stein et al. Abstract# 115, ASH Annual Meeting 2014 |
|  |  |  |  |  |  | http://www.bloodjournal.org/content/124/21/115?sso-checked=true |
|  |  |  |  |  |  |  |
| IDH2 | R172G | All Liquid Tumors | 3A | AG-221 | 23558173 | Stein et al. Abstract# 115, ASH Annual Meeting 2014 |
|  |  |  |  |  |  | http://www.bloodjournal.org/content/124/21/115?sso-checked=true |
|  |  |  |  |  |  |  |
| IDH2 | R140Q | All Liquid Tumors | 3A | AG-221 | 23558173 | Stein et al. Abstract# 115, ASH Annual Meeting 2014 |
|  |  |  |  |  |  | http://www.bloodjournal.org/content/124/21/115?sso-checked=true |
|  |  |  |  |  |  |  |
| ARAF | S214C | Non-Small Cell Lung Cancer | 3A | Sorafenib | 24569458, 17016424 |  |
| ARAF | S214A | Histiocytosis | 3A | Sorafenib | 24569458, 17016424, 26566875 |  |
| MTOR | E2014K | Bladder Cancer | 3A | Everolimus | 24625776 |  |
| MTOR | F1888L | Renal Clear Cell Carcinoma | 4 | Temsirolimus, Rapamycin, Everolimus | 27482884 |  |
| MTOR | C1483F | Renal Clear Cell Carcinoma | 4 | Temsirolimus, Rapamycin, Everolimus | 27482884 |  |
| MTOR | S2215F | Renal Clear Cell Carcinoma | 4 | Temsirolimus, Rapamycin, Everolimus | 27482884 |  |
| MTOR | L2230V | Renal Clear Cell Carcinoma | 4 | Temsirolimus, Rapamycin, Everolimus | 27482884 |  |
| MTOR | T1977K | Renal Clear Cell Carcinoma | 4 | Temsirolimus, Rapamycin, Everolimus | 27482884 |  |
| ATM | Truncating Mutations | Prostate Cancer | 4 | Olaparib | 26510020 |  |
| ATM | N2875K | Prostate Cancer | 4 | Olaparib | 26510020 |  |
| MAP2K1 | Oncogenic Mutations | Histiocytic Disorder | 3A | Cobimetinib, Selumetinib, Trametinib | 23846731, 25351745, 22663011, 26324360, |  |
|  |  |  |  |  | 26566875, 25370473 |  |
|  |  |  |  |  |  |  |
| MAP2K1 | Oncogenic Mutations | Melanoma | 3A | Cobimetinib, Selumetinib, Trametinib | 23846731, 25351745, 22663011, 26324360, |  |
|  |  |  |  |  | 26566875, 25370473 |  |
|  |  |  |  |  |  |  |
| MAP2K1 | Oncogenic Mutations | Low-Grade Serous Ovarian Cancer | 3A | Cobimetinib, Selumetinib, Trametinib | 23846731, 25351745, 22663011, 26324360, |  |
|  |  |  |  |  | 26566875, 25370473 |  |
|  |  |  |  |  |  |  |
| MAP2K1 | Oncogenic Mutations | Non-Small Cell Lung Cancer | 3A | Cobimetinib, Selumetinib, Trametinib | 23846731, 25351745, 22663011, 26324360, |  |
|  |  |  |  |  | 26566875, 25370473 |  |
|  |  |  |  |  |  |  |
| PTCH1 | Truncating Mutations | Skin Cancer, Non-Melanoma | 3A | Sonidegib, Vismodegib | 24900187, 22670903, 22670904, 24523439 |  |
| PTCH1 | Truncating Mutations | Embryonal Tumor | 3A | Sonidegib | 24900187, 24523439 |  |
|  |  |  |  |  |  | Michalarea et al. Abstract# CT323, AACR 2015 |
|  |  |  |  |  | 26469692, 22588880, 24658109, 25544636, | http://www.abstractsonline.com/Plan/ViewAbstract.aspx?sKey=d60a28 |
|  |  |  |  |  |  | c6-91a3-4a37-a419-d79f96801b30&cKey=edbea616-0ae5-4520-936b- |
|  |  |  |  |  | 25673820, 22261591, 20166697, 18594509, |  |
| PTEN | Oncogenic Mutations | All Tumors | 4 | LY3023414, ARQ 751, Palbociclib + Gedato |  | 897b8d25b2d6&mKey=%7b19573A54-AE8F-4E00-9C23- |
|  |  |  |  |  | 23769634, 25398829, 21325073, 16647110, |  |
|  |  |  |  |  | 25177151, 24823695 | BD6D62268424%7d; Arkenau et al. Abstract# 2514, ASCO 2014 |
|  |  |  |  |  |  | http://hwmaint.meeting.ascopubs.org/cgi/content/abstract/32/15_suppl/ |
|  |  |  |  |  |  |  |
|  |  |  |  |  |  | 2514 |
| PTEN | Oncogenic Mutations | Prostate Cancer | 4 | LY3023414 + Enzalutamide | 21575859 |  |
| PTEN | Oncogenic Mutations | Endometrial Cancer | 4 | Olaparib | 20944090, 20049735, 20530668, 26187614, |  |
|  |  |  |  |  | 21468130 |  |
|  |  |  |  |  |  |  |
| JAK2 | PCM1-JAK2 Fusion | Leukemia | 3A | Ruxolitinib | 22899477, 25207766, 23630205, 22875628, |  |
|  |  |  |  |  | 25515960, 23400675, 25260694 |  |
|  |  |  |  |  |  |  |
| NF1 | Oncogenic Mutations | Glioblastoma | 4 | Trametinib | 26936308 |  |
| NF1 | Oncogenic Mutations | Neurofibroma | 4 | Binimetinib, PLX3397 | 26925841, 24718867 |  |
| NF1 | Oncogenic Mutations | Melanoma | 4 | Trametinib | 25243813, 24576830, 21245089, 24583796 |  |
| BRCA1 | Oncogenic Mutations | Ovarian Cancer | 1 | Rucaparib, Niraparib | 27908594, 27717299, 28229583 | McNeish, I. et al. Abstract# 5508, ASCO 2015 |
|  |  |  |  |  |  | http://meetinglibrary.asco.org/content/150121-156 |
|  |  |  |  |  |  |  |
| BRCA1 | Oncogenic Mutations | Ovarian Cancer | 2A | Olaparib | 19553641, 20406929, 21862407, 25366685, |  |
|  |  |  |  |  | 20609468 |  |
|  |  |  |  |  |  |  |
|  |  |  |  |  | 23846731, 22735384, 25265494, 25287827, |  |
| BRAF | V600K | Melanoma | 1 | b, Dabrafenib + Trametinib, Vemurafenib + | 25399551, 22608338, 23051966, 23918947, |  |
|  |  |  |  |  | 22663011, 20818844, 25265492, 24508103, |  |
|  |  |  |  |  |  |  |
|  |  |  |  |  | 23020132, 25089220 |  |
| BRAF | V600G | Colorectal Cancer | 3A | + Panitumumab, Encorafenib + Binimetinib | 19884556, 20619739, 25589621, 19001320, |  |
|  |  |  |  |  | 23617957, 22448344 |  |
|  |  |  |  |  |  |  |
| BRAF | V600E | Colorectal Cancer | 3A | + Panitumumab, Encorafenib + Binimetinib | 19884556, 20619739, 25589621, 19001320, |  |
|  |  |  |  |  | 23617957, 22448344 |  |
|  |  |  |  |  |  |  |
|  |  |  |  |  | 23846731, 22735384, 25265494, 25287827, |  |
| BRAF | V600M | Melanoma | 1 | rafenib, Dabrafenib + Trametinib, Vemurafe | 25399551, 22608338, 23051966, 23918947, |  |
|  |  |  |  |  | 22663011, 20818844, 25265492, 24508103, |  |
|  |  |  |  |  | 23020132, 25089220 |  |
| BRAF | V600D | Colorectal Cancer | 3A | + Panitumumab, Encorafenib + Binimetinib | 19884556, 20619739, 25589621, 19001320, |  |
|  |  |  |  |  | 23617957, 22448344 |  |
|  |  |  |  |  |  |  |
| BRAF | D594N | Melanoma | 4 | Trametinib | 26343582, 20141835 | Noeparast et al. Abstract# 11091, ASCO 2015 |
|  |  |  |  |  |  | http://meeting.ascopubs.org/cgi/content/abstract/33/15_suppl/11091 |
|  |  |  |  |  |  |  |
| BRAF | V600K | Colorectal Cancer | 3A | + Panitumumab, Encorafenib + Binimetinib | 19884556, 20619739, 25589621, 19001320, |  |
|  |  |  |  |  | 23617957, 22448344 |  |
|  |  |  |  |  |  |  |
| BRAF | D594E | Melanoma | 4 | Trametinib | 26343582, 20141835 | Noeparast et al. Abstract# 11091, ASCO 2015 |
|  |  |  |  |  |  | http://meeting.ascopubs.org/cgi/content/abstract/33/15_suppl/11091 |
|  |  |  |  |  |  |  |
| BRAF | L597V | Melanoma | 4 | BGB659 | 26343582 |  |
|  |  |  |  |  | 23846731, 22735384, 25265494, 25287827, |  |
| BRAF | V600R | Melanoma | 1 | rafenib, Dabrafenib + Trametinib, Vemurafe | 25399551, 22608338, 23051966, 23918947, |  |
|  |  |  |  |  | 22663011, 20818844, 25265492, 24508103, |  |
|  |  |  |  |  | 23020132, 25089220 |  |
| BRAF | K601E | Melanoma | 3A | Trametinib | 22663011, 22798288, 24933606, 23248257 |  |
| BRAF | V600D | Colorectal Cancer | 4 | Radiation + Trametinib + Fluorouracil | 23438367, 22663011 |  |
| BRAF | V600E | Colorectal Cancer | 4 | Radiation + Trametinib + Fluorouracil | 23438367, 22663011 |  |
| BRAF | Fusions | Ovarian Cancer | 3A | Selumetinib + Paclitaxel | 26324360 |  |
| BRAF | V600G | Colorectal Cancer | 4 | Radiation + Trametinib + Fluorouracil | 23438367, 22663011 |  |
| BRAF | V600K | Histiocytosis | 2A | Vemurafenib | 20818844, 26287849 |  |
| BRAF | L597Q | Melanoma | 3A | Trametinib | 22663011, 22798288, 24933606 |  |
| BRAF | V600R | Histiocytosis | 2A | Vemurafenib | 20818844, 26287849 |  |
| BRAF | G469A | Melanoma | 4 | Trametinib | 26343582, 20141835 | Noeparast et al. Abstract# 11091, ASCO 2015 |
|  |  |  |  |  |  | http://meeting.ascopubs.org/cgi/content/abstract/33/15_suppl/11091 |
|  |  |  |  |  |  |  |
|  |  |  |  |  | 23846731, 22735384, 25265494, 25287827, |  |
| BRAF | V600G | Melanoma | 1 | rafenib, Dabrafenib + Trametinib, Vemurafe | 25399551, 22608338, 23051966, 23918947, |  |
|  |  |  |  |  | 22663011, 20818844, 25265492, 24508103, |  |
|  |  |  |  |  | 23020132, 25089220 |  |
| BRAF | L597S | Melanoma | 3A | Trametinib | 22663011, 22798288, 24933606 |  |
| BRAF | V600D | Histiocytosis | 2A | Vemurafenib | 20818844, 26287849 |  |
| BRAF | KIAA1549-BRAF Fusion | Soft Tissue Sarcoma | 4 | Sorafenib + Temsirolimus | 23152448, 20586710, 20332142, 25662396, |  |
|  |  |  |  |  | 15466206, 24422672 |  |
|  |  |  |  |  |  |  |
|  |  |  |  |  | 27080216, 22663011, 20818844, 22743296, |  |
| BRAF | V600M | Non-Small Cell Lung Cancer | 2A | murafenib, Dabrafenib, Dabrafenib + Tramet | 27283860, 26776917, 25466451, 26287849, |  |
|  |  |  |  |  | 23733758, 26200454, 24888229, 25089220 |  |
| BRAF | V600M | Colorectal Cancer | 4 | Radiation + Trametinib + Fluorouracil | 23438367, 22663011 |  |
| BRAF | V600E | Histiocytosis | 2A | Vemurafenib | 20818844, 26287849 |  |
| BRAF | G596C | Melanoma | 4 | Trametinib | 26343582, 20141835 | Noeparast et al. Abstract# 11091, ASCO 2015 |
|  |  |  |  |  |  | http://meeting.ascopubs.org/cgi/content/abstract/33/15_suppl/11091 |
|  |  |  |  |  |  |  |
| BRAF | V600K | Colorectal Cancer | 4 | Radiation + Trametinib + Fluorouracil | 23438367, 22663011 |  |
| BRAF | V600G | Histiocytosis | 2A | Vemurafenib | 20818844, 26287849 |  |
| BRAF | L597V | Melanoma | 3A | Trametinib | 22663011, 22798288, 24933606 |  |
| BRAF | V600R | Colorectal Cancer | 4 | Radiation + Trametinib + Fluorouracil | 23438367, 22663011 |  |
|  |  |  |  |  | 23846731, 22735384, 25265494, 25287827, |  |
| BRAF | V600D | Melanoma | 1 | rafenib, Dabrafenib + Trametinib, Vemurafe | 25399551, 22608338, 23051966, 23918947, |  |
|  |  |  |  |  | 22663011, 20818844, 25265492, 24508103, |  |
|  |  |  |  |  | 23020132, 25089220 |  |
| BRAF | V600M | Histiocytosis | 2A | Vemurafenib | 20818844, 26287849 |  |

|  |  |  |  |  | 27080216, 22663011, 20818844, 22743296, |  |
| --- | --- | --- | --- | --- | --- | --- |
| BRAF | V600R | Non-Small Cell Lung Cancer | 2A | murafenib, Dabrafenib, Dabrafenib + Tramet | 27283860, 26776917, 25466451, 26287849, |  |
|  |  |  |  |  | 23733758, 26200454, 24888229, 25089220 |  |
|  |  |  |  |  | 27080216, 22663011, 20818844, 22743296, |  |
| BRAF | V600K | Non-Small Cell Lung Cancer | 2A | murafenib, Dabrafenib, Dabrafenib + Tramet | 27283860, 26776917, 25466451, 26287849, |  |
|  |  |  |  |  | 23733758, 26200454, 24888229, 25089220 |  |
|  |  |  |  |  | 27080216, 22663011, 20818844, 22743296, |  |
| BRAF | V600D | Non-Small Cell Lung Cancer | 2A | murafenib, Dabrafenib, Dabrafenib + Tramet | 27283860, 26776917, 25466451, 26287849, |  |
|  |  |  |  |  | 23733758, 26200454, 24888229, 25089220 |  |
|  |  |  |  |  | 27080216, 22663011, 20818844, 22743296, |  |
| BRAF | V600E | Non-Small Cell Lung Cancer | 2A | murafenib, Dabrafenib, Dabrafenib + Tramet | 27283860, 26776917, 25466451, 26287849, |  |
|  |  |  |  |  | 23733758, 26200454, 24888229, 25089220 |  |
| BRAF | G469V | Melanoma | 4 | Trametinib | 26343582, 20141835 | Noeparast et al. Abstract# 11091, ASCO 2015 |
|  |  |  |  |  |  | http://meeting.ascopubs.org/cgi/content/abstract/33/15_suppl/11091 |
|  |  |  |  |  |  |  |
| BRAF | L597R | Melanoma | 3A | Trametinib | 22663011, 22798288, 24933606 |  |
| BRAF | V600R | Colorectal Cancer | 3A | + Panitumumab, Encorafenib + Binimetinib | 19884556, 20619739, 25589621, 19001320, |  |
|  |  |  |  |  | 23617957, 22448344 |  |
|  |  |  |  |  |  |  |
|  |  |  |  |  | 23846731, 22735384, 25265494, 25287827, |  |
| BRAF | V600E | Melanoma | 1 | b, Dabrafenib + Trametinib, Vemurafenib + | 25399551, 22608338, 23051966, 23918947, |  |
|  |  |  |  |  | 22663011, 20818844, 25265492, 24508103, |  |
|  |  |  |  |  |  |  |
|  |  |  |  |  | 23020132, 25089220 |  |
|  |  |  |  |  | 27080216, 22663011, 20818844, 22743296, |  |
| BRAF | V600G | Non-Small Cell Lung Cancer | 2A | murafenib, Dabrafenib, Dabrafenib + Tramet | 27283860, 26776917, 25466451, 26287849, |  |
|  |  |  |  |  | 23733758, 26200454, 24888229, 25089220 |  |
| BRAF | L597Q | Melanoma | 4 | BGB659 | 26343582 |  |
| BRAF | G466V | Melanoma | 4 | Trametinib | 26343582, 20141835 | Noeparast et al. Abstract# 11091, ASCO 2015 |
|  |  |  |  |  |  | http://meeting.ascopubs.org/cgi/content/abstract/33/15_suppl/11091 |
|  |  |  |  |  |  |  |
| BRAF | V600M | Colorectal Cancer | 3A | + Panitumumab, Encorafenib + Binimetinib | 19884556, 20619739, 25589621, 19001320, |  |
|  |  |  |  |  | 23617957, 22448344 |  |
|  |  |  |  |  |  |  |
| BRCA2 | Oncogenic Mutations | Ovarian Cancer | 2A | Olaparib | 19553641, 20406929, 21862407, 25366685, |  |
|  |  |  |  |  | 20609468 |  |
|  |  |  |  |  |  |  |
| BRCA2 | Oncogenic Mutations | Ovarian Cancer | 1 | Rucaparib, Niraparib | 27908594, 27717299, 28229583 | McNeish, I. et al. Abstract# 5508, ASCO 2015 |
|  |  |  |  |  |  | http://meetinglibrary.asco.org/content/150121-156 |
|  |  |  |  |  |  |  |
| KIT | K642E | Thymic Tumor | 2A | Sorafenib | 19461405 |  |
| KIT | K558N | Gastrointestinal Stromal Tumor | 1 | Imatinib | 18235121, 12181401, 16098458, 15451219 | von Mehren et al. Abstract# 10016, ASCO 2011 |
|  |  |  |  |  |  | http://meetinglibrary.asco.org/content/82574-102 |
|  |  |  |  |  |  |  |
| KIT | E554_V559del | Gastrointestinal Stromal Tumor | 1 | Imatinib | 18235121, 12181401, 16098458, 15451219 | von Mehren et al. Abstract# 10016, ASCO 2011 |
|  |  |  |  |  |  | http://meetinglibrary.asco.org/content/82574-102 |
|  |  |  |  |  |  |  |
| KIT | H697Y | Gastrointestinal Stromal Tumor | 1 | Sunitinib | 17046465, 19282169, 25641662 |  |
|  |  |  |  |  |  | Trent et al. Abstract# 10006, ASCO 2011 |
| KIT | V559D | Gastrointestinal Stromal Tumor | 2A | Nilotinib, Dasatinib, Sorafenib | 22357255, 19467857, 23140824, 22270258, | http://meetinglibrary.asco.org/content/79120-102; Kindler et al. |
|  |  |  |  |  | 21456006, 17419150 | Abstract# 10009, ASCO 2011 |
|  |  |  |  |  |  |  |
|  |  |  |  |  |  | http://meetinglibrary.asco.org/content/80567-102 |
| KIT | V560G | Gastrointestinal Stromal Tumor | 1 | Imatinib | 18235121, 12181401, 16098458, 15451219 | von Mehren et al. Abstract# 10016, ASCO 2011 |
|  |  |  |  |  |  | http://meetinglibrary.asco.org/content/82574-102 |
|  |  |  |  |  |  |  |
| KIT | V559del | Gastrointestinal Stromal Tumor | 1 | Imatinib | 18235121, 12181401, 16098458, 15451219 | von Mehren et al. Abstract# 10016, ASCO 2011 |
|  |  |  |  |  |  | http://meetinglibrary.asco.org/content/82574-102 |
|  |  |  |  |  |  |  |
| KIT | N822H | Gastrointestinal Stromal Tumor | 1 | Imatinib | 18235121, 12181401, 16098458, 15451219 | von Mehren et al. Abstract# 10016, ASCO 2011 |
|  |  |  |  |  |  | http://meetinglibrary.asco.org/content/82574-102 |
|  |  |  |  |  |  |  |
| KIT | V559_V560del | Gastrointestinal Stromal Tumor | 1 | Imatinib | 18235121, 12181401, 16098458, 15451219 | von Mehren et al. Abstract# 10016, ASCO 2011 |
|  |  |  |  |  |  | http://meetinglibrary.asco.org/content/82574-102 |
|  |  |  |  |  |  |  |
| KIT | D820E | Thymic Tumor | 2A | Sorafenib | 19461405 |  |
| KIT | Exon 9 mutations | Gastrointestinal Stromal Tumor | 1 | Regorafenib, Sunitinib, Imatinib | 18235121, 12181401, 17046465, 19282169, | von Mehren et al. Abstract# 10016, ASCO 2011 |
|  |  |  |  |  | 25641662, 16098458, 15451219, 23177515 | http://meetinglibrary.asco.org/content/82574-102 |
|  |  |  |  |  |  |  |
| KIT | P577_D579del | Thymic Tumor | 2A | Sorafenib | 19461405 |  |
| KIT | D419del | Gastrointestinal Stromal Tumor | 1 | Imatinib | 18235121, 12181401, 16098458, 15451219 | von Mehren et al. Abstract# 10016, ASCO 2011 |
|  |  |  |  |  |  | http://meetinglibrary.asco.org/content/82574-102 |
|  |  |  |  |  |  |  |
| KIT | D816V | Gastrointestinal Stromal Tumor | 2A | Nilotinib | 22357255, 19467857, 21456006 |  |
| KIT | V560del | Gastrointestinal Stromal Tumor | 1 | Sunitinib, Imatinib | 18235121, 12181401, 17046465, 19282169, | von Mehren et al. Abstract# 10016, ASCO 2011 |
|  |  |  |  |  | 25641662, 16098458, 15451219 | http://meetinglibrary.asco.org/content/82574-102 |
|  |  |  |  |  |  |  |
| KIT | P551_M552del | Thymic Tumor | 2A | Sunitinib | 25592632, 20571495 |  |
| KIT | N822I | Gastrointestinal Stromal Tumor | 2A | Dasatinib | 17419150 | Trent et al. Abstract# 10006, ASCO 2011 |
|  |  |  |  |  |  | http://meetinglibrary.asco.org/content/79120-102 |
|  |  |  |  |  |  |  |
|  |  |  |  |  |  | Trent et al. Abstract# 10006, ASCO 2011 |
| KIT | D820G | Gastrointestinal Stromal Tumor | 2A | Nilotinib, Dasatinib, Sorafenib | 22357255, 19467857, 23140824, 22270258, | http://meetinglibrary.asco.org/content/79120-102; Kindler et al. |
|  |  |  |  |  | 21456006, 17419150 | Abstract# 10009, ASCO 2011 |
|  |  |  |  |  |  |  |
|  |  |  |  |  |  | http://meetinglibrary.asco.org/content/80567-102 |
| KIT | W557R | Gastrointestinal Stromal Tumor | 1 | Imatinib | 18235121, 12181401, 16098458, 15451219 | von Mehren et al. Abstract# 10016, ASCO 2011 |
|  |  |  |  |  |  | http://meetinglibrary.asco.org/content/82574-102 |
|  |  |  |  |  |  |  |
| KIT | H697Y | Thymic Tumor | 2A | Sunitinib | 25592632, 20571495 |  |
| KIT | M541L | Gastrointestinal Stromal Tumor | 1 | Imatinib | 18235121, 12181401, 16098458, 15451219 | von Mehren et al. Abstract# 10016, ASCO 2011 |
|  |  |  |  |  |  | http://meetinglibrary.asco.org/content/82574-102 |
|  |  |  |  |  |  |  |
| KIT | Exon 11 mutations | Thymic Tumor | 2A | Sunitinib | 25592632, 20571495 |  |
| KIT | D820A | Thymic Tumor | 2A | Sorafenib | 19461405 |  |
| KIT | D820Y | Thymic Tumor | 2A | Sorafenib | 19461405 |  |
| KIT | K558_V559del | Gastrointestinal Stromal Tumor | 1 | Imatinib | 18235121, 12181401, 16098458, 15451219 | von Mehren et al. Abstract# 10016, ASCO 2011 |
|  |  |  |  |  |  | http://meetinglibrary.asco.org/content/82574-102 |
|  |  |  |  |  |  |  |
| KIT | K550_W557del | Gastrointestinal Stromal Tumor | 1 | Regorafenib, Sunitinib, Imatinib | 18235121, 12181401, 17046465, 19282169, | von Mehren et al. Abstract# 10016, ASCO 2011 |
|  |  |  |  |  | 25641662, 16098458, 15451219, 23177515 | http://meetinglibrary.asco.org/content/82574-102 |
|  |  |  |  |  |  |  |
| KIT | D579del | Thymic Tumor | 2A | Sunitinib | 25592632, 20571495 |  |
| KIT | E554_I571del | Gastrointestinal Stromal Tumor | 1 | Imatinib | 18235121, 12181401, 16098458, 15451219 | von Mehren et al. Abstract# 10016, ASCO 2011 |
|  |  |  |  |  |  | http://meetinglibrary.asco.org/content/82574-102 |
|  |  |  |  |  |  |  |
| KIT | V559D | Gastrointestinal Stromal Tumor | 1 | Imatinib | 18235121, 12181401, 16098458, 15451219 | von Mehren et al. Abstract# 10016, ASCO 2011 |
|  |  |  |  |  |  | http://meetinglibrary.asco.org/content/82574-102 |
|  |  |  |  |  |  |  |
| KIT | E554_K558del | Thymic Tumor | 2A | Sunitinib | 25592632, 20571495 |  |
| KIT | L576P | Gastrointestinal Stromal Tumor | 1 | Sunitinib, Imatinib | 18235121, 12181401, 17046465, 19282169, | von Mehren et al. Abstract# 10016, ASCO 2011 |
|  |  |  |  |  | 25641662, 16098458, 15451219 | http://meetinglibrary.asco.org/content/82574-102 |
|  |  |  |  |  |  |  |
| KIT | K550_W557del | Thymic Tumor | 2A | Sunitinib | 25592632, 20571495 |  |
| KIT | A502_Y503dup | Gastrointestinal Stromal Tumor | 1 | Sunitinib | 17046465, 19282169, 25641662 |  |
| KIT | N564_Y578del | Gastrointestinal Stromal Tumor | 1 | Imatinib | 18235121, 12181401, 16098458, 15451219 | von Mehren et al. Abstract# 10016, ASCO 2011 |
|  |  |  |  |  |  | http://meetinglibrary.asco.org/content/82574-102 |
|  |  |  |  |  |  |  |
| KIT | Exon 11 mutations | Gastrointestinal Stromal Tumor | 1 | Regorafenib, Sunitinib, Imatinib | 18235121, 12181401, 17046465, 19282169, | von Mehren et al. Abstract# 10016, ASCO 2011 |
|  |  |  |  |  | 25641662, 16098458, 15451219, 23177515 | http://meetinglibrary.asco.org/content/82574-102 |
|  |  |  |  |  |  |  |
| KIT | P838L | Gastrointestinal Stromal Tumor | 1 | Imatinib | 18235121, 12181401, 16098458, 15451219 | von Mehren et al. Abstract# 10016, ASCO 2011 |
|  |  |  |  |  |  | http://meetinglibrary.asco.org/content/82574-102 |
|  |  |  |  |  |  |  |
| KIT | Y570H | Gastrointestinal Stromal Tumor | 1 | Imatinib | 18235121, 12181401, 16098458, 15451219 | von Mehren et al. Abstract# 10016, ASCO 2011 |
|  |  |  |  |  |  | http://meetinglibrary.asco.org/content/82574-102 |
|  |  |  |  |  |  |  |
| KIT | T574insTQLPYD | Gastrointestinal Stromal Tumor | 1 | Imatinib | 18235121, 12181401, 16098458, 15451219 | von Mehren et al. Abstract# 10016, ASCO 2011 |
|  |  |  |  |  |  | http://meetinglibrary.asco.org/content/82574-102 |
|  |  |  |  |  |  |  |
| KIT | P573_D579del | Gastrointestinal Stromal Tumor | 1 | Imatinib | 18235121, 12181401, 16098458, 15451219 | von Mehren et al. Abstract# 10016, ASCO 2011 |
|  |  |  |  |  |  | http://meetinglibrary.asco.org/content/82574-102 |
|  |  |  |  |  |  |  |
| KIT | V559D | Thymic Tumor | 2A | Sorafenib | 19461405 |  |
| KIT | K642E | Melanoma | 2A | Imatinib | 23775962, 18235121, 12181401, 21642685, |  |
|  |  |  |  |  | 16098458, 15451219 |  |
|  |  |  |  |  |  |  |
| KIT | L576P | Thymic Tumor | 2A | Sunitinib | 25592632, 20571495 |  |
| KIT | V555_V559del | Gastrointestinal Stromal Tumor | 1 | Imatinib | 18235121, 12181401, 16098458, 15451219 | von Mehren et al. Abstract# 10016, ASCO 2011 |
|  |  |  |  |  |  | http://meetinglibrary.asco.org/content/82574-102 |
|  |  |  |  |  |  |  |
| KIT | K558delinsNP | Gastrointestinal Stromal Tumor | 1 | Regorafenib, Sunitinib, Imatinib | 18235121, 12181401, 17046465, 19282169, | von Mehren et al. Abstract# 10016, ASCO 2011 |
|  |  |  |  |  | 25641662, 16098458, 15451219, 23177515 | http://meetinglibrary.asco.org/content/82574-102 |
|  |  |  |  |  |  |  |
| KIT | D579del | Gastrointestinal Stromal Tumor | 1 | Sunitinib, Imatinib | 18235121, 12181401, 17046465, 19282169, | von Mehren et al. Abstract# 10016, ASCO 2011 |
|  |  |  |  |  | 25641662, 16098458, 15451219 | http://meetinglibrary.asco.org/content/82574-102 |
|  |  |  |  |  |  |  |
| KIT | V560G | Gastrointestinal Stromal Tumor | 2A | Dasatinib | 17419150 | Trent et al. Abstract# 10006, ASCO 2011 |
|  |  |  |  |  |  | http://meetinglibrary.asco.org/content/79120-102 |
|  |  |  |  |  |  |  |
| KIT | Y553_K558del | Gastrointestinal Stromal Tumor | 1 | Imatinib | 18235121, 12181401, 16098458, 15451219 | von Mehren et al. Abstract# 10016, ASCO 2011 |
|  |  |  |  |  |  | http://meetinglibrary.asco.org/content/82574-102 |
|  |  |  |  |  |  |  |
| KIT | V560del | Thymic Tumor | 2A | Sunitinib | 25592632, 20571495 |  |
| KIT | D816Y | Gastrointestinal Stromal Tumor | 2A | Dasatinib | 17419150 | Trent et al. Abstract# 10006, ASCO 2011 |
|  |  |  |  |  |  | http://meetinglibrary.asco.org/content/79120-102 |
|  |  |  |  |  |  |  |
| KIT | V555_L576del | Gastrointestinal Stromal Tumor | 2A | Nilotinib | 22357255, 19467857, 21456006 |  |
| KIT | D820Y | Gastrointestinal Stromal Tumor | 1 | Regorafenib | 23177515 |  |
| KIT | N822K | Gastrointestinal Stromal Tumor | 1 | Regorafenib | 23177515 |  |
| KIT | V560D | Gastrointestinal Stromal Tumor | 1 | Regorafenib, Sunitinib, Imatinib | 18235121, 12181401, 17046465, 19282169, | von Mehren et al. Abstract# 10016, ASCO 2011 |
|  |  |  |  |  | 25641662, 16098458, 15451219, 23177515 | http://meetinglibrary.asco.org/content/82574-102 |
|  |  |  |  |  |  |  |
| KIT | N822Y | Gastrointestinal Stromal Tumor | 1 | Imatinib | 18235121, 12181401, 16098458, 15451219 | von Mehren et al. Abstract# 10016, ASCO 2011 |
|  |  |  |  |  |  | http://meetinglibrary.asco.org/content/82574-102 |
|  |  |  |  |  |  |  |
| KIT | P551_M552del | Gastrointestinal Stromal Tumor | 1 | Sunitinib | 17046465, 19282169, 25641662 |  |
| KIT | V530I | Gastrointestinal Stromal Tumor | 1 | Imatinib | 18235121, 12181401, 16098458, 15451219 | von Mehren et al. Abstract# 10016, ASCO 2011 |
|  |  |  |  |  |  | http://meetinglibrary.asco.org/content/82574-102 |
|  |  |  |  |  |  |  |
| KIT | T417_D419delinsRG | Gastrointestinal Stromal Tumor | 1 | Imatinib | 18235121, 12181401, 16098458, 15451219 | von Mehren et al. Abstract# 10016, ASCO 2011 |
|  |  |  |  |  |  | http://meetinglibrary.asco.org/content/82574-102 |
|  |  |  |  |  |  |  |
| KIT | W557_K558del | Gastrointestinal Stromal Tumor | 1 | Imatinib | 18235121, 12181401, 16098458, 15451219 | von Mehren et al. Abstract# 10016, ASCO 2011 |
|  |  |  |  |  |  | http://meetinglibrary.asco.org/content/82574-102 |
|  |  |  |  |  |  |  |
| KIT | I563_L576del | Gastrointestinal Stromal Tumor | 1 | Imatinib | 18235121, 12181401, 16098458, 15451219 | von Mehren et al. Abstract# 10016, ASCO 2011 |
|  |  |  |  |  |  | http://meetinglibrary.asco.org/content/82574-102 |
|  |  |  |  |  |  |  |
| KIT | P577_D579del | Gastrointestinal Stromal Tumor | 2A | Sorafenib | 23140824, 22270258 | Kindler et al. Abstract# 10009, ASCO 2011 |
|  |  |  |  |  |  | http://meetinglibrary.asco.org/content/80567-102 |
|  |  |  |  |  |  |  |
| KIT | I653T | Gastrointestinal Stromal Tumor | 1 | Imatinib | 18235121, 12181401, 16098458, 15451219 | von Mehren et al. Abstract# 10016, ASCO 2011 |
|  |  |  |  |  |  | http://meetinglibrary.asco.org/content/82574-102 |
|  |  |  |  |  |  |  |
| KIT | Q556_K558del | Gastrointestinal Stromal Tumor | 1 | Imatinib | 18235121, 12181401, 16098458, 15451219 | von Mehren et al. Abstract# 10016, ASCO 2011 |
|  |  |  |  |  |  | http://meetinglibrary.asco.org/content/82574-102 |
|  |  |  |  |  |  |  |
| KIT | Exon 9 mutations | Thymic Tumor | 2A | Sunitinib | 25592632, 20571495 |  |
| KIT | T417_D419delinsI | Gastrointestinal Stromal Tumor | 1 | Imatinib | 18235121, 12181401, 16098458, 15451219 | von Mehren et al. Abstract# 10016, ASCO 2011 |
|  |  |  |  |  |  | http://meetinglibrary.asco.org/content/82574-102 |
|  |  |  |  |  |  |  |
| KIT | V654A | Gastrointestinal Stromal Tumor | 2A | Nilotinib | 22357255, 19467857, 21456006 |  |
| KIT | V569_L576del | Gastrointestinal Stromal Tumor | 1 | Imatinib | 18235121, 12181401, 16098458, 15451219 | von Mehren et al. Abstract# 10016, ASCO 2011 |
|  |  |  |  |  |  | http://meetinglibrary.asco.org/content/82574-102 |
|  |  |  |  |  |  |  |
| KIT | P577_W582delinsPYD | Gastrointestinal Stromal Tumor | 1 | Imatinib | 18235121, 12181401, 16098458, 15451219 | von Mehren et al. Abstract# 10016, ASCO 2011 |
|  |  |  |  |  |  | http://meetinglibrary.asco.org/content/82574-102 |
|  |  |  |  |  |  |  |
| KIT | V654A | Thymic Tumor | 2A | Sunitinib | 25592632, 20571495 |  |
| KIT | V555_L576del | Gastrointestinal Stromal Tumor | 1 | Sunitinib, Imatinib | 18235121, 12181401, 17046465, 19282169, | von Mehren et al. Abstract# 10016, ASCO 2011 |
|  |  |  |  |  | 25641662, 16098458, 15451219 | http://meetinglibrary.asco.org/content/82574-102 |
|  |  |  |  |  |  |  |
| KIT | F522C | Gastrointestinal Stromal Tumor | 1 | Imatinib | 18235121, 12181401, 16098458, 15451219 | von Mehren et al. Abstract# 10016, ASCO 2011 |
|  |  |  |  |  |  | http://meetinglibrary.asco.org/content/82574-102 |
|  |  |  |  |  |  |  |
| KIT | D820E | Gastrointestinal Stromal Tumor | 2A | Sorafenib | 23140824, 22270258 | Kindler et al. Abstract# 10009, ASCO 2011 |
|  |  |  |  |  |  | http://meetinglibrary.asco.org/content/80567-102 |
|  |  |  |  |  |  |  |

| KIT | L576P | Melanoma | 2A | Imatinib | 23775962, 18235121, 12181401, 21642685, |  |
| --- | --- | --- | --- | --- | --- | --- |
|  |  |  |  |  | 16098458, 15451219 |  |
|  |  |  |  |  |  |  |
| KIT | V654A | Gastrointestinal Stromal Tumor | 1 | Sunitinib | 17046465, 19282169, 25641662 |  |
| KIT | V559C | Gastrointestinal Stromal Tumor | 1 | Imatinib | 18235121, 12181401, 16098458, 15451219 | von Mehren et al. Abstract# 10016, ASCO 2011 |
|  |  |  |  |  |  | http://meetinglibrary.asco.org/content/82574-102 |
|  |  |  |  |  |  |  |
| KIT | K558delinsNP | Thymic Tumor | 2A | Sunitinib | 25592632, 20571495 |  |
| KIT | D820G | Gastrointestinal Stromal Tumor | 1 | Regorafenib, Sunitinib | 17046465, 19282169, 25641662, 23177515 |  |
|  |  |  |  |  | 22357255, 19467857, 23140824, 22270258, | Kindler et al. Abstract# 10009, ASCO 2011 |
| KIT | D820Y | Gastrointestinal Stromal Tumor | 2A | Nilotinib, Dasatinib, Sorafenib |  | http://meetinglibrary.asco.org/content/80567-102; Trent et al. Abstract# |
|  |  |  |  |  | 21456006, 17419150 |  |
|  |  |  |  |  |  | 10006, ASCO 2011 http://meetinglibrary.asco.org/content/79120-102 |
|  |  |  |  |  |  |  |
| KIT | D820A | Gastrointestinal Stromal Tumor | 2A | Nilotinib, Sorafenib | 22357255, 19467857, 23140824, 22270258, | Kindler et al. Abstract# 10009, ASCO 2011 |
|  |  |  |  |  | 21456006 | http://meetinglibrary.asco.org/content/80567-102 |
|  |  |  |  |  |  |  |
| KIT | W557_K558del | Thymic Tumor | 2A | Sorafenib | 19461405 |  |
| KIT | Y553N | Gastrointestinal Stromal Tumor | 1 | Imatinib | 18235121, 12181401, 16098458, 15451219 | von Mehren et al. Abstract# 10016, ASCO 2011 |
|  |  |  |  |  |  | http://meetinglibrary.asco.org/content/82574-102 |
|  |  |  |  |  |  |  |
| KIT | N505I | Thymic Tumor | 2A | Sorafenib | 19461405 |  |
| KIT | E554_K558del | Gastrointestinal Stromal Tumor | 1 | Sunitinib, Imatinib | 18235121, 12181401, 17046465, 19282169, | von Mehren et al. Abstract# 10016, ASCO 2011 |
|  |  |  |  |  | 25641662, 16098458, 15451219 | http://meetinglibrary.asco.org/content/82574-102 |
|  |  |  |  |  |  |  |
| KIT | K642E | Gastrointestinal Stromal Tumor | 1 | Imatinib | 18235121, 12181401, 16098458, 15451219 | von Mehren et al. Abstract# 10016, ASCO 2011 |
|  |  |  |  |  |  | http://meetinglibrary.asco.org/content/82574-102 |
|  |  |  |  |  |  |  |
| KIT | Y578C | Gastrointestinal Stromal Tumor | 1 | Imatinib | 18235121, 12181401, 16098458, 15451219 | von Mehren et al. Abstract# 10016, ASCO 2011 |
|  |  |  |  |  |  | http://meetinglibrary.asco.org/content/82574-102 |
|  |  |  |  |  |  |  |
| KIT | V555_L576del | Thymic Tumor | 2A | Sunitinib | 25592632, 20571495 |  |
| KIT | A502_Y503dup | Thymic Tumor | 2A | Sunitinib | 25592632, 20571495 |  |
| KIT | V559A | Melanoma | 2A | Imatinib | 23775962, 18235121, 12181401, 21642685, |  |
|  |  |  |  |  | 16098458, 15451219 |  |
|  |  |  |  |  |  |  |
|  |  |  |  |  | 22357255, 19467857, 23140824, 22270258, | Kindler et al. Abstract# 10009, ASCO 2011 |
| KIT | W557_K558del | Gastrointestinal Stromal Tumor | 2A | Nilotinib, Dasatinib, Sorafenib |  | http://meetinglibrary.asco.org/content/80567-102; Trent et al. Abstract# |
|  |  |  |  |  | 21456006, 17419150 |  |
|  |  |  |  |  |  | 10006, ASCO 2011 http://meetinglibrary.asco.org/content/79120-102 |
|  |  |  |  |  |  |  |
| KIT | D820G | Thymic Tumor | 2A | Sunitinib, Sorafenib | 19461405, 25592632, 20571495 |  |
| KIT | W557G | Gastrointestinal Stromal Tumor | 1 | Imatinib | 18235121, 12181401, 16098458, 15451219 | von Mehren et al. Abstract# 10016, ASCO 2011 |
|  |  |  |  |  |  | http://meetinglibrary.asco.org/content/82574-102 |
|  |  |  |  |  |  |  |
| KIT | D816F | Gastrointestinal Stromal Tumor | 2A | Dasatinib | 17419150 | Trent et al. Abstract# 10006, ASCO 2011 |
|  |  |  |  |  |  | http://meetinglibrary.asco.org/content/79120-102 |
|  |  |  |  |  |  |  |
| KIT | K642E | Gastrointestinal Stromal Tumor | 2A | Nilotinib, Sorafenib | 22357255, 19467857, 23140824, 22270258, | Kindler et al. Abstract# 10009, ASCO 2011 |
|  |  |  |  |  | 21456006 | http://meetinglibrary.asco.org/content/80567-102 |
|  |  |  |  |  |  |  |
| KIT | V560D | Thymic Tumor | 2A | Sunitinib | 25592632, 20571495 |  |
| KIT | M552_W557del | Gastrointestinal Stromal Tumor | 1 | Imatinib | 18235121, 12181401, 16098458, 15451219 | von Mehren et al. Abstract# 10016, ASCO 2011 |
|  |  |  |  |  |  | http://meetinglibrary.asco.org/content/82574-102 |
|  |  |  |  |  |  |  |
| KIT | L576P | Gastrointestinal Stromal Tumor | 2A | Nilotinib, Dasatinib | 22357255, 19467857, 21456006, 17419150 | Trent et al. Abstract# 10006, ASCO 2011 |
|  |  |  |  |  |  | http://meetinglibrary.asco.org/content/79120-102 |
|  |  |  |  |  |  |  |
| KIT | V559C | Gastrointestinal Stromal Tumor | 2A | Nilotinib | 22357255, 19467857, 21456006 |  |
| KIT | K558_E562del | Gastrointestinal Stromal Tumor | 1 | Imatinib | 18235121, 12181401, 16098458, 15451219 | von Mehren et al. Abstract# 10016, ASCO 2011 |
|  |  |  |  |  |  | http://meetinglibrary.asco.org/content/82574-102 |
|  |  |  |  |  |  |  |
| KIT | N505I | Gastrointestinal Stromal Tumor | 2A | Sorafenib | 23140824, 22270258 | Kindler et al. Abstract# 10009, ASCO 2011 |
|  |  |  |  |  |  | http://meetinglibrary.asco.org/content/80567-102 |
|  |  |  |  |  |  |  |
| KIT | V559G | Gastrointestinal Stromal Tumor | 1 | Imatinib | 18235121, 12181401, 16098458, 15451219 | von Mehren et al. Abstract# 10016, ASCO 2011 |
|  |  |  |  |  |  | http://meetinglibrary.asco.org/content/82574-102 |
|  |  |  |  |  |  |  |
| KRAS | Wildtype | Colorectal Cancer | 4 | anitumumab, Pembrolizumab, Regorafenib | 26288737, 25242168, 23177514, 26028255, | Strickler et al. Abstract# 3548, ASCO 2016 |
|  |  |  |  |  | 21170960 | http://meetinglibrary.asco.org/content/166423-176 |
|  |  |  |  |  |  | Germann et al. Abstract# 4693, AACR 2015 |
| KRAS | Oncogenic Mutations | Colorectal Cancer | 4 | py + Trametinib + Fluorouracil, Cobimetinib | 21690569, 23438367, 23846731, 22663011, | http://cancerres.aacrjournals.org/content/75/15_Supplement/4693; |
|  |  |  |  |  | 25787767, 26515496, 25609064, 26028255 | Bendell et al. Abstract# 3502, ASCO 2016 |
|  |  |  |  |  |  |  |
|  |  |  |  |  |  | http://meetinglibrary.asco.org/content/171295-176 |
|  |  |  |  |  | 25801412, 23934108, 22805291, 26728409, |  |
| KRAS | Oncogenic Mutations | Non-Small Cell Lung Cancer | 4 | lumetinib, Binimetinib, Ribociclib + Trametin | 24908424, 26837474, 20609353, 23200175, |  |
|  |  |  |  |  | 15542782, 24746704, 25792301, 15801831 |  |
|  |  |  |  |  | 25037139, 22805291, 20699365, 20609351, | Robarge et al. Abstract# DDT02-03, AACR 2014 |
|  |  |  |  |  |  | http://cancerres.aacrjournals.org/content/74/19_Supplement/DDT02- |
| KRAS | Oncogenic Mutations | All Tumors | 4 | nimetinib + Alpelisib, Cobimetinib + GDC-09 | 15483017, 25265494, 25342139, 23726034, |  |
|  |  |  |  |  |  | 03; Juric et al. Abstract# 9051, ASCO 2014 |
|  |  |  |  |  | 24569456, 23587417 |  |
|  |  |  |  |  |  | http://meetinglibrary.asco.org/content/129278-144 |
|  |  |  |  |  |  |  |
| KRAS | Wildtype | Colorectal Cancer | 1 | Regorafenib, Panitumumab, Cetuximab | 20921462, 24739896, 20921465, 19339720, |  |
|  |  |  |  |  | 15269313, 15677699, 17470858, 23177514 |  |
|  |  |  |  |  |  |  |
| KRAS | Oncogenic Mutations | Colorectal Cancer | R1 | Panitumumab, Cetuximab | 21228335, 20619739, 18316791, 20921465, |  |
|  |  |  |  |  | 24024839 |  |
|  |  |  |  |  |  |  |
| RAF1 | S257L | Lung Adenocarcinoma | 4 | Sorafenib | 24569458 |  |
| NRAS | Oncogenic Mutations | Melanoma | 3A | Binimetinib, Binimetinib + Ribociclib | 23414587, 16273091, 22983396 | Sosman et al. Abstract #9009, ASCO 2014 |
|  |  |  |  |  |  | http://meetinglibrary.asco.org/content/130034-144 |
|  |  |  |  |  |  |  |
| NRAS | Oncogenic Mutations | Colorectal Cancer | R1 | Panitumumab, Cetuximab | 20619739, 24024844, 25110411, 24024839 |  |
| NRAS | Oncogenic Mutations | Thyroid Cancer | 3A | Selumetinib + Radioiodine Uptake Therapy | 22105174, 23406027 |  |
| NRAS | Oncogenic Mutations | Colorectal Cancer | 3A | Cobimetinib + Atezolizumab | 25787767, 26515496, 25609064, 26028255 | Bendell et al. Abstract# 3502, ASCO 2016 |
|  |  |  |  |  |  | http://meetinglibrary.asco.org/content/171295-176 |
|  |  |  |  |  |  |  |
| NRAS | Oncogenic Mutations | Colorectal Cancer | 4 | adiation Therapy + Trametinib + Fluorourac | 21690569, 23438367, 23846731, 22663011 | Germann et al. Abstract# 4693, AACR 2015 |
|  |  |  |  |  |  | http://cancerres.aacrjournals.org/content/75/15_Supplement/4693 |
|  |  |  |  |  |  |  |
| NTRK1 | Fusions | All Tumors | 3A | LOXO-101 | 26216294, 26884591, 26603524 |  |
| NTRK1 | Fusions | Salivary Gland Cancer | 3A | Entrectinib | 26565381, 26884591, 26457764, 26797418 |  |
| NTRK2 | Fusions | Salivary Gland Cancer | 3A | LOXO-101, Entrectinib | 26565381, 26216294, 26884591, 26457764, |  |
|  |  |  |  |  | 26797418, 26603524 |  |
|  |  |  |  |  |  |  |
| NTRK3 | Fusions | Salivary Gland Cancer | 3A | LOXO-101, Entrectinib | 26565381, 26216294, 26884591, 26457764, |  |
|  |  |  |  |  | 26797418, 27093299, 26603524 |  |
|  |  |  |  |  |  |  |
| RET | Fusions | Non-Small Cell Lung Cancer | 3A | Vandetanib | 23578175, 25366691, 23584301, 23991695, |  |
|  |  |  |  |  | 23154560 |  |
|  |  |  |  |  |  |  |
| RET | Fusions | Non-Small Cell Lung Cancer | 2A | Cabozantinib | 23533264 | Drilon et al. Abstract# 8007, ASCO 2015 |
|  |  |  |  |  |  | http://meetinglibrary.asco.org/content/147349-156 |
|  |  |  |  |  |  |  |
